# Supplementary material for: Sulphostin-inspired N-phosphonopiperidones as selective covalent DPP8 and DPP9 inhibitors
Source: Nat Commun. 2025 Apr 3;16:3208. doi: 10.1038/s41467-025-58493-z (PMC11968843; doi:10.1038/s41467-025-58493-z)
Supplement: Supplementary file 1 — Supplementary Information [file 41467_2025_58493_MOESM1_ESM.pdf]

Supplementary Information

Sulphostin-inspired *N*-phosphonopiperidones as selective covalent DPP8 and DPP9 inhibitors

Leonard Sewald[#], Werner W. A. Tabak[#], Lorenz Fehr[#], Samuel Zolg, Maja Najdzion, Carlo J. A. Verhoef, David Podlesainski, Ruth Geiss-Friedlander, Alfred Lammens, Farnusch Kaschani, Doris Hellerschmied, Robert Huber, Markus Kaiser*

*Corresponding author

[#]These authors contributed equally: Leonard Sewald, Werner W. A. Tabak, Lorenz Fehr

Email: markus.kaiser@uni-due.de

Contents

Supplementary Figures ... 3

Supplementary Tables ... 17

Supplementary Methods ... 20

- Chemical Synthesis ... 20
  - General Information ... 20
  - Synthetic Procedures ... 21
- NMR Spectra ... 35

Supplementary References ... 66

Supplementary Figures

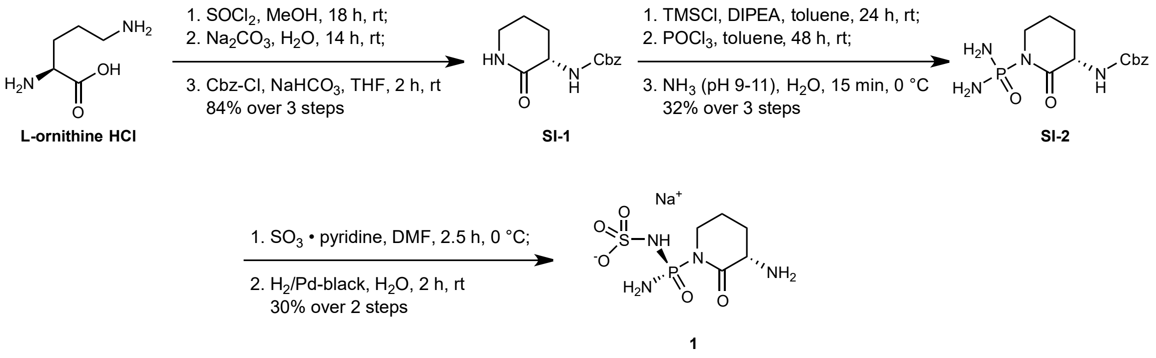

Supplementary Figure 1. **Chemical synthesis of Sulphostin.** Procedure adapted from Abe *et al.*[1,2].

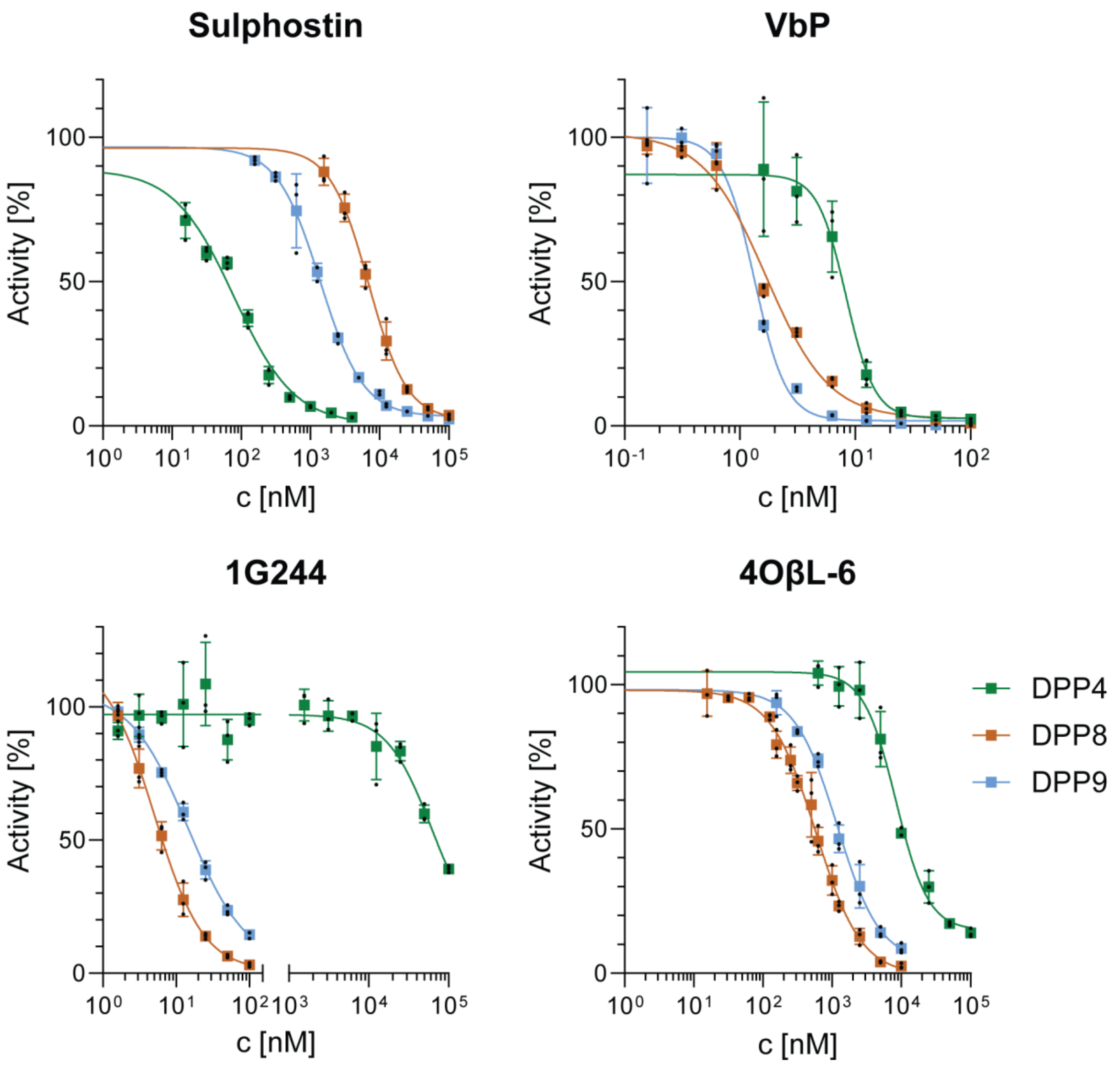

Supplementary Figure 2. **DPP4/8/9 inhibition curves of Sulphostin, VbP, 1G244, and 4OβL-6.** Enzyme activity was determined by measuring the release of AMC from the substrate GP-AMC. All activity measurements were performed in triplicate (*n* = 3 technical replicates), mean values are shown and error bars indicate the standard error of the mean (SEM). Data was normalized to the DMSO control. Source data are provided as a Source Data file.

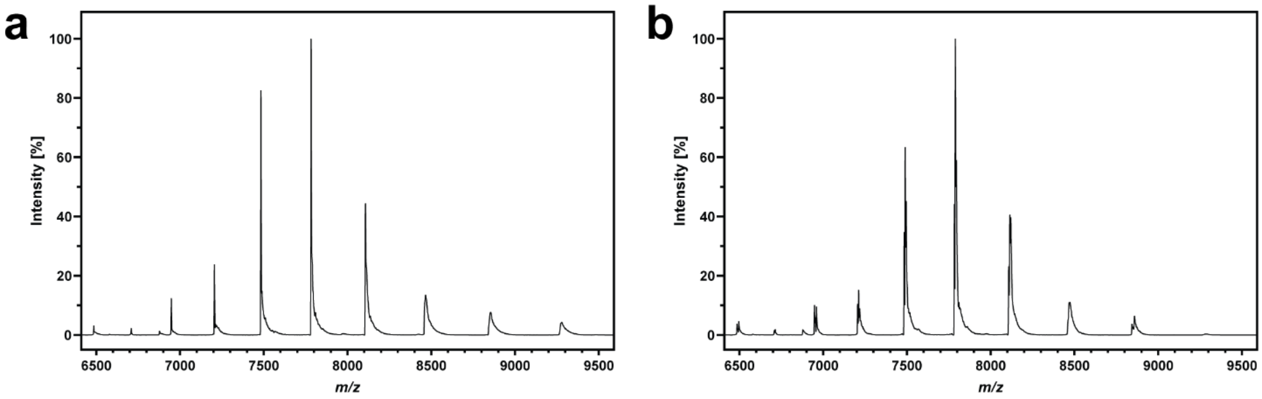

Supplementary Figure 3. **Native MS spectrum of DPP9 with or without sulphostin bound.** (**a**) Native MS spectrum of DPP9 in the dimeric state after treatment with DMSO. Peaks correspond to different charge states. (**b**) Native MS spectrum of DPP9 in the dimeric state after pre-incubation with 50 µM Sulphostin for 90 min. Peaks correspond to different charge states.

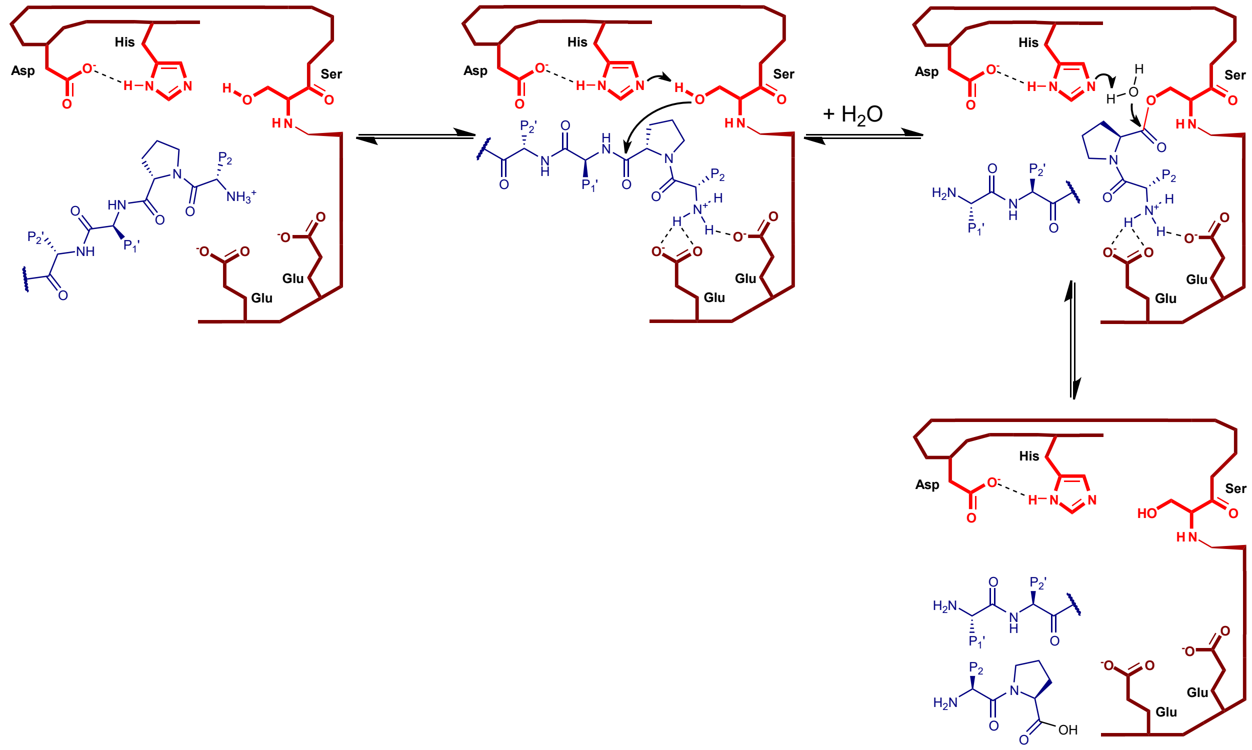

Supplementary Figure 4. **Reaction mechanism of peptide cleavage by DPP9.** The catalytic residues are shown in red and the substrate in blue. The histidine residue of the catalytic triad deprotonates the active site serine, which attacks the carbonyl carbon atom of the substrate in a nucleophilic addition. P1‘-P2’ of the substrate acts as a leaving group and is released by an elimination reaction. The N-terminal site remains and leads to an acyl intermediate whose carbonyl carbon is attacked by water, which is deprotonated by the histidine residue of the catalytic triad. The acyl bond is then cleaved by an elimination reaction, resulting in the release of the product.

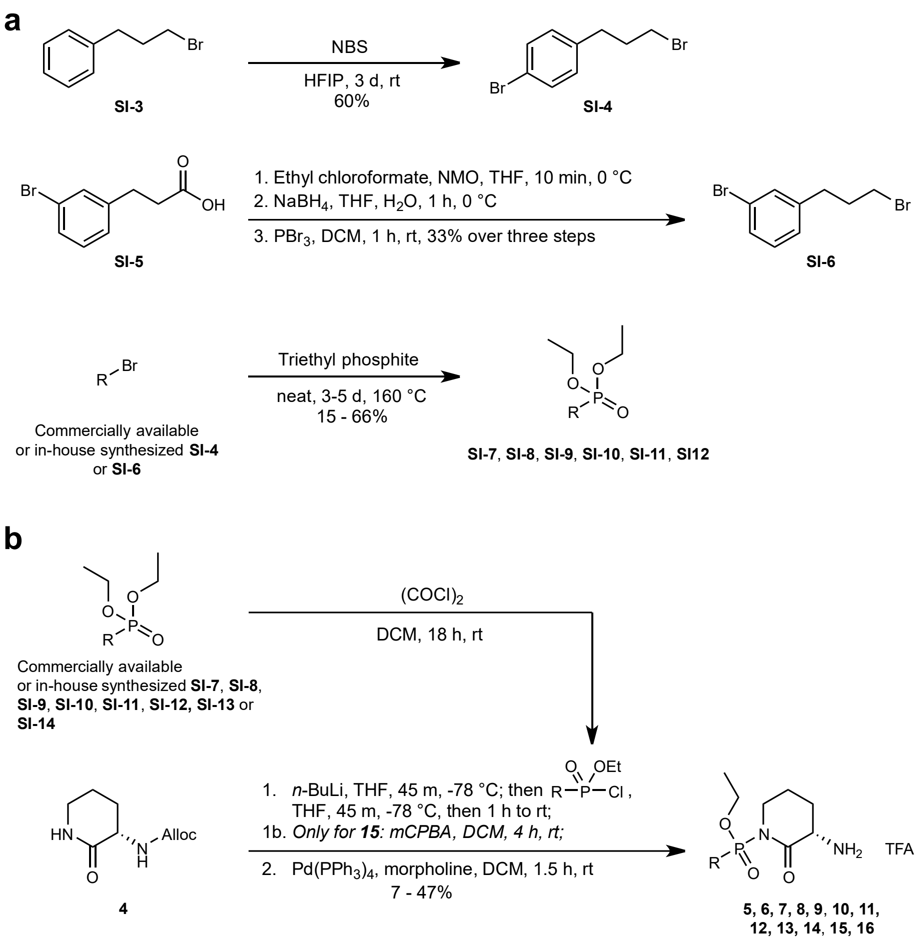

Supplementary Figure 5. **Chemical synthesis of *N*-phosphono-(*S*)-aminopiperidine-2-ones 5, 6, 7, 8, 9, 10, 11, 12, 13, 14, 15, and 16.** Chemical synthesis of (**a**) phosphonate diethyl ester derivatives and (**b**) *N*-phosphono-(*S*)-aminopiperidine-2-ones.

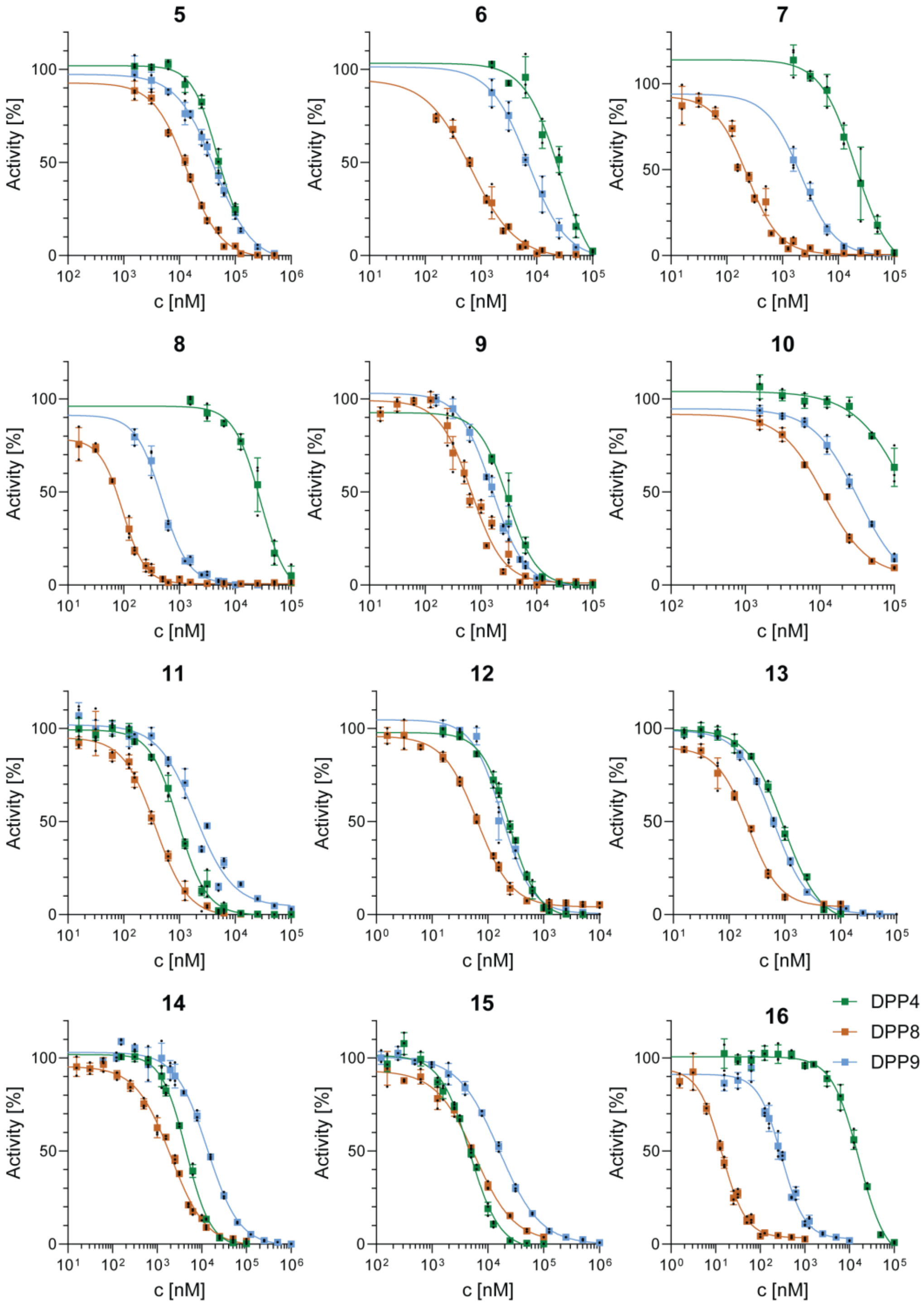

Supplementary Figure 6. **DPP4/8/9 inhibition curves of the *N*-phosphono-(*S*)-3-aminopiperidine-2-ones.** Enzyme activity was determined by measuring the release of AMC from the substrate GP-AMC. All activity measurements were performed in triplicate (*n* = 3 technical replicates), mean values are shown and error bars indicate the SEM. Data was normalized to the DMSO control. Source data are provided as a Source Data file.

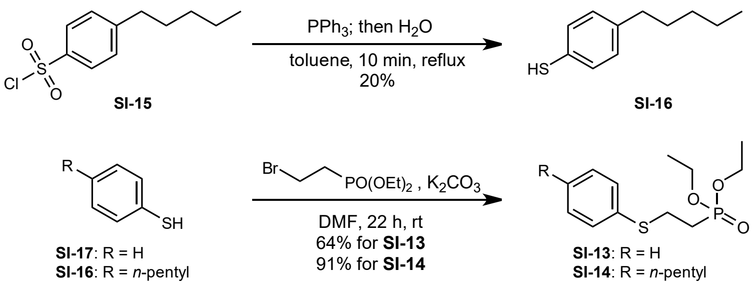

Supplementary Figure 7. **Chemical synthesis of SI-13 and SI-14 used for the synthesis of the sulfone derivative 15 and its precursor 14.**

5

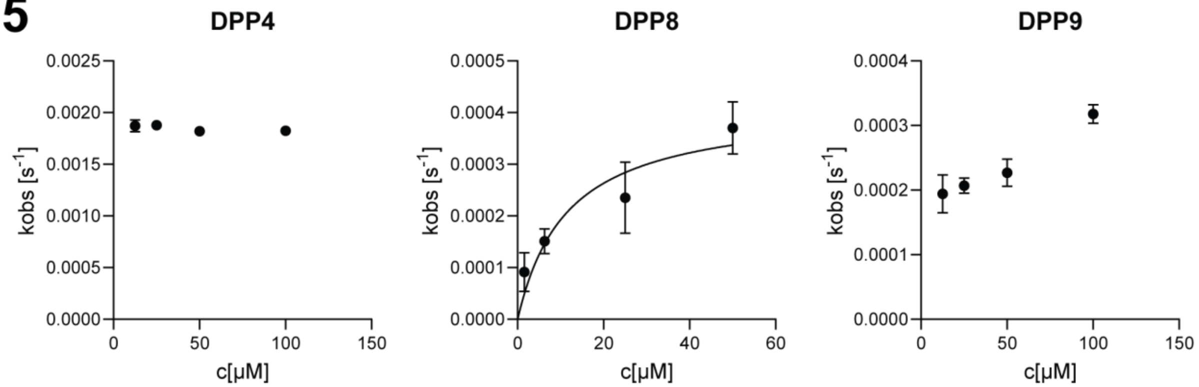
DPP4
DPP8
DPP9
kobs [s-1]
c[μM]

6

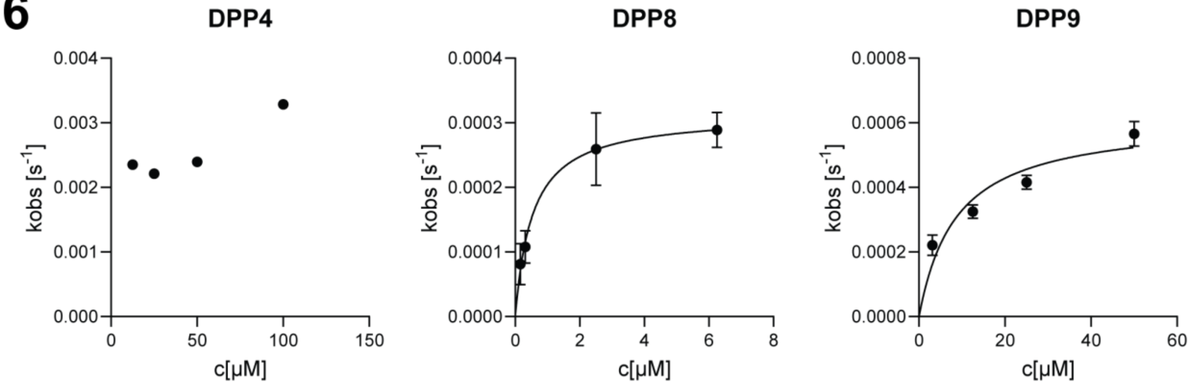
DPP4
DPP8
DPP9
kobs [s-1]
c[μM]

7

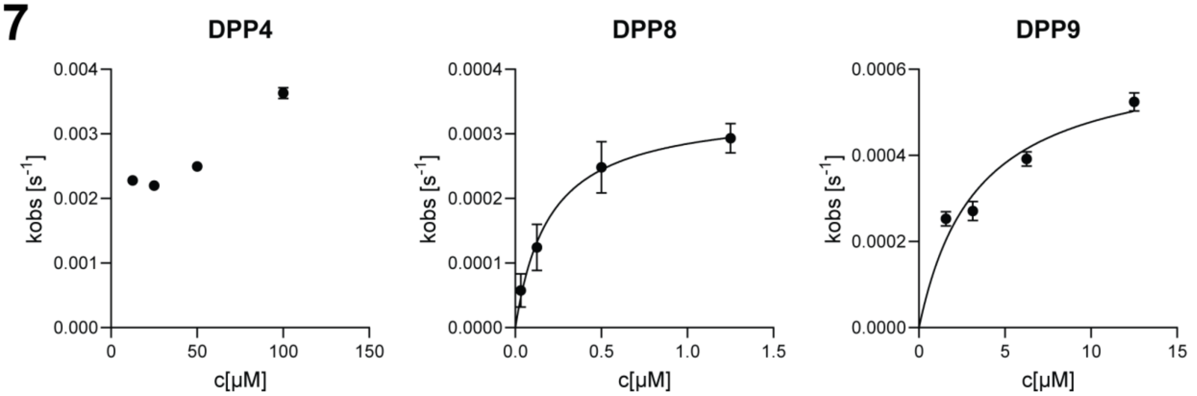
DPP4
DPP8
DPP9
kobs [s-1]
c[μM]

8

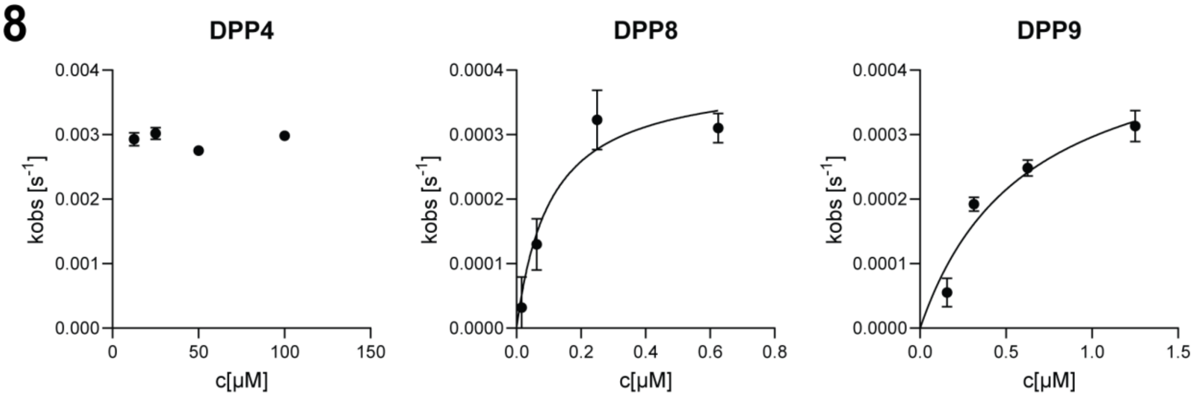
DPP4
DPP8
DPP9
kobs [s-1]
c[μM]

9

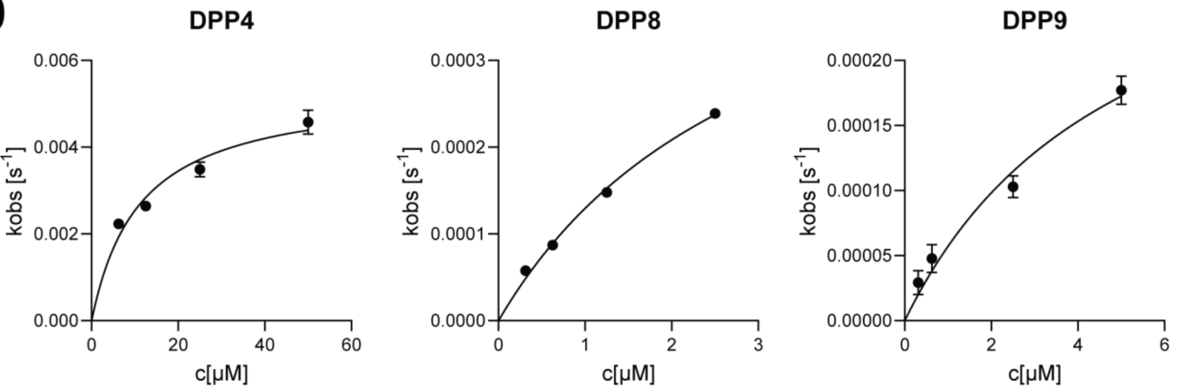
DPP4
DPP8
DPP9
kobs [s-1]
c[µM]

10

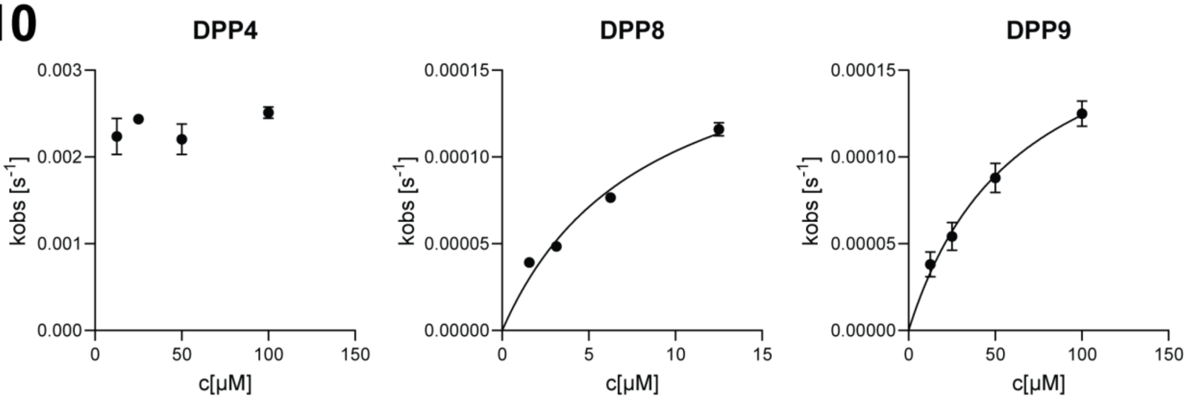
DPP4
DPP8
DPP9
kobs [s-1]
c[µM]

11

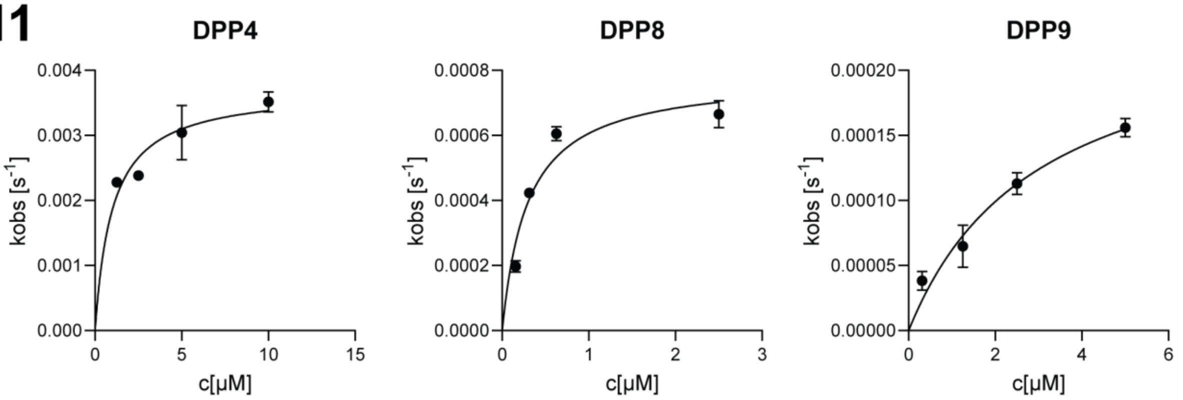
DPP4
DPP8
DPP9
kobs [s-1]
c[µM]

12

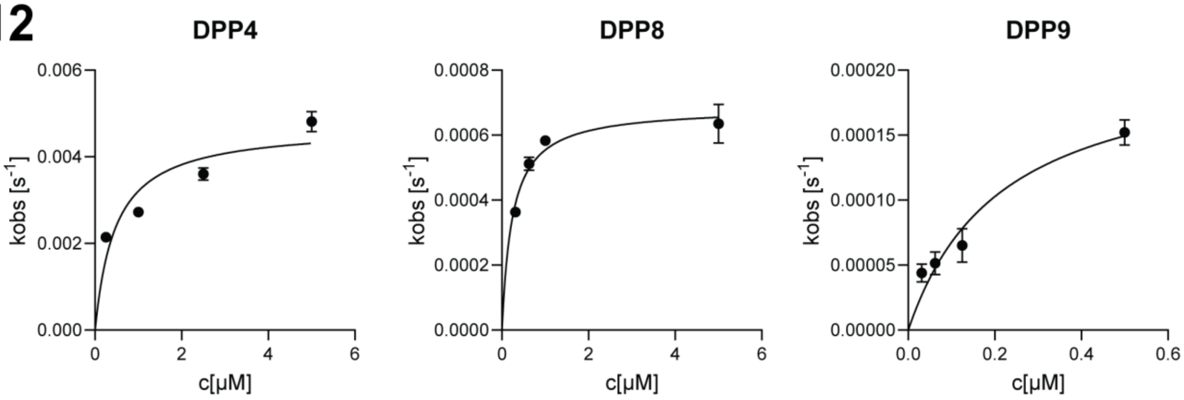
DPP4
DPP8
DPP9
kobs [s-1]
c[µM]

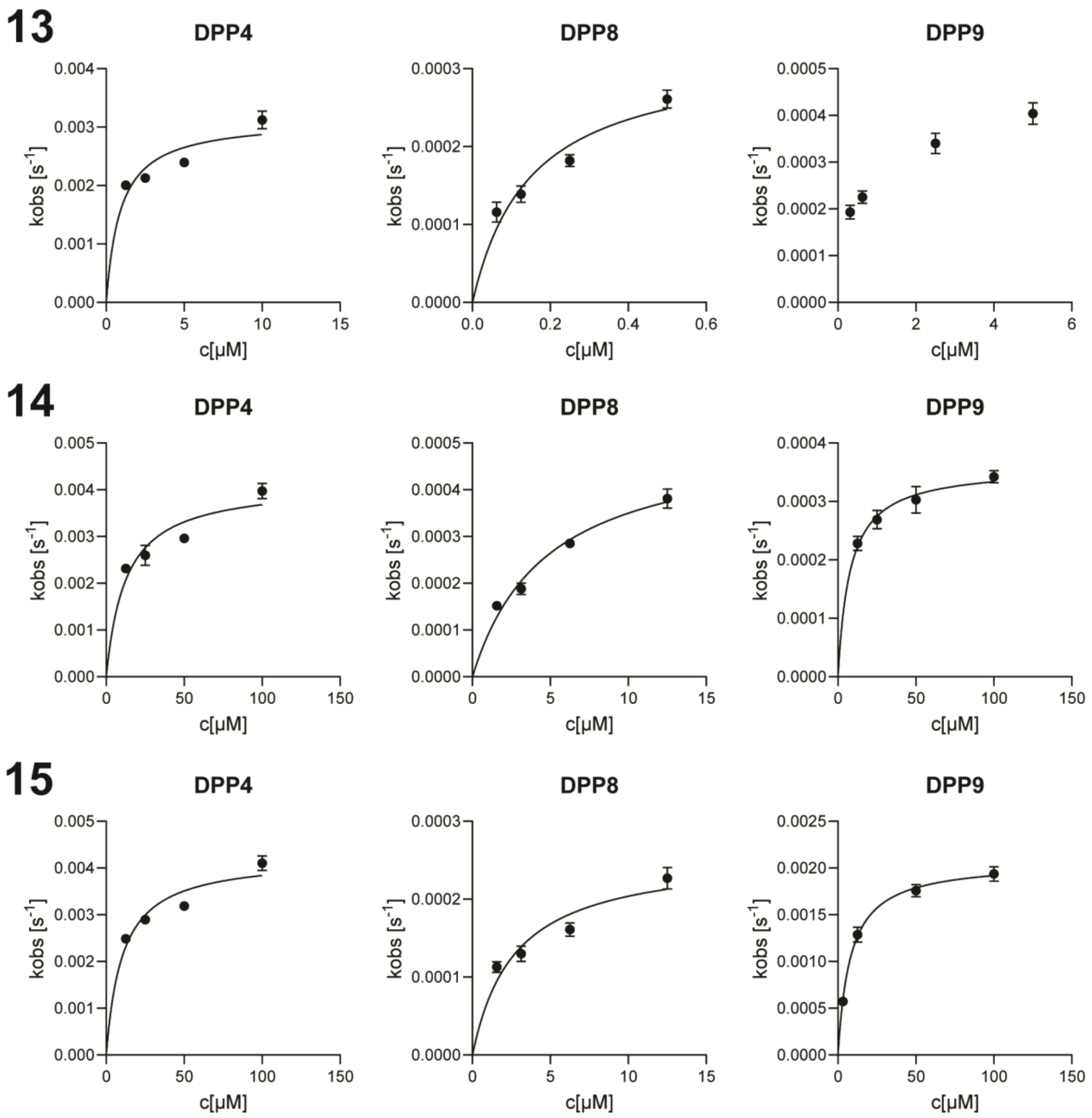

Supplementary Figure 8. **Kinetic analysis of DPP inhibition by *N*-phosphono-(*S*)-3-aminopiperidine-2-ones.** The pseudo-first order rate constant (k_{obs}) was calculated from an exponential regression of progress curves and plotted against the inhibitor concentration. K_I and k_{inact} were obtained via fitting to a hyperbolic equation. All measurements were performed in triplicate (*n* = 3 technical replicates), mean values are shown and the error bars indicate the SEM. Source data are provided as a Source Data file.

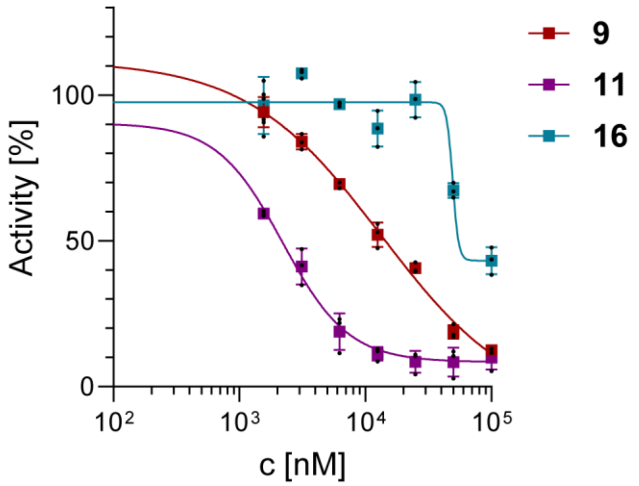

Supplementary Figure 9. **PREP inhibition curves of the *N*-phosphono-(*S*)-3-aminopiperidine-2-ones 9, 11 and 16.** All activity measurements were performed in triplicate (*n* = 3 technical replicates), mean values are shown and error bars indicate the SEM. Source data are provided as a Source Data file.

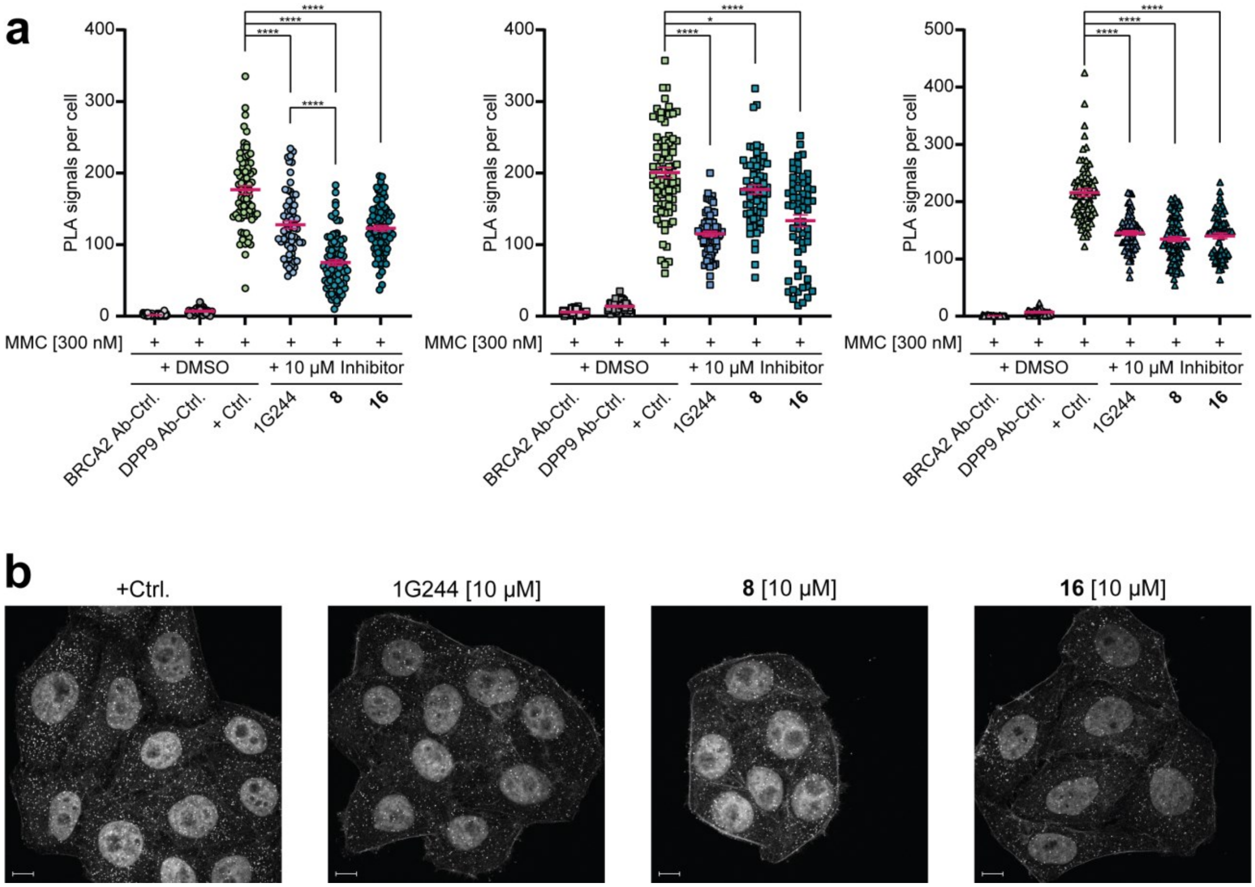

Supplementary Figure 10. **Independent biological replicates of PLA quantification, showing MMC-induced DPP9-BRCA2 PLA events in HeLa wild-type cells.** Cells were treated with 300 nM MMC for 24 h and received 10 µM of the indicated inhibitor 1 h prior to fixation. (**a**) Quantification of PLA events for each of the 3 biologically independent replicates, marked by varying shapes (replicate 1: *n* = 93 for BRCA2 Ab-Ctrl., *n* = 97 for DPP9 Ab-Ctrl., *n* = 78 for +Ctrl., *n* = 70 for 1G244, *n* = 87 for **8**, *n* = 83 for **16**; replicate 2: *n* = 72 for BRCA2 Ab-Ctrl., *n* = 76 for DPP9 Ab-Ctrl., *n* = 76 for +Ctrl., *n* = 69 for 1G244, *n* = 65 for **8**, *n* = 61 for **16**; replicate 3: *n* = 75 for BRCA2 Ab-Ctrl., *n* = 64 for DPP9 Ab-Ctrl., *n* = 68 for +Ctrl., *n* = 63 for 1G244, *n* = 79 for **8**, *n* = 61 for **16**; *n* values indicate total number of cells in each analysis group). Each dot represents the number of PLA events in a single cell. Data were analyzed by unpaired two-sided *t*-test comparisons (*p = 0.0148, ****p < 0.0001). Source data are provided as a Source Data file. (**b**) Representative PLA images showing close proximity between endogenous DPP9 and endogenous BRCA2 in HeLa wild-type cells (+Ctrl.). Inhibitor treatment reduced the PLA events (white dots) compared to the control. The images shown are representative of 3 independent biological replicates. Scale bar = 10 µm.

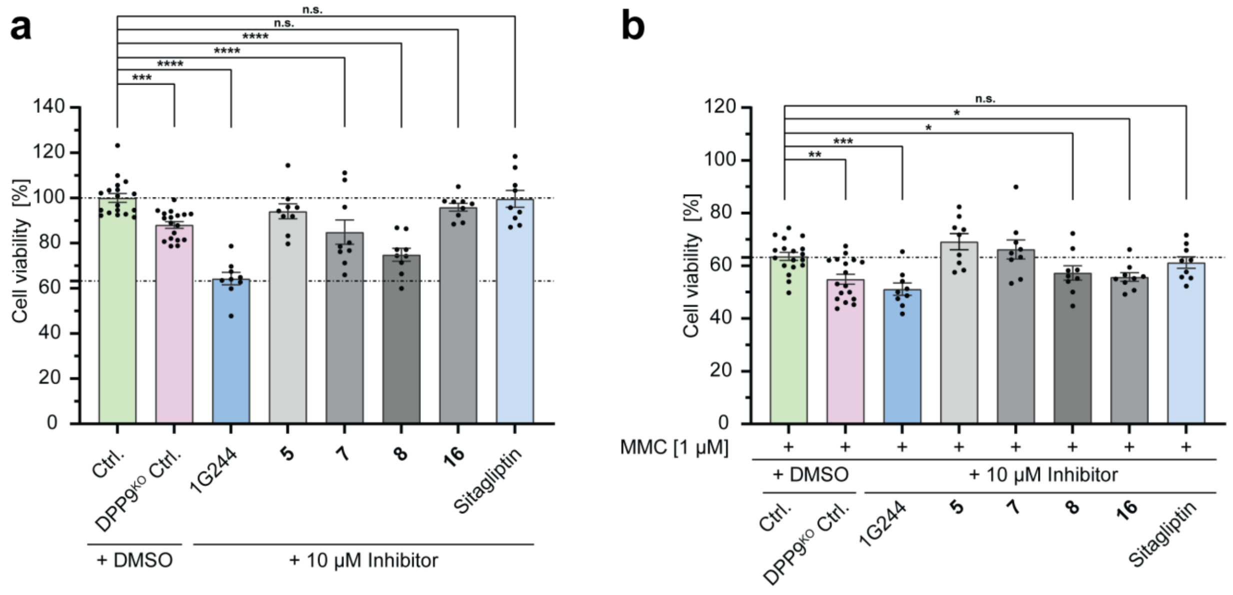

Supplementary Figure 11. **Effect of *N*-phosphono-(*S*)-3-aminopiperidinones on cell viability of HeLa cells.** (**a**) HeLa wild-type cells were incubated with 10 µM of the respective inhibitors for 72 hours. DPP9^{KO} cells, and 1G244 (10 µM) were analyzed as positive controls, whereas the DPP4-selective inhibitor Sitagliptin (10 µM) was used as a negative control. The graph shows the mean and error bars indicate the SEM of all individual measurements (*n* = 18 (HeLa wild-type and DPP9^{KO} control) or 9 (inhibitor treated samples) independent replicates). Data were analyzed by a Tukey two-way ANOVA, using a mixed effect analysis (n.s. = not significant, ***p = 0.002, ****p < 0.0001). (**b**) HeLa wild-type cells were treated with 1 µM MMC and 10 µM of the respective inhibitors. Cell viability was measured after 72 h. DPP9^{KO} cells and 1G244 (10 µM) were analyzed as positive controls, whereas Sitagliptin (10 µM) was used as a negative control. Dashed lines indicate the viability of the HeLa wild-type control. Data were normalized for each condition [+MMC +inhibitor] separately to the corresponding [-MMC + inhibitor] control. The graph shows the mean and error bars indicate the SEM of all individual measurements (*n* = 18 (HeLa wild-type controls), 17 (HeLa DPP9^{KO} control) or 9 (inhibitor treated samples) independent replicates). Data were analyzed by an uncorrected Fisher's LSD two-way ANOVA, using a mixed effect analysis (n.s. = not significant, ***p = 0.0002, **p = 0.002, *p = 0.0385 for Ctrl. vs. **8**, *p = 0.0127 for Ctrl. vs. **16**). Source data are provided as a Source Data file.

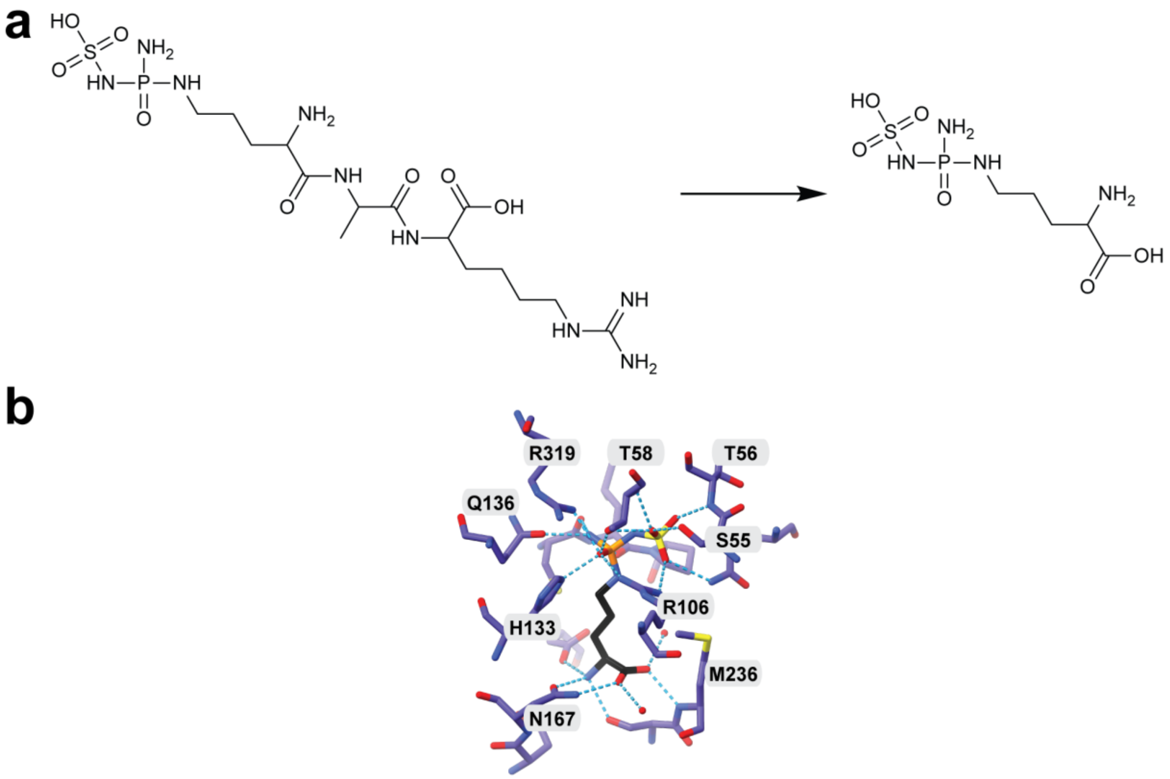

Supplementary Figure 12. **Phaseolotoxin and its cleavage product Octicidin are representatives of the sulpho-phosphotriamide natural product family.** (**a**) Chemical structure of the natural product Phaseolotoxin and its cleavage product Octicidin. (**b**) X-ray structure of ornithine transcarbamoylase complexed with *N* $^{\delta}$-(*N′*-sulfodiaminophosphinyl)-L-ornithine. Crystal structure from PDB: 1DUV.

Supplementary Tables

Supplementary Table 1. Rate constants for irreversible inhibition of DPPs by *N*-phosphonopiperidones compounds

		K_I [nM]	k_{inact} [s^{-1}]	kapp [M^{-1} s^{-1}]
**5**	DPP4	-	-	-
	DPP8	11,523 ± 5,611	0.00042 ± 0.00007	36
	DPP9	-	-	-
**6**	DPP4	-	-	-
	DPP8	532 ± 143	0.00031 ± 0.00002	589
	DPP9	8,661 ± 2,518	0.00061 ± 0.00005	71
**7**	DPP4	-	-	-
	DPP8	198 ± 51	0.00034 ± 0.00003	1,725
	DPP9	3,343 ± 739	0.00064 ± 0.00005	191
**8**	DPP4	-	-	-
	DPP8	105 ± 39	0.00039 ± 0.00004	3,757
	DPP9	613 ± 188	0.00048 ± 0.00007	780
**9**	DPP4	11,153 ± 1,993	0.00537 ± 0.00033	482
	DPP8	3,120 ± 363	0.00053 ± 0.00004	171
	DPP9	5,071 ± 1,811	0.00035 ± 0.00007	68
**10**	DPP4	-	-	-
	DPP8	8,251 ± 1,532	0.00019 ± 0.00002	23
	DPP9	-	-	-
**11**	DPP4	998 ± 248	0.00372 ± 0.00022	3,721
	DPP8	294 ± 62	0.00078 ± 0.00005	2,665
	DPP9	3,002 ± 873	0.00025 ± 0.00004	83
**12**	DPP4	470 ± 146	0.00472 ± 0.00035	10,026
	DPP8	237 ± 38	0.00069 ± 0.00003	2,891
	DPP9	217 ± 58	0.00021 ± 0.00003	986
**13**	DPP4	913 ± 240	0.00313 ± 0.00018	3,435
	DPP8	148 ± 33	0,00032 ± 0.00003	2,172
	DPP9	460 ± 71	0.00042 ± 0.00002	918
**14**	DPP4	12,567 ± 3,037	0.00414 ± 0.00026	330
	DPP8	4,760 ± 734	0.00052 ± 0.00003	108
	DPP9	7,736 ± 1,189	0.00036 ± 0.00001	46
**15**	DPP4	10,426 ± 2085	0.00424 ± 0.00020	407
	DPP8	2,703 ± 651	0.00026 ± 0.00002	96
	DPP9	7,954 ± 627	0.00207 ± 0.00004	260
**16**	DPP4	512,000 ± 269,800	0.01027 ± 0.00249	20
	DPP8	4 ± 2	0.00018 ± 0.00002	51,049
	DPP9	216 ± 90	0.00050 ± 0.00007	2,310

Supplementary Table 2. IC_{50} values [nM] for PREP inhibition by selected *N*-phosphonopiperidones (**9, 11, 16**)

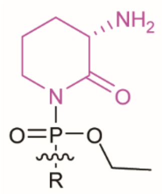	**R**	**PREP [nM]**
**9**	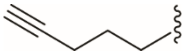	13,418 ± 4,040
**11**	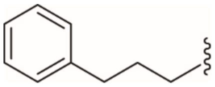	2,207 ± 1,577
**16**	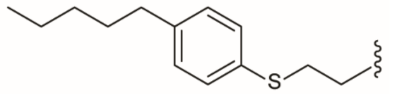	>50,000

Supplementary Table 3. Data collection and refinement statistics

	DPP9:**1**	DPP9 Ser730Ala:**1**	DPP4:**1**	DPP9:**16**
PDB code	9GON	9GOC	9GOH	9GOD
**Data Collection**				
Space group	P 1	P 1	P 2_1 2_1 2_1	P 1
Cell dimensions				
a; b; c [Å]	88.80; 106.68; 121.14	88.61; 106.53; 121.27	65.92; 68.21; 421.06	89.31; 106.75; 121.48
α; β; γ [°]	65.0; 70.8; 76.6	65.2; 70.7; 76.4	90.0; 90.0; 90.0	65.2; 71.0; 76.7
Resolution [Å]	1.89 (1.93-1.89)	1.89 (1.93-1.89)	2.38 (2.42-2.38)	2.49 (2.74-2.49)
Unique reflections	289674 (14469)	288843 (14452)	78066 (3786)	96524 (4826)
Multiplicity	2.2 (2.2)	2.2 (2.3)	8.2 (8.3)	3.7 (3.8)
Completeness [%]	96.3 (95.9)	96.2 (95.7)	99.9 (99.9)	Spherical: 71.8 (14.2)<br>Ellipsoidal: 92.2 (55.8)
R_{pim} [%]	3.9 (41.2)	3.6 (41.8)	2.8 (56.3)	7.9 (38.6)
R_{sym} [%]	4.7 (49.8)	4.3 (50.2)	7.6 (153.4)	13.0 (64.6)
R_{meas} [%]	6.2 (64.9)	5.7 (65.6)	8.1 (163.7)	15.3 (75.4)
$CC_{1/2}$ [%]	99.80 (75.40)	99.80 (78.30)	99.90 (91.30)	99.00 (67.60)
Mean(I)/sd	5.3 (1.3)	6.0 (1.3)	14.5 (1.3)	5.5 (1.4)
**Refinement**				
Resolution [Å]	90.21-1.89	83.24-1.89	210.53-2.38	106.55-2.49
Number of reflections (working /test)	287379 / 2295	286483 / 2336	76954 / 1109	95210 / 2036
R_{cryst}/R_{free} [%]	16.3/20.6	17.7/19.9	23.6/28.5	17.0/21.9
Total number of atoms:	30750	30013	12358	27151
Protein	28006	27432	11937	26297
Water	2373	2285	111	636
Ligand	32	64	16	76
B-factors				
Protein	38.4	41.9	73.3	51.2
Ligand	33.0	39.8	60.0	66.6
Water	44.4	45.5	59.7	33.0
Other atoms	57.2	53.5	116.7	53.2
Deviation from ideal geometry:				
Bond lengths [Å]	0.008	0.004	0.003	0.005
Bond angles [°]	1.47	1.32	1.34	1.38
Bonded B's [Å]	1.5	2.1	2.4	3.5

Supplementary Methods

Chemical Synthesis

General Information

Chemicals and Other Materials. All reactions were carried out under an atmosphere of argon. Used chemicals and (dry) solvents were commercially obtained from ABCR, Acros Organics, BLD Pharmatech, Fisher Chemical, Sigma Aldrich and VWR Chemicals. Flash chromatography was performed on silica gel with particle size of 60-200 µm and an average pore size of 60 Å. Thin layer chromatography (TLC) analysis was carried out on Merck $60F_{254}$ silica gel plates.

HPLC and Mass Spectrometry. Preparative HPLC reversed-phase chromatography was performed on a Shimadzu Prominence system with a LC-20AP pump equipped with a Phenomenex Luna® C18(2) 100Å column (5 µm, 100 x 21.2 mm) using, a gradient program with MeCN/MQ-water (0.1% TFA) at 20.0 mL min^{-1}. Analytical LC-MS (ESI) analysis was performed on a Thermo Scientific UltiMate 3000 HPLC system with a Thermo Scientific LTQ XL™ linear ion trap mass spectrometer, using a gradient program with eluent A = MQ-water with 0.1% formic acid and eluent B = acetonitrile with 0.1% formic acid, 0.5 min 10% B, then 10% to 100% B in 5.5 min, then 3.2 min 100% B, a flow rate of 1.0 mL min^{-1} on a Macherey-Nagel NUCLEODUR C18 Pyramid column (particle size 5 µm, 250 mm x 4.0 mm) at 25 °C. HRMS (ESI) analyses were performed on a Thermo Scientific Exactive Plus EMR mass spectrometer.

Nuclear Magnetic Resonance. NMR spectra of intermediate compounds were recorded with a Bruker 400 MHz Avance II spectrometer, equipped with a PATXI probe, with a 1H frequency of 399.99 MHz and 13C frequency of 100.59 MHz. NMR spectra of final compounds were recorded with a Bruker AV NEO 400 MHz spectrometer, equipped with a BBO Bruker Prodigy Cryoprobe, with a ^{1}H frequency of 400.13 MHz and ^{13}C frequency of 100.62 MHz. All 2D NMR spectra were acquired using NOAH (NMR by Ordered Acquisition using ^{1}H detection), with a 4166.7 Hz spectral width for ^{1}H and 19230.8 Hz spectral width for ^{13}C, using 512 x 256 points, with 2 scans per increment and a relaxation delay of 1.69 seconds. The ^{1}H-^{1}H COSY and multiplicity-edited ^{1}H-^{13}C HSQC spectra

were processed to 512 x 256 points. ^{1}H-^{13}C HMBC spectra were processed to 2048 x 2048 points. All experiments were performed at 298 K. Residual ^{1}H resonance from the deuterated solvents was used to reference the ^{1}H spectra with the methyl resonance of tetramethylsilane (δ = 0.00 ppm), the ^{13}C spectra were referenced through the solvent ^{13}C resonance.

Synthetic Procedures

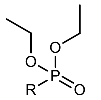

General Procedure A for the Preparation of Alkyl Diethoxyphosphoryl Derivatives (SI-7, SI-8, SI-9, SI-10, SI-11, SI-12). The corresponding bromide (<25 mmol, 1.0 equiv.) was dissolved in triethyl phosphite (1.5 equiv.) in a closed pressure tube and heated to 160 °C for 3-5 days, after which the residual volatiles were removed *in vacuo* at 100 °C. The resulting crude residue was purified by flash column chromatography (SiO_2, EtOAc/DCM) to provide the corresponding diethoxyphosphoryl derivative.

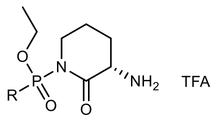

General Procedure B for the Preparation of (3*S*)-3-amino-1-[(*R*/*S*)-alkyl (ethoxy) phosphoryl]piperidin-2-one derivatives (5, 6, 7, 8, 9, 10, 11, 12, 13, 14). A solution of alkyl diethoxyphosphoryl derivative (0.2-0.3 mmol, 1.0 equiv.) was dissolved in dry DCM (1.5 mL), cooled to 0 °C, and subsequently treated with oxalyl chloride (200 µL). The resulting mixture was slowly warmed to room temperature, stirred for 18 h, and concentrated *in vacuo*. The resulting residue was dissolved in dry THF (1 mL) and cooled to -78 °C, hereafter called 'chlorophosphonate derivative solution'.

In parallel, compound **4** (0.2-0.3 mmol, 1.0 equiv.) was dissolved in dry THF (1.0 mL), cooled to -78 °C, treated with a 2.5 M solution of *n*-butyllithium in hexanes (1.0 equiv.) and stirred for 45 minutes at -78 °C. The resulting mixture was transferred and added dropwise with a syringe to the 'chlorophosphonate derivative solution'. The resulting mixture was stirred for 45-90 minutes at -78 °C, allowed to warm to room temperature and stirred for another 10-60 minutes at room temperature, after which the reaction was quenched by the addition of half-saturated aqueous NH_4Cl (6 mL) and DCM (15 mL). The organic layer was collected, washed with brine (5 mL), dried over Na_2SO_4, filtered, and concentrated *in vacuo*. The resulting crude residue was dissolved in dry DCM (2.5 mL),

after which morpholine (2.0 equiv.) and $Pd(PPh_3)_4$ (0.02 equiv.) were added. The resulting mixture was stirred for 1.5 h at room temperature, after which it was concentrated *in vacuo* to provide a crude residue. This was purified by preparative reversed-phase chromatography (C18, acetonitrile/water with 0.1% TFA) to obtain the corresponding (3*S*)-3-amino-1-[(*R*/*S*)-alkyl (ethoxy) phosphoryl]piperidin-2-one derivative.

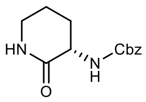

(*S*)-3-(Cbz-amino)-piperidin-2-one (SI-1). Compound **SI-1** was prepared from *L*-ornithine HCl according to the procedure of Abe *et al.*[1], providing **SI-1** (24.8 g, 84% with respect to *L*-ornithine) as a white solid. LC-MS (ESI) R_t = 6.5 min; *m/z* calcd. for $[C_{13}H_{17}N_2O_3]^+$ = 249.1, found 249.0. ^{1}H NMR (400 MHz, $CDCl_3$) δ 7.34 – 7.15 (m, 5H), 6.58 (s, 1H), 5.78 (s, 1H), 5.01 (s, 2H), 4.07 – 3.90 (m, 1H), 3.26 – 3.07 (m, 2H), 2.44 – 2.26 (m, 1H), 1.85 – 1.66 (m, 2H), 1.59 – 1.44 (m, 1H). ^{13}C NMR (101 MHz, $CDCl_3$) δ 171.7, 156.5, 136.5, 128.6, 128.2, 66.9, 51.7, 41.8, 27.7, 21.1. The spectroscopic data are in accordance with the literature[1].

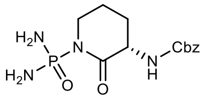

(*S*)-3-(Cbz-amino)-1-(diaminophosphoryl)-piperidin-2-one (SI-2). Compound **SI-2** was prepared from **SI-1** according to the TMSCl in toluene mediated lactam amide activation procedure of Abe *et al.*[2], providing **SI-2** (4.98 g, 32%) as a white solid. LC-MS (ESI) R_t = 5.9 min; *m/z* calcd. for $[C_{13}H_{19}N_4O_4P]^+$ = 327.1, found 327.0. ^{1}H NMR (400 MHz, DMSO-D_6) δ 7.48 – 7.24 (m, 6H), 5.04 (s, 2H), 4.21 (s, 2H), 4.16 (s, 2H), 4.13 – 4.04 (m, 1H), 3.66 – 3.53 (m, 1H), 3.53 – 3.42 (m, 1H), 2.11 – 1.93 (m, 1H), 1.84 – 1.71 (m, 2H), 1.70 – 1.55 (m, 1H). ^{13}C NMR (101 MHz, DMSO-D_6) δ 173.0, 156.1, 137.1, 128.4, 127.8, 65.4, 51.3, 42.7, 26.0, 21.3. The spectroscopic data are in accordance with the literature[2]**.**

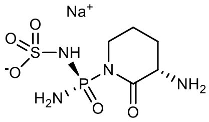

(Amino- (*R*)- ((*S*)-3- amino- 2-oxopiperidin- 1-yl) phosphoryl) sulfamate sodium salt (1). Compound **1** was prepared from **SI-2** according to the procedure of Abe *et al*[1]. In contrast to the described procedure, diastereomeric resolution was carried out by preparative reversed phase chromatography (C18, acetonitrile/water with 0.1% TFA). The fractions containing the desired isomer were combined, basified using concentrated aqueous $NaHCO_3$, and concentrated *in vacuo*. The resulting residue was desalted using column chromatography (Diaion HP-20SS, MeOH/water 3:7). The fractions containing the desired compound were concentrated *in vacuo*. The Cbz group was removed as described by Abe *et al.*[1], providing

1 (7.8 mg, 30% with respect to **SI-2**) as a white solid. LC-MS (ESI) R_t = 1.4 min; *m/z* calcd. for $[C_5H_{12}N_4O_5PS]^+$ = 271.0, found 271.3. ^{1}H NMR (400 MHz, D_2O) δ 3.84 – 3.74 (m, 1H), 3.70 – 3.64 (m, 1H), 3.62 – 3.50 (m, 2H), 2.26 – 2.13 (m, 1H), 2.05 – 1.77 (m, 2H), 1.71 – 1.55 (m, 1H). ^{31}P NMR (162 MHz, D_2O) δ 3.42.

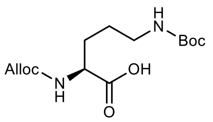

***N*-*α*-Alloc-*N*-*δ*-Boc-*L*-Ornithine (SI-18).** *N*-*δ*-Boc-*L*-Ornithine (5.00 g, 21.5 mmol, 1.0 equiv.) and sodium carbonate (2.73 g, 25.8 mmol, 1.2 equiv.) were dissolved in a mixture of 1,4-dioxane (100 mL) and water (100 mL). The resulting mixture was cooled to 0 °C, treated dropwise with allyl chloroformate (2.75 mL, 25.9 mmol, 1.2 equiv.), then slowly warmed to room temperature and stirred for 18 h. The mixture was washed with EtOAc (2× 100 mL), then the aqueous layer was collected, acidified to pH = 3 with 1 M aqueous $NaHSO_4$ and extracted with EtOAc (2× 200 mL). The organic layers were combined, washed with brine (100 mL), dried over Na_2SO_4, filtered, and concentrated *in vacuo* to provide compound **SI-18** as a clear colorless oil (7.26 g, yield n.d.), comprising low amounts of solvent, which was used in the next step without further purification. LC-MS (ESI) R_t = 6.6 min; *m/z* calcd. for $[C_{14}H_{24}N_2NaO_6]^+$ = 339.2, found 339.6. ^{1}H NMR (400 MHz, DMSO-D_6) δ 7.48 (d, *J* = 8.0 Hz, 1H), 6.76 (t, *J* = 5.7 Hz, 1H), 5.90 (ddt, *J* = 17.1, 10.6, 5.3 Hz, 1H), 5.29 (dd, *J* = 17.3, 1.8 Hz, 1H), 5.17 (dd, *J* = 10.5, 1.6 Hz, 1H), 4.47 (d, *J* = 5.3 Hz, 2H), 3.89 (td, *J* = 8.8, 4.6 Hz, 1H), 3.57 (d, *J* = 0.9 Hz, 3H), 2.89 (q, *J* = 6.5 Hz, 2H), 1.75 – 1.61 (m, 1H), 1.59 – 1.48 (m, 1H), 1.47 – 1.39 (m, 2H), 1.37 (s, 9H). ^{13}C NMR (101 MHz, DMSO-D_6) δ 173.9, 155.9, 155.6, 133.6, 117.0, 77.4, 66.4, 64.4, 53.6, 28.3, 28.2, 26.2. HRMS (ESI) *m*/*z* calcd. for $[C_{14}H_{25}N_2O_6]^+$ = 317.17071; found 317.17107 (|Δ| = 1.14 ppm).

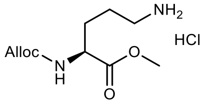

***N*-*α*-Alloc-*L*-Ornithine hydrochloride (SI-19).** Compound **SI-18** (7.23 g) was dissolved in 1 M hydrogen chloride in methanol (40 mL) and stirred for 18 h at room temperature. The resulting mixture was then concentrated *in vacuo* to provide compound **SI-19** as a clear colorless oil (5.77 g, yield n.d.), which was used in the next step without further purification. LC-MS (ESI) R_t = 4.1 min; *m/z* calcd. for $[C_{10}H_{19}N_2O_4]^+$ = 231.1, found 231.2. ^{1}H NMR (400 MHz, DMSO-D_6) δ 8.12 (br, 3H), 7.74 (d, *J* = 7.8 Hz, 1H), 5.90 (ddt, *J* = 16.1, 10.5, 5.3 Hz, 1H), 5.29 (d, *J* = 17.9 Hz, 1H), 5.18 (d, *J* = 10.4 Hz, 1H), 4.47 (d, *J* = 5.2 Hz, 2H), 4.01 (td, *J* = 8.3, 4.3 Hz, 1H), 3.63 (s, 3H), 2.73 (s, 2H), 1.83 – 1.68 (m, 1H), 1.69 – 1.56 (m, 3H). ^{13}C NMR (101 MHz, DMSO-D_6) δ

172.5, 155.9, 133.4, 117.2, 64.6, 53.4, 51.9, 38.2, 27.6, 23.7. HRMS (ESI) *m*/*z* calcd. for $[C_{20}H_{37}N_4O_8(=2M+H)]^+$ = 461.26059; found 461.25892 (|Δ| = 3.62 ppm).

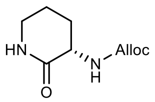

(*S*)-3-(Alloc-amino)-piperidin-2-one (4). Compound **SI-19** (5.74 g) was dissolved in a mixture of EtOAc (50 mL) and MeOH (2.5 mL), and subsequently treated with $NaHCO_3$ (2.71 g, 32.2 mmol, ≥ 1.5 equiv.). The resulting mixture was stirred for 3 days at room temperature, washed with 0.5 M hydrochloric acid (2× 30 mL) and brine (25 mL), dried over Na_2SO_4, and concentrated *in vacuo* to provide a white solid which was triturated with Et_2O to provide compound **4** (1.09 g, 26% over last three steps). LC-MS (ESI) R_t = 5.4 min; *m/z* calcd. for $[C_9H_{15}N_2O_3]^+$ = 199.1, found 199.1. ^{1}H NMR (400 MHz, DMSO-D_6) δ 7.51 (s, 1H), 7.29 (d, *J* = 8.6 Hz, 1H), 5.90 (ddt, *J* = 17.0, 10.5, 5.3 Hz, 1H), 5.29 (dd, *J* = 17.2, 1.7 Hz, 1H), 5.17 (dd, *J* = 10.6, 1.7 Hz, 1H), 4.46 (d, *J* = 5.4 Hz, 1H), 3.86 (ddd, *J* = 10.3, 8.5, 5.9 Hz, 1H), 3.16 – 3.02 (m, 3H), 2.00 – 1.88 (m, 1H), 1.85 – 1.56 (m, 3H). ^{13}C NMR (101 MHz, DMSO-D_6) δ 169.8, 155.9, 133.7, 116.9, 64.3, 50.6, 41.0, 27.8, 21.2. HRMS (ESI) *m/z* calcd. for $[C_{18}H_{29}N_4O_6(=2M+H)]^+$ = 397.20816; found 397.20824 (|Δ| = 0.20 ppm).

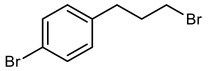

1-Bromo-4-(3-bromopropyl)benzene (SI-4). 1-Bromo-3-phenylpropane (**SI-3**, 5.00 g, 25.1 mmol, 1.0 equiv.) was dissolved in hexafluoroisopropanol (100 mL) and treated with *N*-bromosuccinimide (4.91 g, 27.6 mmol, 1.1 equiv). The resulting mixture was stirred for 3 days at room temperature and subsequently concentrated *in vacuo*. The obtained residue was redissolved in DCM (100 mL), washed with water (100 mL), saturated aqueous $NaHCO_3$ (100 mL) and brine (100 mL), dried over Na_2SO_4, filtered, and concentrated *in vacuo* to obtain a crude residue. This was purified by flash column chromatography (100 g SiO_2, cyclohexane → cyclohexane/EtOAc 19:1) to provide compound **SI-4** as a clear colorless oil (4.17 g, 60%). TLC R_f = 0.5 (SiO_2, DCM/EtOAc 19:1). HPLC R_t = 9.2 min. ^{1}H NMR (400 MHz, $CDCl_3$) δ 7.42 (d, *J* = 8.4 Hz, 2H), 7.08 (d, *J* = 8.4 Hz, 2H), 3.38 (t, *J* = 6.5 Hz, 3H), 2.74 (t, *J* = 7.4 Hz, 2H), 2.18 – 2.07 (m, 3H). ^{13}C NMR (101 MHz, $CDCl_3$) δ 139.6, 131.7, 130.4, 120.1, 34.0, 33.5, 32.9.

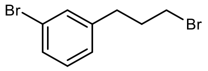

1-Bromo-3-(3-bromopropyl)benzene (SI-6). A solution of 3-(3-bromophenyl)propanoic acid (**SI-5**, 5.00 g, 21.8 mmol, 1.0 equiv.) in THF (200 mL) was cooled to 0 °C, and treated with *N*-methyl morpholine (4.85 mL, 43.6 mmol,

2.0 equiv.), followed by the addition of ethyl chloroformate (4.36 mL, 45.8 mmol, 2.1 equiv.). The mixture was stirred for 10 minutes at 0 °C, after which the white precipitate was removed by filtration. A solution of $NaBH_4$ (2.63 g, 69.5 mmol, 3.2 equiv.) in water (25 mL) was added to the filtrate and stirred for 1 h at 0 °C. Water (300 mL) was added to the mixture, and the aqueous layer was extracted with EtOAc (3× 100 mL). The organic layers were combined and washed with 0.5 M hydrochloric acid (3× 50 mL), brine (50 mL), dried over Na_2SO_4, filtered, and concentrated *in vacuo* to provide the intermediate alcohol.

The intermediate alcohol was dissolved in dry DCM (45 mL), and treated dropwise with a solution of PBr (1.02 mL, 10.7 mmol, 0.49 equiv.) in dry DCM (15 mL). The resulting mixture was stirred for 1 h at room temperature, and quenched by the addition of water (25 mL). The organic phase was collected and washed with water (2× 25 mL) and brine (25 mL), dried over Na_2SO_4, filtered, and concentrated *in vacuo*. The obtained crude residue was purified by flash column chromatography (100 g SiO_2, cyclohexane → cyclohexane/EtOAc 19:1) to provide compound **SI-6** as a clear colorless oil (2.02 g, 33%). TLC R_f = 0.5 (SiO_2, DCM/EtOAc 19:1). HPLC R_t = 9.2 min. ^{1}H NMR (400 MHz, $CDCl_3$) δ 7.29 – 7.25 (m, 2H), 7.12 – 7.03 (m, 2H), 3.31 (t, J = 6.5 Hz, 2H), 2.68 (t, J = 7.4 Hz, 2H), 2.16 – 2.00 (m, 2H). ^{13}C NMR (101 MHz, $CDCl_3$) δ 142.9, 131.6, 130.1, 129.4, 127.3, 122.6, 33.9, 33.6, 32.7.

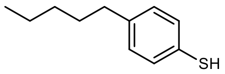

4-Pentylthiophenol (SI-16). 4-Pentylbenzenesulfonyl chloride (**SI-15**, 2.28 g, 10.0 mmol, 1.0 equiv.) was dissolved in toluene (60 mL), cooled to 0 °C, and treated portion wise with triphenylphosphine (7.87 g, 30.0 mmol, 3.0 equiv.). The resulting mixture was heated to reflux for 10 minutes, and subsequently cooled to 50 °C, after which water (10 mL) was added to the mixture. The mixture was allowed to cool to room temperature and diluted with toluene (200 mL), the aqueous layer was separated and discarded, the organic layer was then extracted with 10% aqueous NaOH (2× 150 mL). The combined aqueous layers were then washed with toluene (2× 150 mL), acidified to pH 4 with concentrated hydrochloric acid, and extracted with DCM (3× 200 mL). The combined DCM layers were washed with brine (100 mL), dried over Na_2SO_4, filtered, and concentrated *in vacuo*, to provide compound **SI-16** as a clear colorless oil (356 mg, 20%), which was used in the next step without further purification. ^{1}H NMR (400 MHz, $CDCl_3$) δ

7.27 (d, J = 8.1 Hz, 2H), 7.12 (d, J = 8.1 Hz, 2H), 3.45 (s, 1H), 2.68 – 2.55 (m, 2H), 1.70 – 1.59 (m, 2H), 1.46 – 1.29 (m, 4H), 0.95 (t, J = 6.8 Hz, 3H). ^{13}C NMR (101 MHz, $CDCl_3$) δ 140.9, 130.0, 129.3, 126.9, 35.5, 31.6, 31.2, 22.7, 14.2.

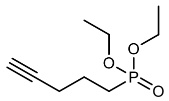

Diethyl (pent-4-ynyl)phosphonate (SI-7). Pent-4-yn-1-ol (2.00 g, 23.8 mmol, 1.0 equiv.) was dissolved in diethyl ether (30 mL), cooled to 0 °C, treated with phosphorus tribromide (1.12 mL, 11.8 mmol, 0.5 equiv.) and stirred for 2 h at 0 °C. The resulting mixture was quenched by the addition of saturated aqueous NH_4Cl (20 mL) on ice, neutralized using 5% aqueous $NaHCO_3$ and extracted with diethyl ether (3× 50 mL). The organic layers were combined, dried over Na_2SO_4, filtered and carefully concentrated *in vacuo* to provide the volatile intermediate bromide, which was used directly in general procedure **A** to provide compound **SI-7** as a clear colorless oil (742 mg, 15%). TLC R_f = 0.2 (SiO_2, DCM/EtOAc 9:1). LC-MS (ESI) R_t = 6.6 min; *m/z* calcd. for $[C_9H_{18}O_3P]^+$ = 205.1, found 205.1. ^{1}H NMR (400 MHz, $CDCl_3$) δ 4.23 – 3.95 (m, 4H), 2.35 – 2.23 (m, 2H), 1.99 – 1.92 (m, 1H), 1.92 – 1.79 (m, 3H), 1.79 – 1.64 (m, 1H), 1.38 – 1.04 (m, 6H). ^{13}C NMR (101 MHz, $CDCl_3$) δ 83.0, 69.4, 61.6 (d, J = 6.6 Hz), 24.6 (d, J = 142.2 Hz), 21.6 (d, J = 4.5 Hz), 19.2 (d, J = 17.8 Hz), 16.5 (d, J = 5.9 Hz). HRMS (ESI) *m/z* calcd. for $[C_{18}H_{35}O_6P_2(=2M+H)]^+$ = 409.19034; found 409.19036 (|Δ| = 0.05 ppm).

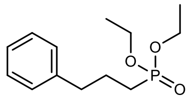

Diethyl (3-phenylpropyl)phosphonate (SI-8). Prepared using 1-bromo-3-phenyl-propane through general procedure **A**, provided as clear colorless oil (2.68 g, 44%). TLC R_f = 0.2 (SiO_2, DCM/EtOAc 9:1). LC-MS (ESI) R_t = 7.6 min; *m/z* calcd. for $[C_{13}H_{22}O_3P]^+$ = 257.1, found 257.3. ^{1}H NMR (400 MHz, $CDCl_3$) δ 7.37 (t, J = 7.0 Hz, 2H), 7.31 – 7.22 (m, 3H), 4.26 – 4.05 (m, 4H), 2.79 (t, J = 7.5 Hz, 2H), 2.13 – 1.95 (m, 2H), 1.89 – 1.76 (m, 2H), 1.39 (t, J = 7.1 Hz, 6H). ^{13}C NMR (101 MHz, $CDCl_3$) δ 141.2, 128.6, 128.5, 126.2, 61.6 (d, J = 6.8 Hz), 36.6 (d, J = 17.1 Hz), 25.2 (d, J = 141.1 Hz), 24.3 (d, J = 4.8 Hz), 16.6 (d, J = 6.2 Hz). HRMS (ESI) *m/z* calcd. for $[C_{13}H_{22}O_3P]^+$ = 257.13011; found 257.13025 (|Δ| = 0.54 ppm). The spectroscopic data are in accordance with the literature.[3]

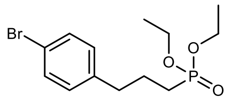

Diethyl (3-(4-bromophenyl)propyl)phosphonate (SI-9). Prepared using compound **SI-4** through general procedure **A**, provided as clear colorless oil (1.18 g, 49%). TLC R_f = 0.2 (SiO_2, DCM/EtOAc 4:1). LC-MS (ESI) R_t = 8.2 min; *m/z* calcd. for $[C_{13}H_{21}BrO_3P]^+$ = 335.0, found 335.8. ^{1}H NMR (400 MHz, $CDCl_3$) δ

7.39 (d, J = 8.3 Hz, 2H), 7.04 (d, J = 8.2 Hz, 2H), 4.17 – 3.96 (m, 4H), 2.65 (t, J = 7.5 Hz, 2H), 2.01 – 1.84 (m, 2H), 1.79 – 1.63 (m, 2H), 1.30 (t, J = 7.1 Hz, 6H). ^{13}C NMR (101 MHz, $CDCl_3$) δ 140.0, 131.5, 130.3, 119.9, 61.5 (d, J = 6.4 Hz), 35.8 (d, J = 16.7 Hz), 25.0 (d, J = 141.6 Hz), 24.0 (d, J = 4.7 Hz), 16.5 (d, J = 5.9 Hz). HRMS (ESI) *m*/*z* calcd. for $[C_{13}H_{21}BrO_3P]^+$ = 335.04062; found 335.04074 (|Δ| = 0.36 ppm).

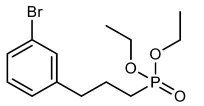

Diethyl (3-(3-bromophenyl)propyl)phosphonate (SI-10). Prepared using compound **SI-6** through general procedure **A**, provided as clear colorless oil (1.55 g, 64%). TLC R_f = 0.2 (SiO_2, DCM/EtOAc 4:1). LC-MS (ESI) R_t = 8.2 min; *m/z* calcd. for $[C_{13}H_{21}BrO_3P]^+$ = 335.0, found 335.9. ^{1}H NMR (400 MHz, $CDCl_3$) δ 7.28 – 7.24 (m, 2H), 7.11 – 7.00 (m, 2H), 4.10 – 3.94 (m, 4H), 2.60 (t, J = 7.5 Hz, 2H), 1.93 – 1.77 (m, 2H), 1.71 – 1.59 (m, 2H), 1.25 (t, J = 7.1 Hz, 6H). ^{13}C NMR (101 MHz, $CDCl_3$) δ 143.5, 131.7, 130.1, 129.4, 127.3, 122.6, 61.7 (d, J = 6.5 Hz), 36.1 (d, J = 17.0 Hz), 25.2 (d, J = 141.4 Hz), 24.1 (d, J = 4.7 Hz), 16.6 (d, J = 6.1 Hz). HRMS (ESI) *m*/*z* calcd. for $[C_{13}H_{21}BrO_3P]^+$ = 335.04062; found 335.04074 (|Δ| = 0.36 ppm).

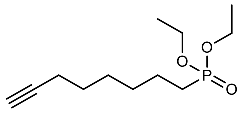

Diethyl (oct-7-yn-1-yl)phosphonate (SI-11). Prepared using 8-bromo-1-octyne through general procedure **A**, provided as clear colorless oil (174 mg, 54%). TLC R_f = 0.3 (SiO_2, cyclohexane/EtOAc 1:1). HPLC R_t = 9.2 min. ^{1}H NMR (400 MHz, $CDCl_3$) δ 4.14 – 3.98 (m, 4H), 2.16 (td, J = 6.9, 2.6 Hz, 2H), 1.92 (t, J = 2.6 Hz, 1H), 1.77 – 1.64 (m, 2H), 1.66 – 1.55 (m, 2H), 1.50 (q, J = 6.9 Hz, 2H), 1.45 – 1.34 (m, 4H), 1.30 (t, J = 7.0 Hz, 6H). ^{13}C NMR (101 MHz, $CDCl_3$) δ 84.6, 68.4, 61.6 (d, J = 6.6 Hz), 30.2, 30.1, 28.3, 25.7 (d, J = 140.6 Hz), 22.4 (d, J = 5.3 Hz), 18.4, 16.6 (d, J = 6.0 Hz). HRMS (ESI) *m*/*z* calcd. for $[C_{24}H_{47}O_6P_2(=2M+H)]^+$ = 493.28424; found 493.28479 (|Δ| = 1.11 ppm).

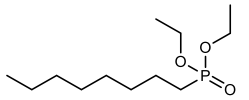

Diethyl octylphosphonate (SI-12). Prepared using 1-bromoctane through general procedure **A**, provided as clear colorless oil (857 mg, 66%). TLC R_f = 0.3 (SiO_2, DCM/EtOAc 4:1). LC-MS (ESI) R_t = 9.0 min; *m/z* calcd. for $[C_{12}H_{28}O_3P]^+$ = 251.2, found 251.4. ^{1}H NMR (400 MHz, $CDCl_3$) δ 4.17 – 3.89 (m, 4H), 1.74 – 1.62 (m, 2H), 1.62 – 1.49 (m, 2H), 1.39 – 1.30 (m, 2H), 1.29 (t, J = 7.1 Hz, 6H), 1.26 – 1.18 (m, 8H), 0.85 (t, J = 6.8 Hz, 3H). ^{13}C NMR (101 MHz, $CDCl_3$) δ 61.5 (d, J = 6.6 Hz), 31.9, 30.7 (d, J = 17.0 Hz), 29.1, 25.8 (d, J = 140.3 Hz), 22.7, 22.5 (d, J = 5.3 Hz), 16.6 (d, J = 6.0 Hz), 14.2. HRMS (ESI) *m/z* calcd. for $[C_{12}H_{28}O_3P]^+$ = 251.17706;

found 251.17722 (|Δ| = 0.64 ppm). The spectroscopic data are in accordance with the literature [4].

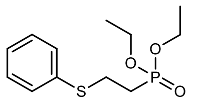

Diethyl (2-(phenylthio)ethyl)phosphonate (SI-13). Diethyl (2-bromoethyl)phosphonate (490 mg, 2.00 mmol, 1.0 equiv.) was dissolved in DMF (5 mL) and treated with thiophenol (**SI-17**, 224 µL, 2.20 mmol, 1.1 equiv.) and K_2CO_3 (828 mg, 5.99 mmol, 3.0 equiv.). The resulting mixture was stirred for 22 h at room temperature, and extracted with EtOAc (50 mL). The organic layer was collected, washed with 0.2 M hydrochloric acid (3× 50 mL), and brine (50 mL), dried over Na_2SO_4, filtered, and concentrated *in vacuo* to obtain a crude residue. This was purified by flash column chromatography (20 g SiO_2, cyclohexane/EtOAc 1:1 → 0:1) to provide compound **SI-13** as a clear colorless oil (350 mg, 64%). TLC R_f = 0.4 (SiO_2, cyclohexane/EtOAc 1:4). LC-MS (ESI) R_t = 7.7 min; *m/z* calcd. for $[C_{12}H_{20}O_3PS]^+$ = 275.1, found 275.3. ^{1}H NMR (400 MHz, $CDCl_3$) δ 7.28 – 7.24 (m, 2H), 7.21 (dd, *J* = 8.5, 6.7 Hz, 2H), 7.15 – 7.10 (m, 1H), 4.09 – 3.93 (m, 4H), 3.10 – 2.94 (m, 2H), 2.04 – 1.89 (m, 2H), 1.23 (t, *J* = 7.1 Hz, 6H). ^{13}C NMR (101 MHz, $CDCl_3$) δ 135.1, 130.1, 129.2, 126.8, 61.9 (d, J = 6.7 Hz), 27.3 (d, J = 2.6 Hz), 26.6 (d, J = 130.5 Hz), 16.6 (d, J = 6.0 Hz). HRMS (ESI) *m/z* calcd. for $[C_{12}H_{20}O_3PS]^+$ = 275.08653; found 275.08660 (|Δ| = 0.25 ppm).

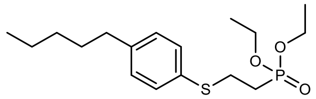

Diethyl (2-(4-pentylphenylthio)ethyl)phosphonate (SI-14). Diethyl (2-bromoethyl)phosphonate (337 mg, 1.38 mmol, 1.0 equiv.) was dissolved in DMF (5 mL) and treated with thiol **SI-16** (270 mg, 1.50 mmol, 1.1 equiv.) and K_2CO_3 (563 mg, 4.07 mmol, 3.0 equiv.). The resulting mixture was stirred for 22 h at room temperature, and extracted with EtOAc (50 mL). The organic layer was collected, washed with 0.2 M hydrochloric acid (3× 50 mL), and brine (50 mL), dried over Na_2SO_4, filtered, and concentrated *in vacuo* to obtain a crude residue. This was purified by flash column chromatography (20 g SiO_2, cyclohexane/EtOAc 4:1 → 1:1) to provide compound **SI-14** as a clear colorless oil (432 mg, 91%). TLC R_f = 0.3 (SiO_2, cyclohexane/EtOAc 4:1). LC-MS (ESI) R_t = 9.6 min; *m/z* calcd. for $[C_{17}H_{30}O_3PS]^+$ = 345.2, found 345.3. ^{1}H NMR (400 MHz, $CDCl_3$) δ 7.26 (dd, *J* = 8.4, 2.0 Hz, 2H), 7.10 (d, *J* = 8.2 Hz, 2H), 4.12 – 4.00 (m, 4H), 3.10 – 3.00 (m, 2H), 2.55 (dd, *J* = 8.7, 6.8 Hz, 2H), 2.08 – 1.96 (m, 2H), 1.64 – 1.52 (m, 2H), 1.29 (t, *J* = 7.1 Hz, 10H), 0.87 (t, *J* = 6.9 Hz, 3H). ^{13}C NMR (101 MHz, $CDCl_3$) δ 142.1, 131.4, 130.9, 129.3, 61.9 (d, *J* = 6.6 Hz), 35.6, 31.6,

31.2, 27.8 (d, J = 3.6 Hz), 26.7 (d, J = 135.6 Hz), 22.6, 16.6 (d, J = 5.9 Hz), 14.1. HRMS (ESI) m/z calcd. for $[C_{17}H_{30}O_3PS]^+$ = 345.16478; found 345.16479 (|Δ| = 0.03 ppm).

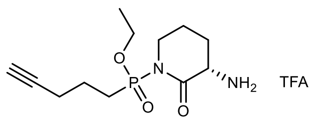

(3*S*)-3-amino-1-[(*R*/*S*)-ethoxy(pent-4-ynyl)phosphoryl] piperidin-2-one trifluoroacetate (9). Prepared using compound **SI-7** through general procedure **B**, provided as clear colorless oil (14 mg, 7%, mixture of two P isomers). LC-MS (ESI) R_t = 4.5 min; m/z calcd. for $[C_{12}H_{22}N_2O_3P]^+$ = 273.1, found 273.2. ^{1}H NMR (400 MHz, DMSO-D$_6$) δ 8.38 (br, 3H), 4.26 – 4.11 (m, 1H), 4.08 – 3.95 (m, 1H), 3.93 – 3.83 (m, 1H), 3.63 – 3.42 (m, 2H), 2.87 – 2.76 (m, 1H), 2.33 – 2.17 (m, 3H), 2.20 – 1.96 (m, 2H), 1.96 – 1.77 (m, 2H), 1.77 – 1.57 (m, 3H), 1.25 – 0.95 (m, 3H). ^{13}C NMR (101 MHz, DMSO-D$_6$) δ 171.4, 171.3, 158.1 (q, J = 32.3 Hz), 116.8 (q, J = 298.0 Hz), 83.4, 83.4, 72.2, 71.8, 63.4 – 63.1 (m), 61.0 – 60.6 (m), 49.6, 49.6, 42.9 – 42.8 (m), 42.5 – 42.4 (m), 29.0 – 28.6 (m), 26.3 (d, J = 50.4 Hz), 25.0 (d, J = 51.2 Hz), 23.5, 23.4, 20.7 (d, J = 3.7 Hz), 20.2, 19.9 (m), 18.7 – 18.5 (m), 18.4 – 18.3 (m), 16.1 (dd, J = 6.6, 3.3 Hz), 14.20, 14.18, 5.6 – 5.4 (m). HRMS (ESI) m/z calcd. for $[C_{12}H_{22}N_2O_3P]^+$ = 273.13626; found 273.13655 (|Δ| = 1.06 ppm).

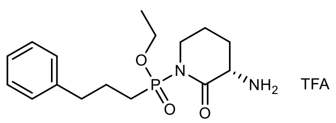

(3*S*)-3-amino-1-[(*R*/*S*)-ethoxy (3-phenylpropyl)phosphoryl] piperidin-2-one trifluoroacetate (11). Prepared using compound **SI-8** through general procedure **B**, provided as clear colorless oil (19 mg, 25%, mixture of two P isomers). LC-MS (ESI) R_t = 5.1 min; m/z calcd. for $[C_{16}H_{26}N_2O_3P]^+$ = 325.2, found 325.4. ^{1}H NMR (400 MHz, DMSO-D$_6$) δ 8.34 (br, 3H), 7.34 – 7.25 (m, 2H), 7.23 – 7.16 (m, 3H), 4.24 – 4.11 (m, 1H), 4.09 – 3.77 (m, 2H), 3.77 – 3.61 (m, 1H), 3.61 – 3.45 (m, 1H), 2.70 – 2.59 (m, 2H), 2.28 – 2.17 (m, 1H), 2.14 – 1.96 (m, 2H), 1.95 – 1.72 (m, 4H), 1.72 – 1.61 (m, 1H), 1.24 – 1.16 (m, 3H). ^{13}C NMR (101 MHz, DMSO-D$_6$) δ 171.4, 171.3, 158.0 (q, J = 32.0 Hz), 141.0, 128.4, 126.0, 116.9 (q, J = 298.2 Hz), 60.9 – 60.4 (m), 49.7 – 49.3 (m), 42.7, 42.3, 35.7 (d, J = 3.3 Hz), 35.4 (d, J = 3.4 Hz), 26.6 (d, J = 45.4 Hz), 25.4 (d, J = 46.0 Hz), 23.5, 23.3, 23.2 – 23.0 (m), 20.2 (d, J = 3.6 Hz), 20.0 (d, J = 2.3 Hz), 16.0 (d, J = 4.0 Hz). HRMS (ESI) m/z calcd. for $[C_{16}H_{26}N_2O_3P]^+$ = 325.16756; found 325.16807 (|Δ| = 1.57 ppm).

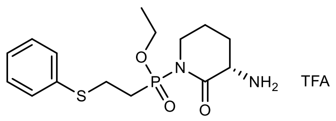

(3*S*)-3-amino-1-[(*R*/*S*)-ethoxy(2-(phenylthio)ethyl) phosphoryl]piperidin-2-one trifluoroacetate (14). Prepared using compound **SI-13** through general procedure **B**, provided

as a white solid (22 mg, 21%, mixture of two P isomers). LC-MS (ESI) R_t = 5.1 min; *m/z* calcd. for $[C_{15}H_{24}N_2O_3PS]^+$ = 343.1, found 343.8. ^{1}H NMR (400 MHz, DMSO-D$_6$) δ 8.38 (br, 3H), 7.38 – 7.32 (m, 4H), 7.27 – 7.21 (m, 1H), 4.15 (ddd, *J* = 24.1, 12.2, 7.8 Hz, 1H), 4.07 – 3.97 (m, 1H), 3.97 – 3.84 (m, 1H), 3.73 – 3.50 (m, 2H), 3.20 – 3.01 (m, 2H), 2.46 – 2.28 (m, 2H), 2.28 – 2.16 (m, 1H), 1.96 – 1.78 (m, 2H), 1.73 (m, 1H), 1.21 (q, *J* = 7.1 Hz, 3H). ^{13}C NMR (101 MHz, DMSO-D$_6$) δ 171.5, 158.5 – 157.4 (m), 134.9, 134.9, 129.3, 128.7, 128.6, 126.29, 126.26, 119.3 – 114.5 (m), 61.3 – 60.9 (m), 49.8 – 49.5 (m), 43.1, 42.7, 27.7 (d, *J* = 72.2 Hz), 26.5 (d, *J* = 73.1 Hz), 25.1 – 24.9 (m), 23.6, 23.4, 20.2 (d, *J* = 3.4 Hz), 20.0 (d, *J* = 2.3 Hz), 16.1 (d, *J* = 3.1 Hz), 16.0 (d, *J* = 3.3 Hz). HRMS (ESI) *m*/*z* calcd. for $[C_{15}H_{24}N_2O_3PS]^+$ = 343.12398; found 343.12441 (|Δ| = 1.25 ppm).

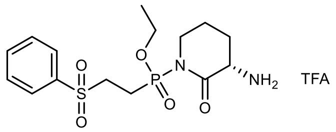

(3*S*)-3-amino-1-[(*R*/*S*)-ethoxy(2-(phenylsulfonyl)ethyl) phosphoryl]piperidin-2-one trifluoroacetate (15). Prepared analogously to compound **14**, but in contrast to the preparation of **14**, before Alloc deprotection, the crude intermediate was oxidized in DCM (0.2 M) with 3-chlorobenzene-1-carboperoxoic acid (2.2 equiv.) for 4 hours at room temperature, to obtain the corresponding sulfon. The resulting reaction mixture was diluted with DCM (30 mL), washed with 5% aqueous sodium thiosulfate (30 mL) and brine (20 mL), dried over Na_2SO_4, filtered, and concentrated *in vacuo*. After Alloc deprotection *via* general procedure **B, 15** was provided as yellow crystals (13 mg, 3%, mixture of two P isomers). LC-MS (ESI) R_t = 4.9 min; *m/z* calcd. for $[C_{15}H_{24}N_2O_5PS]^+$ = 375.1, found 375.5. ^{1}H NMR (400 MHz, DMSO-D$_6$) δ 8.39 (br, 3H), 7.96 – 7.89 (m, 2H), 7.83 – 7.74 (m, 1H), 7.73 – 7.64 (m, 2H), 4.17 – 4.06 (m, 1H), 4.05 – 3.96 (m, 1H), 3.96 – 3.83 (m, 1H), 3.80 – 3.70 (m, 1H), 3.67 – 3.37 (m, 2H), 3.32 – 3.21 (m, 1H), 2.45 – 2.29 (m, 2H), 2.27 – 2.13 (m, 1H), 1.94 – 1.79 (m, 1H), 1.77 – 1.59 (m, 2H), 1.23 – 1.07 (m, 3H). ^{13}C NMR (101 MHz, DMSO-D$_6$) δ 171.7, 171.6, 138.2, 137.9, 134.3, 134.0, 129.7, 129.5, 128.0, 127.9, 61.7 – 61.5 (m), 59.8, 50.6, 49.8 – 49.4 (m), 48.0, 43.5, 42.8, 23.5 (d, *J* = 44.7 Hz), 22.0, 21.2, 21.0, 20.7, 20.2 (d, *J* = 4.2 Hz), 19.9, 19.8 (d, *J* = 3.1 Hz), 19.7, 16.5 (d, *J* = 6.0 Hz), 16.1 – 15.9 (m). HRMS (ESI) *m*/*z* calcd. for $[C_{15}H_{24}N_2O_5PS]^+$ = 375.11381; found 375.11407 (|Δ| = 0.69 ppm).

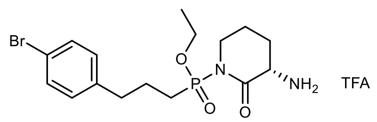

(3*S*)-3-amino-1-[(*R*/*S*)-ethoxy(3-(4-bromophenyl propyl)phosphoryl]piperidin-2-one trifluoroacetate (12). Prepared using compound **SI-9** through general procedure **B**, provided as a white solid (42 mg, 36%, mixture of two P isomers). LC-MS (ESI) R_t = 5.2 min; *m/z* calcd. for $[C_{16}H_{25}BrN_2O_3P]^+$ = 403.1, found 403.8. ^{1}H NMR (400 MHz, DMSO-D_6) δ 8.41 (br, 3H), 7.51 – 7.43 (m, 2H), 7.20 – 7.14 (m, 2H), 4.23 – 4.11 (m, 1H), 4.07 – 3.95 (m, 1H), 3.93 – 3.79 (m, 1H), 3.75 – 3.62 (m, 1H), 3.62 – 3.47 (m, 1H), 2.67 – 2.59 (m, 2H), 2.29 – 2.17 (m, 1H), 2.14 – 1.95 (m, 2H), 1.93 – 1.64 (m, 5H), 1.24 – 1.16 (m, 3H). ^{13}C NMR (101 MHz, DMSO-D_6) δ 171.34, 171.29, 158.8 – 157.3 (m), 140.5, 131.2, 130.74, 130.72, 119.1, 60.8 – 60.5 (m), 49.5, 42.8, 42.4, 34.9, 34.7, 26.5 (d, *J* = 45.6 Hz), 25.2 (d, *J* = 46.3 Hz), 23.5, 23.3, 22.9 – 22.7 (m), 20.2 (d, *J* = 3.1 Hz), 20.0 (d, *J* = 2.6 Hz), 16.1 (d, *J* = 3.5 Hz), 16.0 (d, *J* = 3.3 Hz). HRMS (ESI) *m*/*z* calcd. for $[C_{16}H_{25}BrN_2O_3P]^+$ = 403.07807; found 403.07884 (|Δ| = 1.91 ppm).

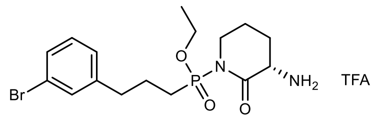

(3*S*)-3-amino-1-[(*R*/*S*)-ethoxy(3-(3-bromophenyl propyl)phosphoryl]piperidin-2-one trifluoroacetate (13). Prepared using compound **SI-9** through general procedure **B**, provided as a white solid (39 mg, 33%, mixture of two P isomers). LC-MS (ESI) R_t = 5.4 min; *m/z* calcd. for $[C_{16}H_{25}BrN_2O_3P]^+$ = 403.1, found 403.2. ^{1}H NMR (400 MHz, DMSO-D_6) δ 8.39 (br, 3H), 7.44 – 7.41 (m, 1H), 7.41 – 7.38 (m, 1H), 7.28 – 7.24 (m, 1H), 7.23 – 7.20 (m, 1H), 4.23 – 4.11 (m, 1H), 4.07 – 3.94 (m, 1H), 3.94 – 3.80 (m, 1H), 3.75 – 3.45 (m, 2H), 2.69 – 2.60 (m, 2H), 2.28 – 2.18 (m, 1H), 2.13 – 1.95 (m, 2H), 1.93 – 1.63 (m, 5H), 1.24 – 1.16 (m, 3H). ^{13}C NMR (101 MHz, DMSO-D_6) δ 171.4, 171.3, 158.6 – 157.5 (m), 144.0, 131.2, 130.6, 129.0, 127.6, 127.6, 121.7, 121.3 – 112.4 (m), 60.9 – 60.5 (m), 49.5, 42.8, 42.4, 35.1 (d, *J* = 4.3 Hz), 34.9 (d, *J* = 4.0 Hz), 26.5 (d, *J* = 46.6 Hz), 25.3 (d, *J* = 47.6 Hz), 23.5, 23.3, 22.9, 20.2 (d, *J* = 3.4 Hz), 20.0 (d, *J* = 3.1 Hz), 16.1 (d, *J* = 3.6 Hz), 16.0 (d, *J* = 3.2 Hz). HRMS (ESI) *m*/*z* calcd. for $[C_{16}H_{25}BrN_2O_3P]^+$ = 403.07807; found 403.07764 (|Δ| = 1.07 ppm).

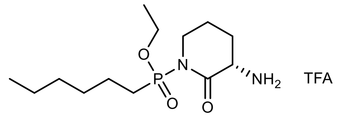

(3*S*)-3-amino-1-[(*R*/*S*)-ethoxy(hexyl)phosphoryl] piperidin-2-one trifluoroacetate (5). Prepared using hexylphosphonic acid diethyl ester (**2**) through general procedure **B**, provided as clear colorless oil (18 mg, 27%, mixture of two P isomers). LC-MS (ESI) R_t = 5.2 min; *m/z*

calcd. for $[C_{13}H_{28}N_2O_3P]^+$ = 291.2, found 291.5. ^{1}H NMR (400 MHz, DMSO-D$_6$) δ 8.43 (br, 3H), 4.23 – 4.11 (m, 1H), 4.08 – 3.94 (m, 1H), 3.93 – 3.80 (m, 1H), 3.75 – 3.61 (m, 1H), 3.61 – 3.44 (m, 1H), 2.30 – 2.18 (m, 1H), 2.13 – 1.95 (m, 2H), 1.94 – 1.64 (m, 3H), 1.53 – 1.37 (m, 2H), 1.37 – 1.28 (m, 2H), 1.27 – 1.16 (m, 7H), 0.85 (t, *J* = 6.8 Hz, 3H). ^{13}C NMR (101 MHz, DMSO-D$_6$) δ 171.3, 158.3 (q, *J* = 32.9 Hz), 116.7 (q, *J* = 297.0 Hz), 60.7 – 60.4 (m), 49.5, 42.5 (d, *J* = 24.3 Hz), 30.8, 29.5 (d, *J* = 14.0 Hz), 26.9 (d, *J* = 41.6 Hz), 25.7 (d, *J* = 42.3 Hz), 23.4 (d, *J* = 6.7 Hz), 21.9, 21.2 (d, *J* = 4.4 Hz), 21.1 (d, J = 4.4 Hz), 20.18 (d, *J* = 3.5 Hz), 20.02 (d, *J* = 2.9 Hz), 16.1 (d, I = 3.0 Hz), 16.0 (d, *J* = 2.8 Hz), 13.9. HRMS (ESI) *m/z* calcd. for $[C_{13}H_{28}N_2O_3P]^+$ = 291.18321; found 291.18361 (|Δ| = 1.37 ppm).

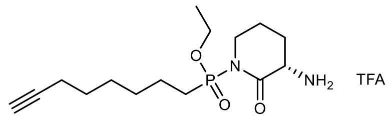

(3*S*)-3-amino-1-[(*R*/*S*)-ethoxy(oct-7-ynyl) phosphoryl]piperidin-2-one trifluoroacetate (10). Prepared using compound **SI-11** through general procedure **B**, provided as clear colorless oil (5 mg, 7%, mixture of two P isomers). LC-MS (ESI) R_t = 5.2 min; *m/z* calcd. for $[C_{15}H_{28}N_2O_3P]^+$ = 315.2, found 315.3. ^{1}H NMR (400 MHz, DMSO-D$_6$) δ 8.31 (br, 3H), 4.26 – 4.11 (m, 1H), 4.07 – 3.93 (m, 1H), 3.93 – 3.79 (m, 1H), 3.79 – 3.61 (m, 1H), 3.61 – 3.44 (m, 1H), 2.75 (t, *J* = 2.6 Hz, 1H), 2.23 (m, 1H), 2.14 (td, *J* = 7.0, 2.2 Hz, 2H), 2.09 – 1.95 (m, 2H), 1.92 – 1.75 (m, 2H), 1.75 – 1.60 (m, 1H), 1.52 – 1.38 (m, 4H), 1.37 – 1.28 (m, 4H), 1.26 – 1.15 (m, 3H). ^{13}C NMR (101 MHz, DMSO-D$_6$) δ 171.5 – 171.2 (m), 84.5, 71.3, 60.8 – 60.5 (m), 49.7 – 49.2 (m), 42.6, 42.3, 29.3, 29.2, 27.7, 27.7, 26.9 (d, *J* = 41.8 Hz), 25.7 (d, *J* = 43.4 Hz), 23.4, 23.3, 21.5 – 21.0 (m), 20.3 – 20.0 (m), 20.0 – 19.8 (m), 17.6, 16.2 – 15.8 (m). HRMS (ESI) *m/z* calcd. for $[C_{15}H_{28}N_2O_3P]^+$ = 315.18321; found 315.18339 (|Δ| = 0.57 ppm).

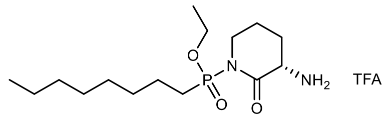

(3*S*)-3-amino-1-[(*R*/*S*)-ethoxy(octyl)phosphoryl] piperidin-2-one trifluoroacetate (6). Prepared using compound **SI-12** through general procedure **B**, provided as clear colorless oil (29 mg, 39%, mixture of two P isomers). LC-MS (ESI) R_t = 5.4 min; *m/z* calcd. for $[C_{15}H_{32}N_2O_3P]^+$ = 319.2, found 319.7. ^{1}H NMR (400 MHz, DMSO-D$_6$) δ 8.33 (br, 3H), 4.26 – 4.11 (m, 1H), 4.11 – 3.92 (m, 1H), 3.92 – 3.78 (m, 1H), 3.78 – 3.62 (m, 1H), 3.60 – 3.43 (m, 1H), 2.29 – 2.17 (m, 1H), 2.15 – 1.96 (m, 2H), 1.96 – 1.74 (m, 2H), 1.74 – 1.60 (m, 1H), 1.53 – 1.38 (m, 2H), 1.37 – 1.22 (m, 10H), 1.22 – 1.12 (m, 3H), 0.94 –

0.79 (m, 3H). ^{13}C NMR (101 MHz, DMSO-D$_6$) δ 171.3, 158.0 (q, *J* = 32.1 Hz), 116.8 (q, *J* = 297.9 Hz), 61.0 – 60.3 (m), 49.5, 42.6, 42.3, 31.2, 29.9, 29.7, 28.7 – 28.3 (m), 26.9 (d, *J* = 41.3 Hz), 25.7 (d, *J* = 41.6 Hz), 23.5 – 23.2 (m), 22.1, 21.4 – 21.0 (m), 20.3 – 20.0 (m), 20.0 – 19.7 (m), 16.1, 14.0. HRMS (ESI) *m/z* calcd. for $[C_{15}H_{32}N_2O_3P]^+$ = 319.21451; found 319.21502 (|Δ| = 1.60 ppm).

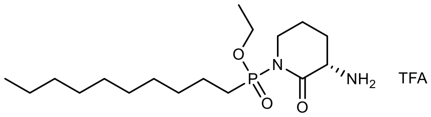

(3*S*)-3-amino-1-[(*R*/*S*)-decyl(ethoxy)phosphoryl] piperidin-2-one trifluoroacetate (7). Prepared using decylphosphonic acid diethyl ester through general procedure **B**, provided as clear colorless oil (37 mg, 47%, mixture of two P isomers). LC-MS (ESI) R_t = 5.7 min; *m/z* calcd. for $[C_{17}H_{36}N_2O_3P]^+$ = 347.2, found 347.5. ^{1}H NMR (400 MHz, DMSO-D$_6$) δ 8.35 (br, 3H), 4.24 – 4.11 (m, 1H), 4.09 – 3.93 (m, 1H), 3.93 – 3.78 (m, 1H), 3.78 – 3.62 (m, 1H), 3.62 – 3.39 (m, 1H), 2.28 – 2.17 (m, 1H), 2.15 – 1.96 (m, 2H), 1.96 – 1.75 (m, 2H), 1.75 – 1.61 (m, 1H), 1.53 – 1.36 (m, 2H), 1.36 – 1.22 (m, 14H), 1.23 – 1.15 (m, 3H), 0.85 (t, *J* = 6.8 Hz, 3H). ^{13}C NMR (101 MHz, DMSO-D$_6$) δ 171.3, 158.1 (q, *J* = 34.0 Hz), 116.4 (q, *J* = 297.2 Hz), 60.8 – 60.5 (m), 49.5, 42.6, 42.3, 31.3, 29.9, 29.7, 29.0, 28.9, 28.7, 28.6 – 28.5 (m), 26.9 (d, *J* = 40.8 Hz), 25.7 (d, *J* = 41.6 Hz), 23.3, 22.1, 21.3 – 21.1 (m), 20.2 – 20.1 (m), 20.1 – 19.9 (m), 16.1 – 15.9 (m), 14.0. HRMS (ESI) *m/z* calcd. for $[C_{17}H_{36}N_2O_3P]^+$ = 347.24581; found 347.24624 (|Δ| = 1.24 ppm).

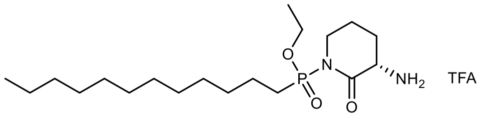

(3*S*)-3-amino-1-[(*R*/*S*)-dodecyl(ethoxy) phosphoryl]piperidin-2-one trifluoroacetate (8). Prepared using dodecylphosphonic acid diethyl ester through general procedure **B**, provided as clear colorless oil (23 mg, 26%, mixture of two P isomers). LC-MS (ESI) R_t = 6.0 min; *m/z* calcd. for $[C_{19}H_{40}N_2O_3P]^+$ = 375.3, found 375.6. ^{1}H NMR (400 MHz, DMSO-D$_6$) δ 8.37 (br, 3H), 4.24 – 4.11 (m, 1H), 4.10 – 3.77 (m, 2H), 3.76 – 3.43 (m, 4H), 2.30 – 2.16 (m, 1H), 2.15 – 1.93 (m, 2H), 1.95 – 1.73 (m, 2H), 1.75 – 1.60 (m, 1H), 1.53 – 1.37 (m, 2H), 1.28 – 1.22 (m, 16H), 1.22 – 1.15 (m, 3H), 0.90 – 0.80 (m, 3H). ^{13}C NMR (101 MHz, DMSO-D$_6$) δ 171.3, 158.1 (q, *J* = 32.8 Hz), 60.7 – 60.4 (m), 49.5, 42.6, 42.3, 31.3, 29.9, 29.7, 29.0 (m), 28.8, 28.7, 28.6, 26.9 (d, *J* = 40.6 Hz), 25.7 (d, *J* = 41.4 Hz), 23.6 – 23.2 (m), 22.1, 21.2 (m), 20.1 (m), 16.1 (m), 14.0. HRMS (ESI) *m/z* calcd. for $[C_{19}H_{40}N_2O_3P]^+$ = 375.27711; found 375.27753 (|Δ| = 1.12 ppm).

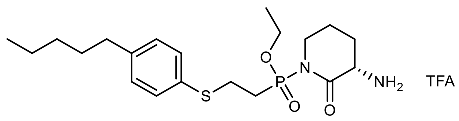

(3*S*)-3-amino-1-[(*R*/*S*)-ethoxy(2-(4-pentylphenylthio) ethyl)phosphoryl] piperidin-2-one trifluoroacetate (16). Prepared using compound **SI-14** through general procedure **B**, provided as a white solid (18 mg, 15%, mixture of two P isomers). LC-MS (ESI) R_t = 5.8 min; *m/z* calcd. for $[C_{20}H_{34}N_2O_3PS]^+$ = 413.2, found 413.7. ^{1}H NMR (400 MHz, DMSO- D_6) δ 8.35 (br, 3H), 7.26 (dd, *J* = 8.2, 1.7 Hz, 2H), 7.18 (d, *J* = 8.2 Hz, 2H), 4.23 – 4.08 (m, 1H), 4.09 – 3.96 (m, 1H), 3.96 – 3.83 (m, 1H), 3.75 – 3.62 (m, 1H), 3.62 – 3.48 (m, 1H), 3.15 – 2.98 (m, 2H), 2.58 – 2.51 (m, 2H), 2.44 – 2.28 (m, 2H), 2.28 – 2.17 (m, 1H), 1.94 – 1.80 (m, 2H), 1.78 – 1.63 (m, 1H), 1.54 (dq, *J* = 9.0, 7.5 Hz, 2H), 1.36 – 1.24 (m, 4H), 1.20 (m, 3H), 0.86 (t, *J* = 7.0 Hz, 3H). ^{13}C NMR (101 MHz, DMSO-D_6) δ 171.4, 158.6 – 157.2 (m), 140.9, 140.9, 131.4, 129.4, 129.3, 129.2, 121.8 – 110.8 (m), 61.1 (m), 49.7 – 49.4 (m), 43.0, 42.6, 34.6, 30.9, 30.5, 27.8 (d, *J* = 68.6 Hz), 26.6 (d, *J* = 69.4 Hz), 25.6 – 25.4 (m), 23.5 (d, *J* = 17.6 Hz), 21.9, 20.2, 20.0, 16.2 – 15.9 (m), 13.9. HRMS (ESI) *m*/*z* calcd. for $[C_{20}H_{34}N_2O_3PS]^+$ = 413.20223; found 413.20294 (|Δ| = 1.72 ppm).

NMR Spectra

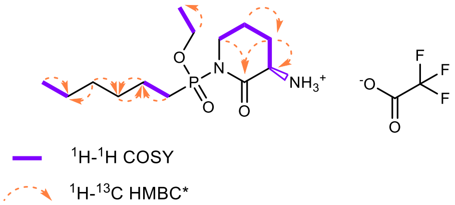

Observed ^{1}H-^{1}H and ^{1}H-^{13}C NMR correlations in typical *N*-phosphonopiperidone example **5**. *Displaying only a limited selection of correlations for clarity.

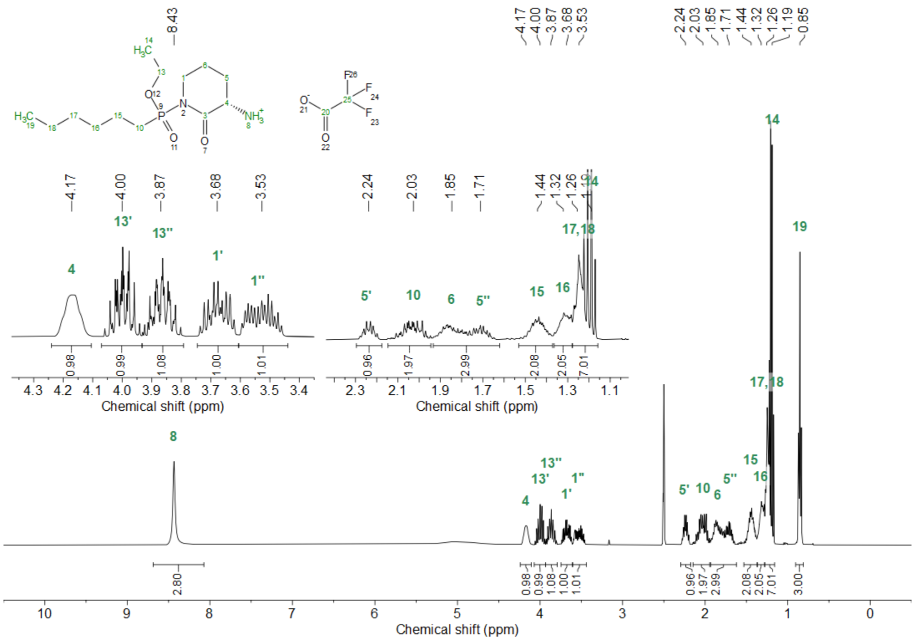

<u>**5** ^{1}H-NMR</u>

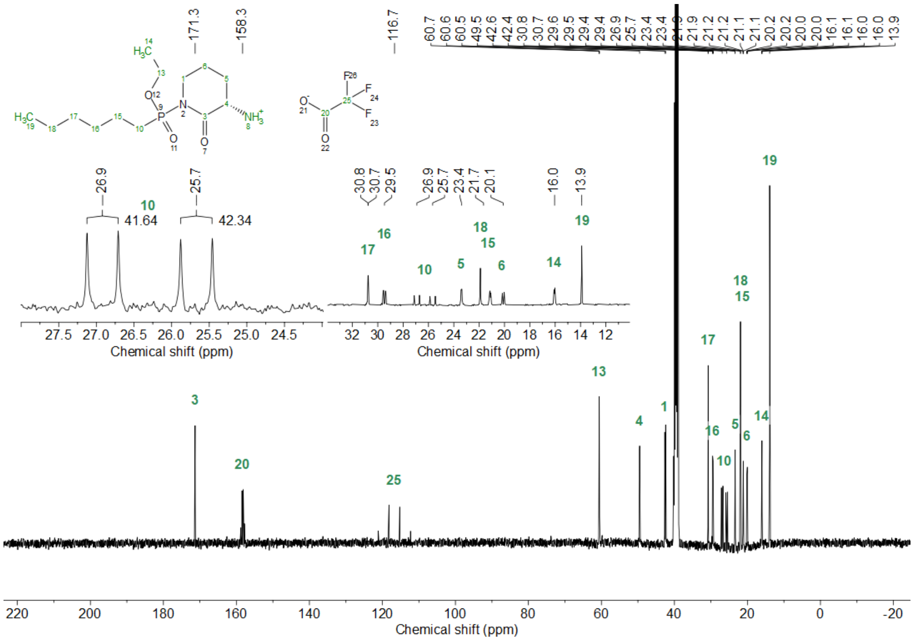

5 ^{13}C-NMR

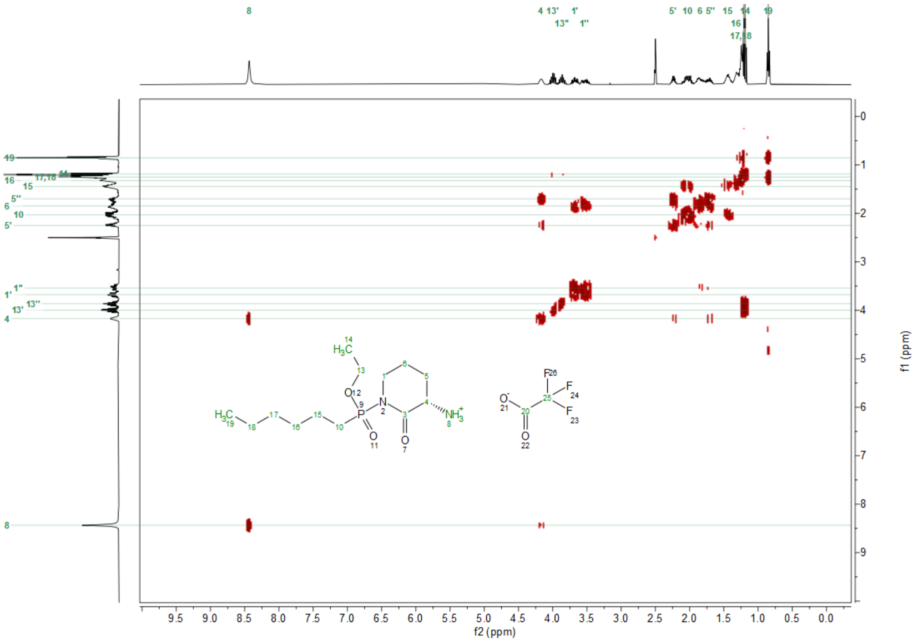

5 ^{1}H-^{1}H COSY NMR

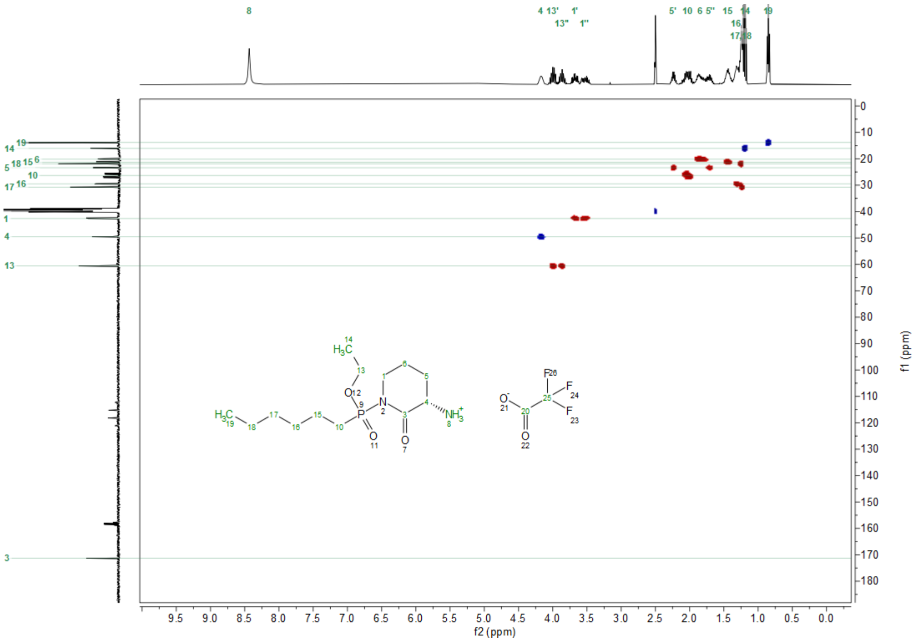

5 ^{1}H-^{13}C HSQC NMR

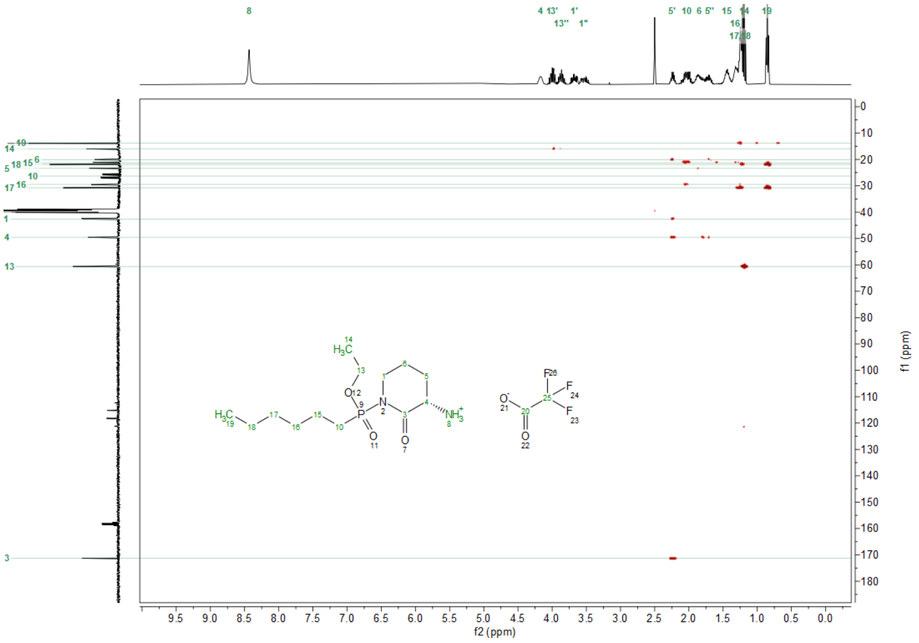

5 ^{1}H-^{13}C HMBC NMR

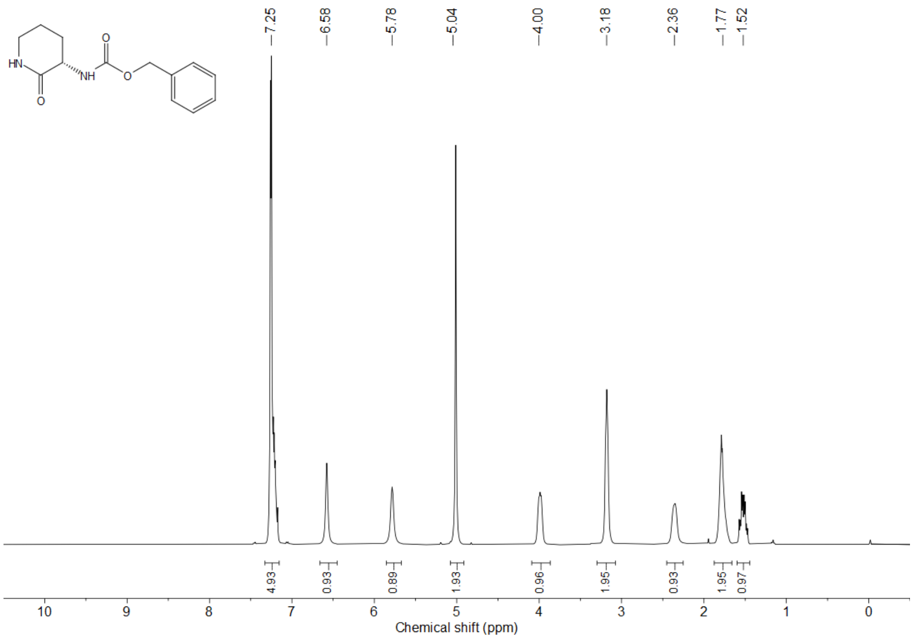

SI-1 ^{1}H-NMR

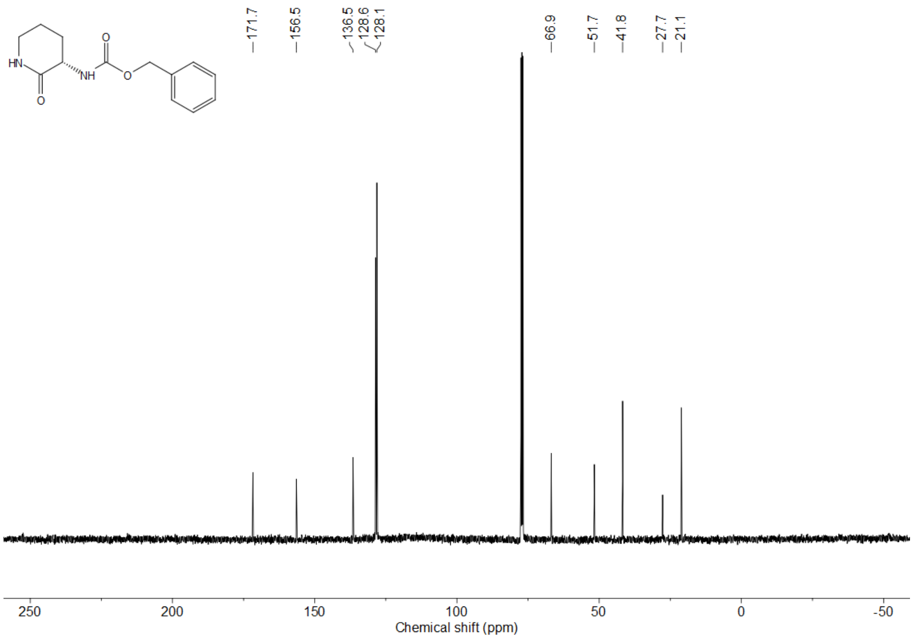

SI-1 ^{13}C-NMR

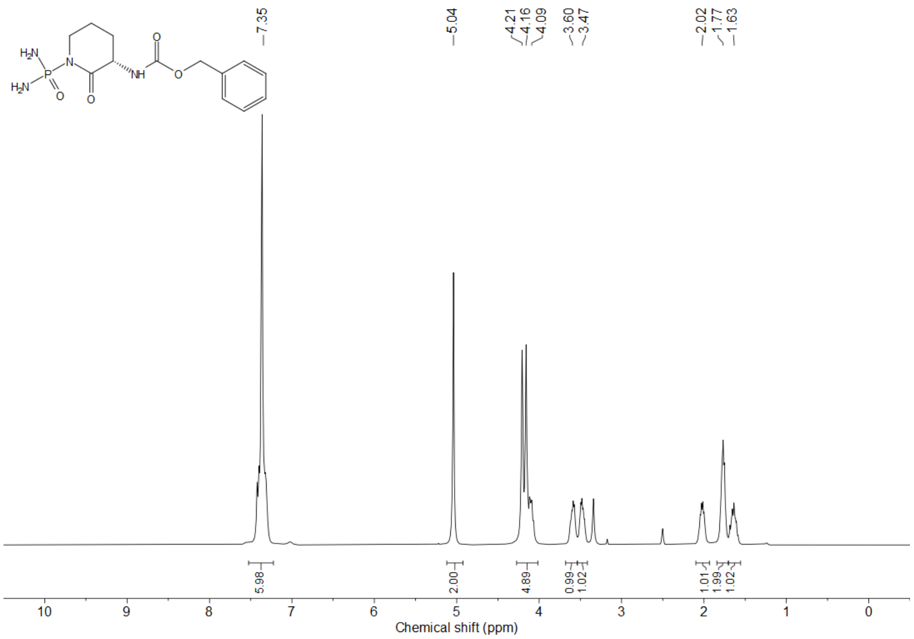

SI-2 1H-NMR

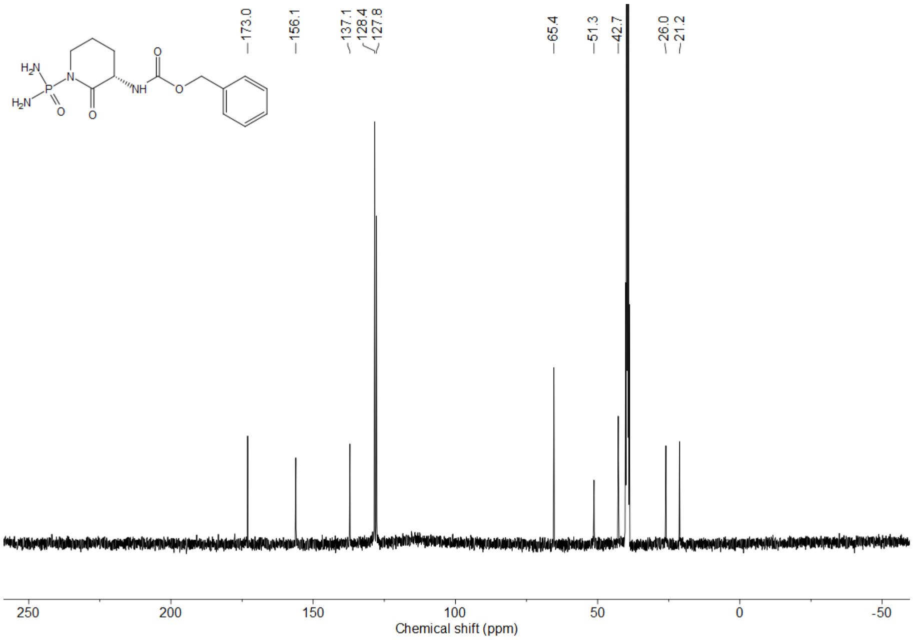

SI-2 13C-NMR

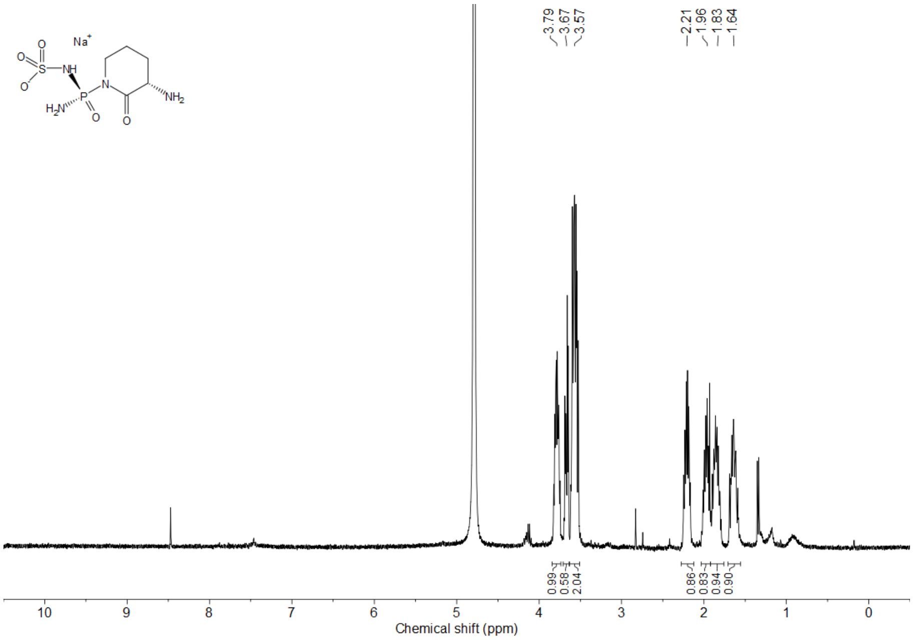

1 ^{1}H-NMR

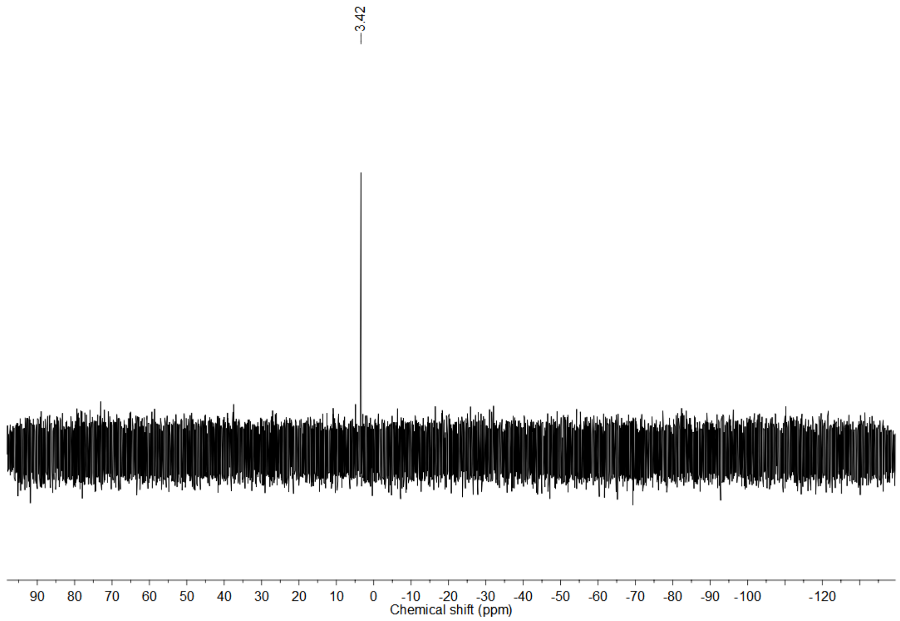

1 ^{31}P-NMR

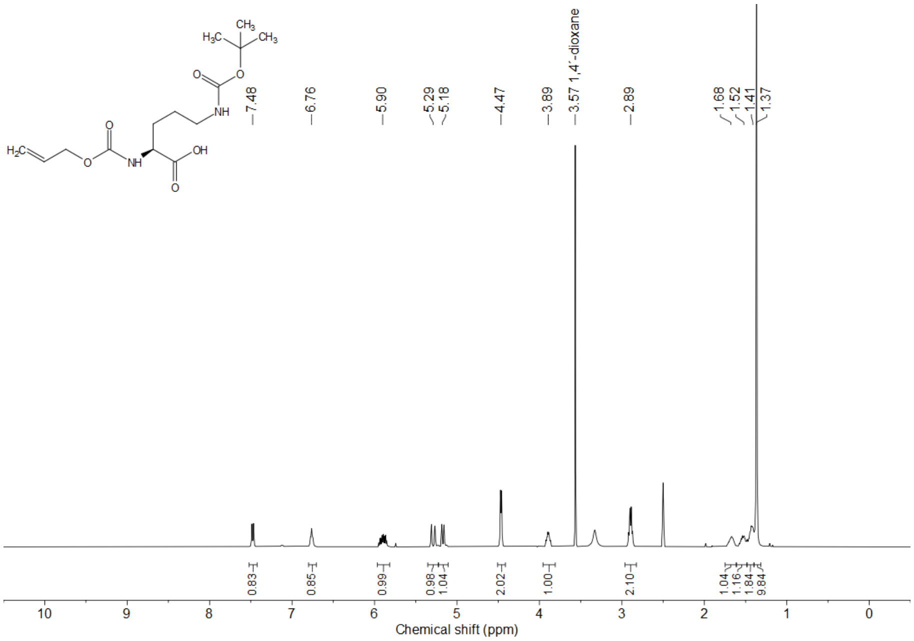

SI-18 ^{1}H-NMR

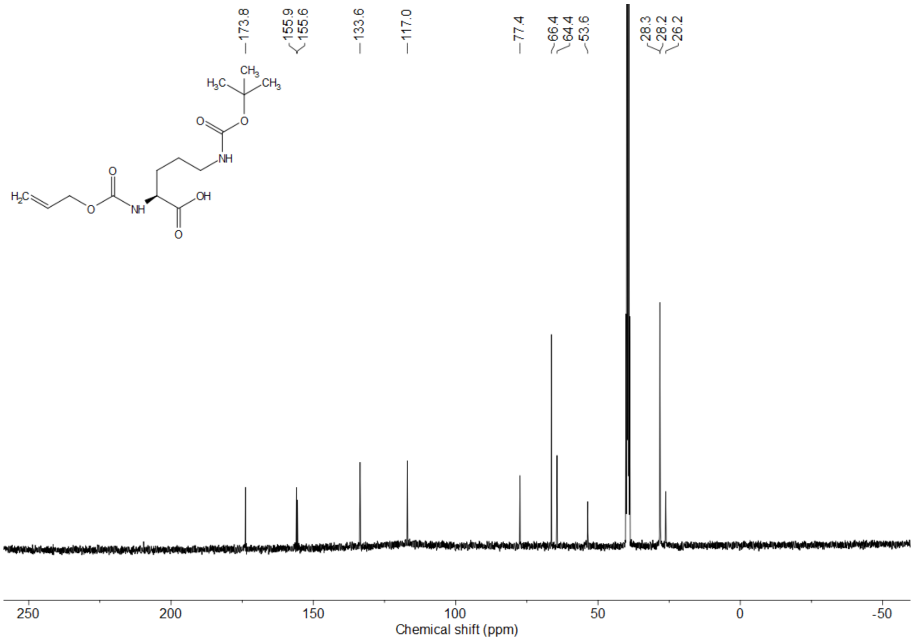

SI-18 ^{13}C-NMR

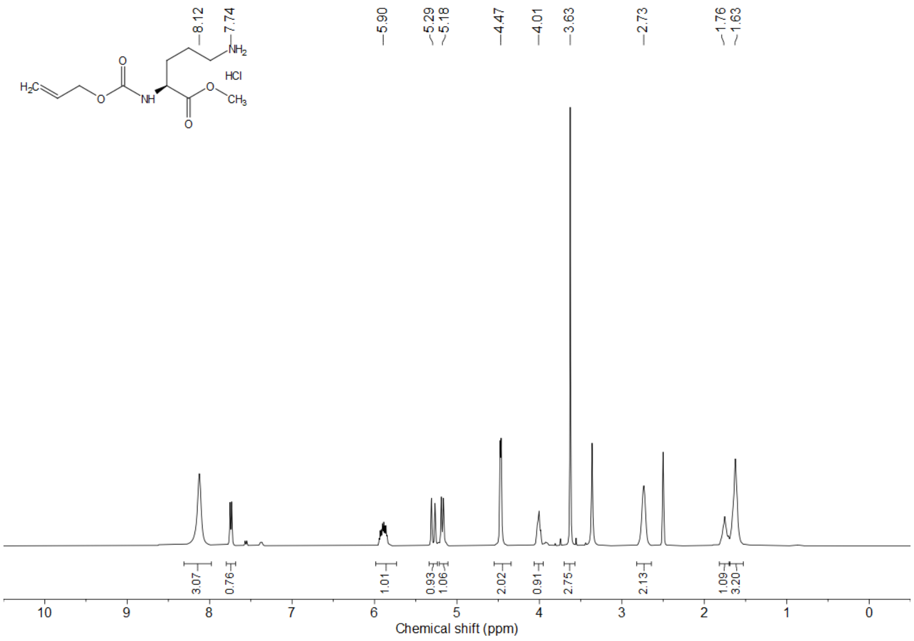

SI-19 ^{1}H-NMR

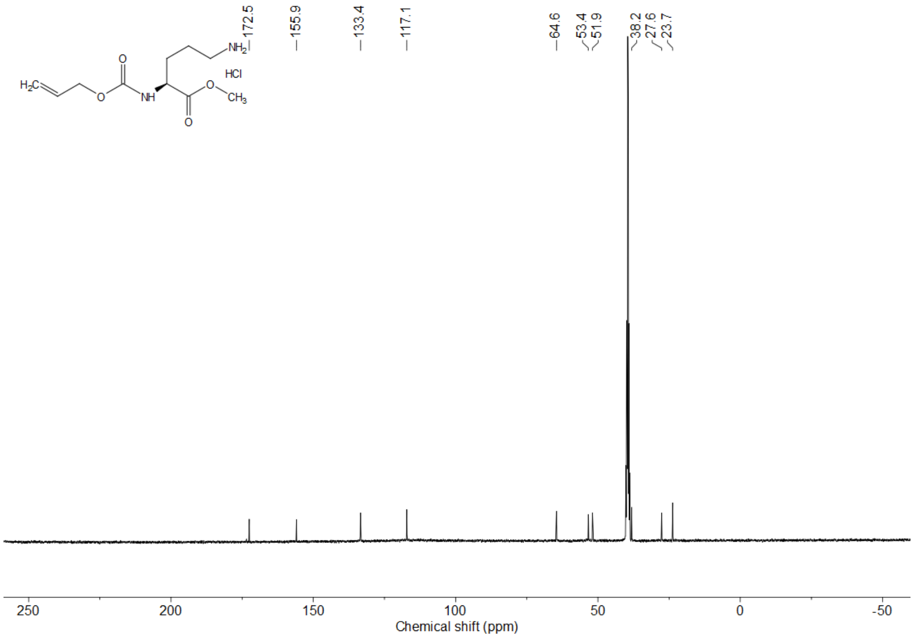

SI-19 ^{13}C-NMR

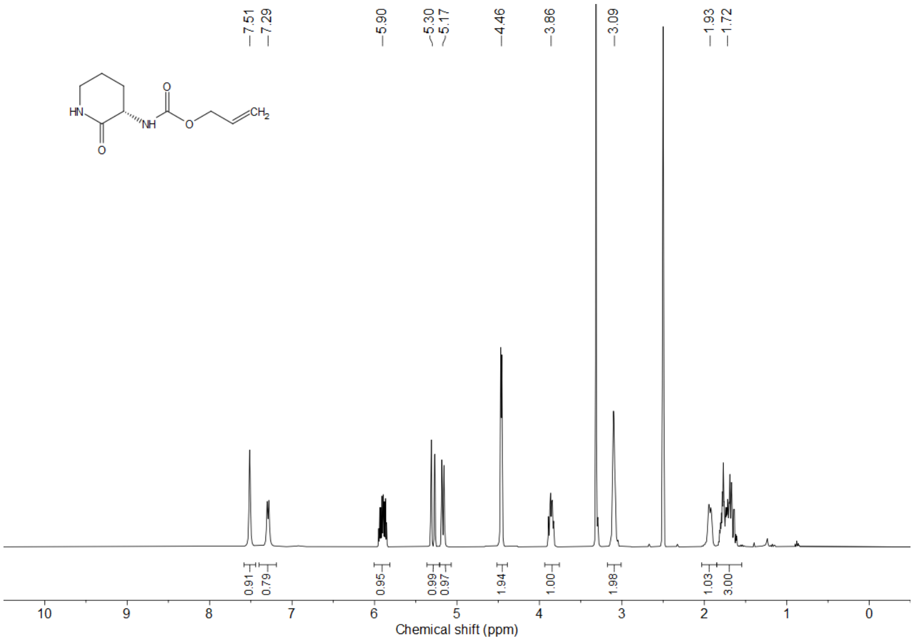

4 ^{1}H-NMR

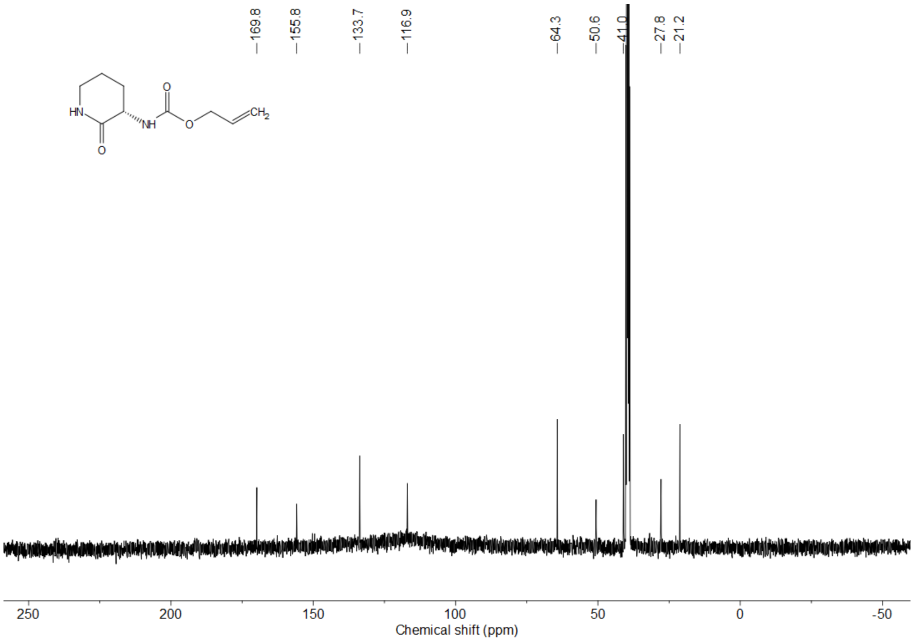

4 ^{13}C-NMR

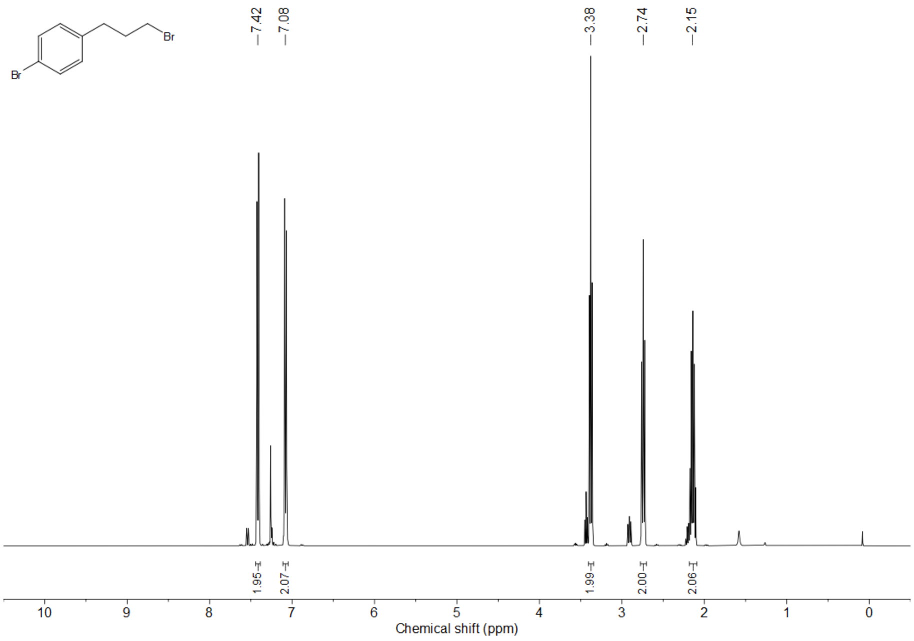

SI-4 ^{1}H-NMR

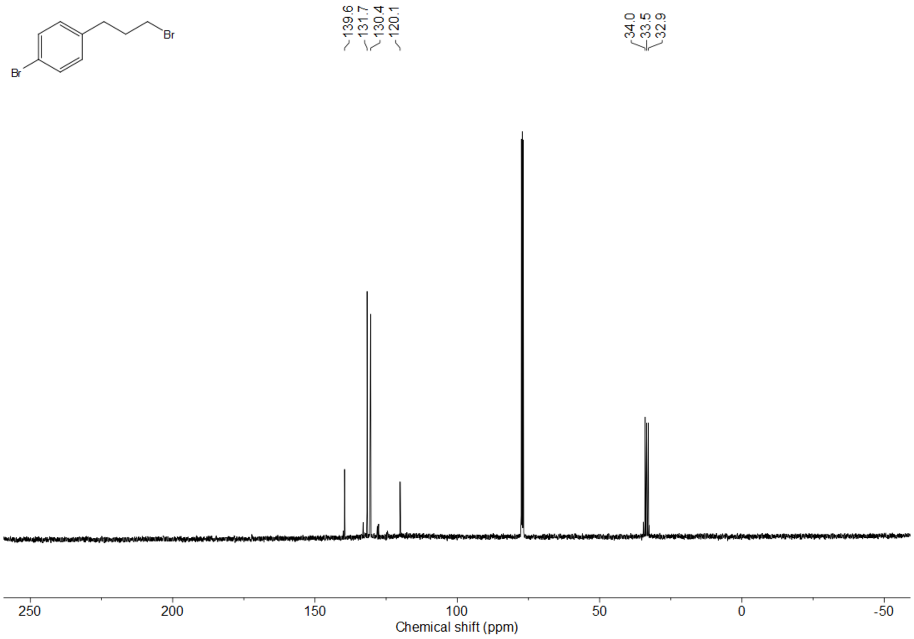

SI-4 ^{13}C-NMR

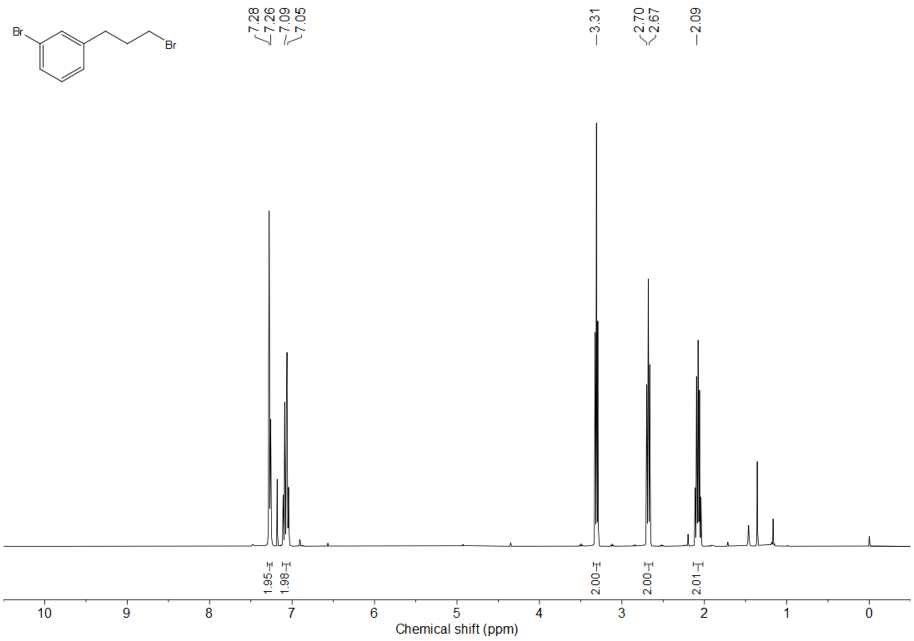

SI-6 ^{1}H-NMR

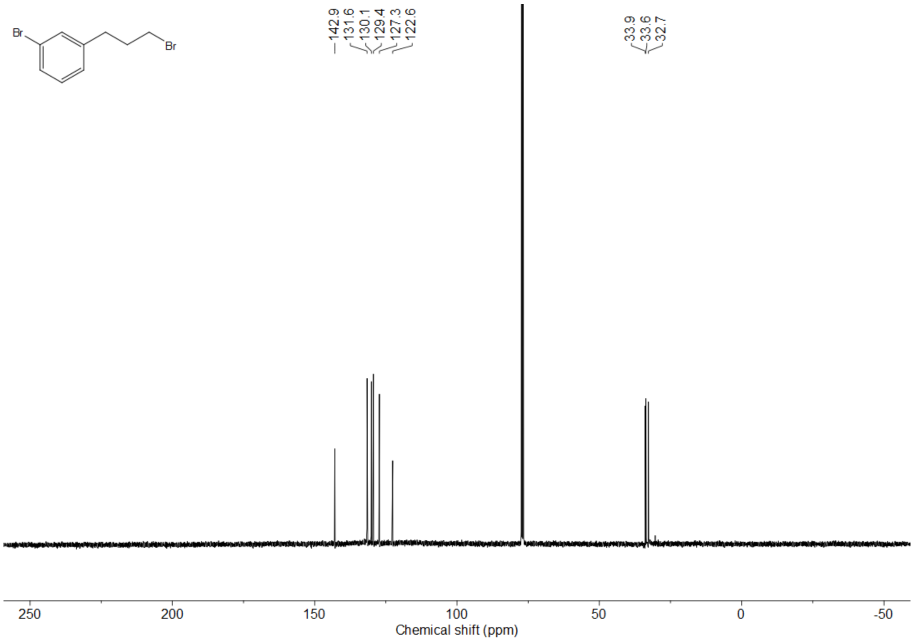

SI-6 ^{13}C-NMR

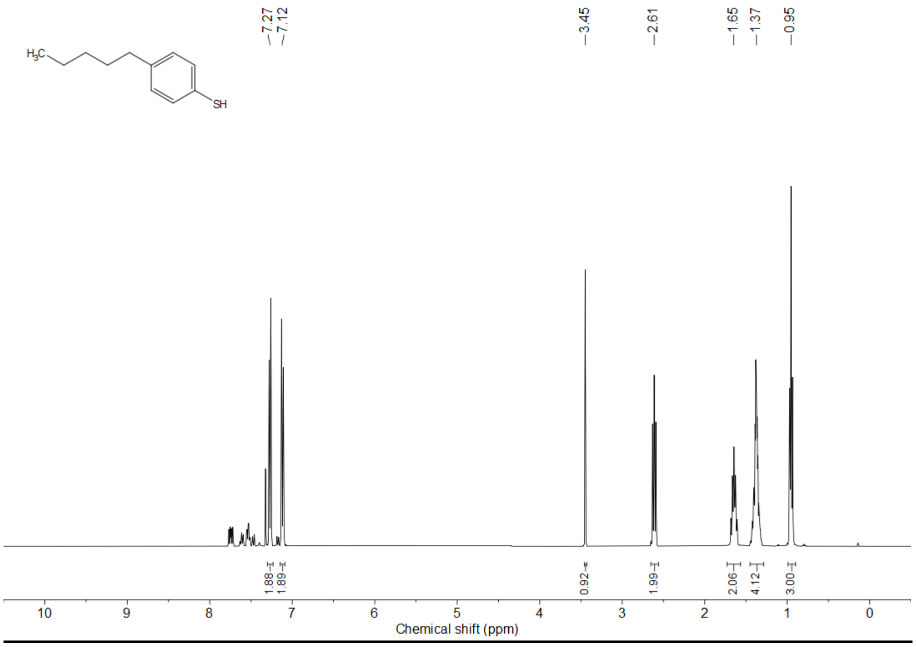

SI-16 ^{1}H-NMR

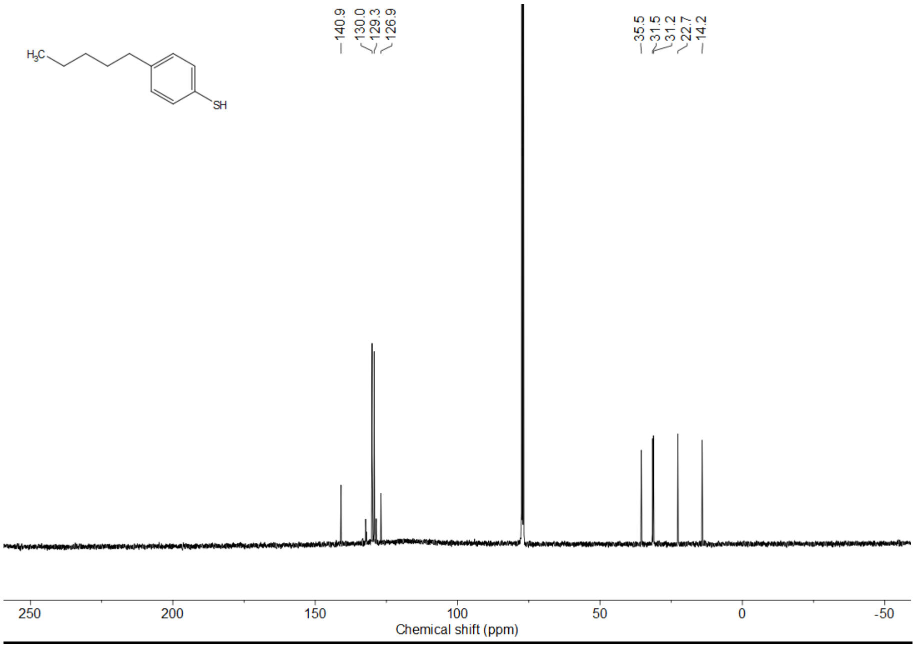

SI-16 ^{13}C-NMR

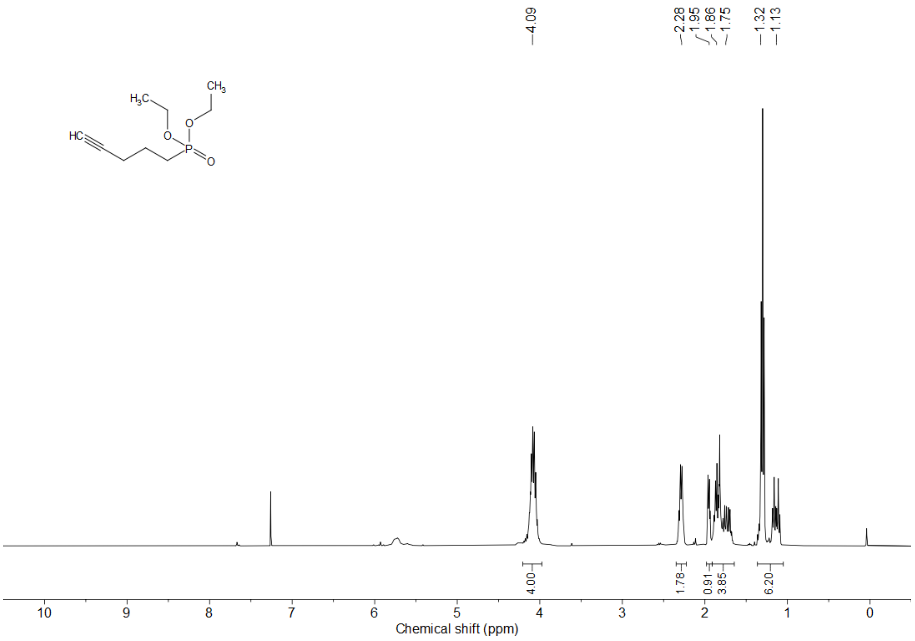

SI-7 ^{1}H-NMR

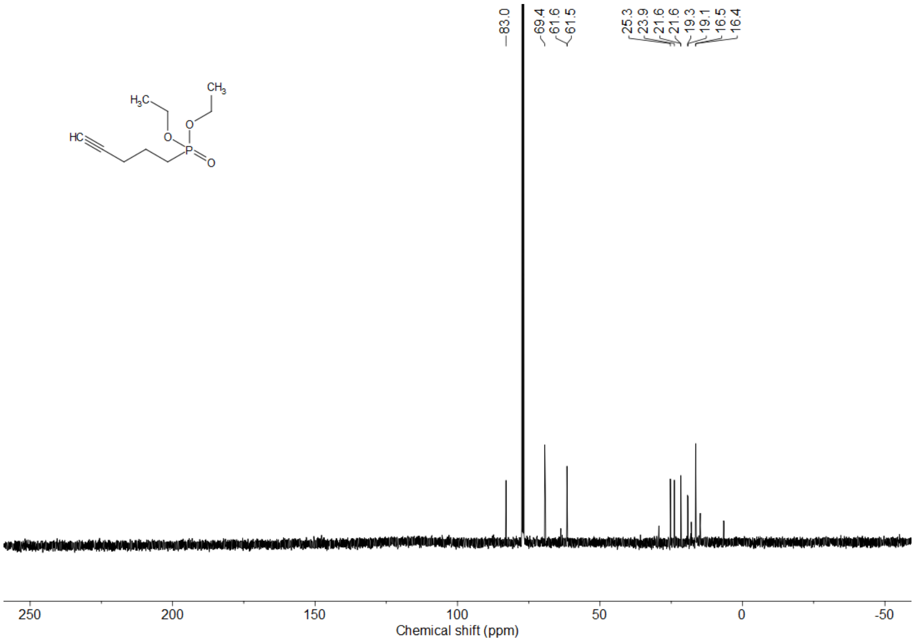

SI-7 ^{13}C-NMR

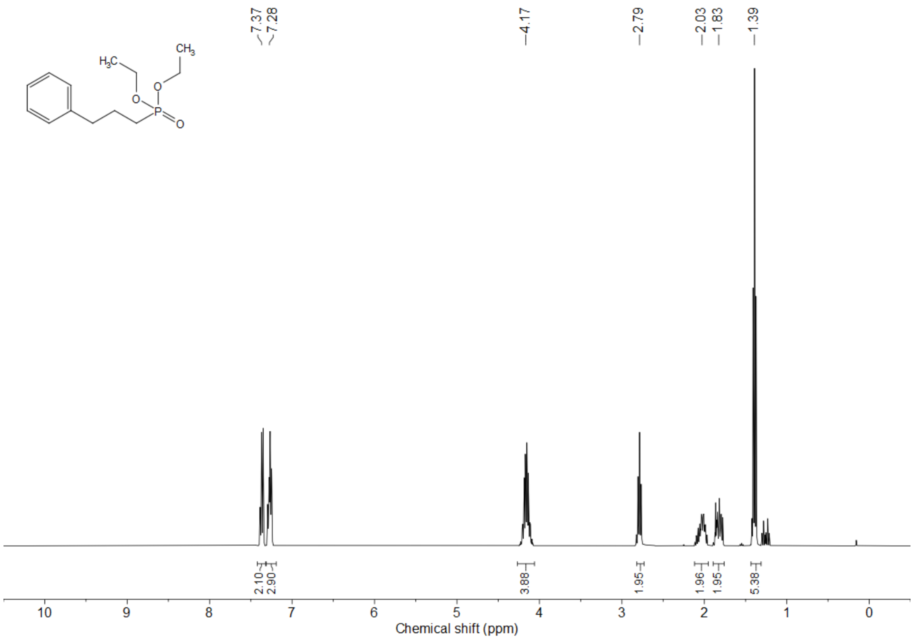

SI-8 ^{1}H-NMR

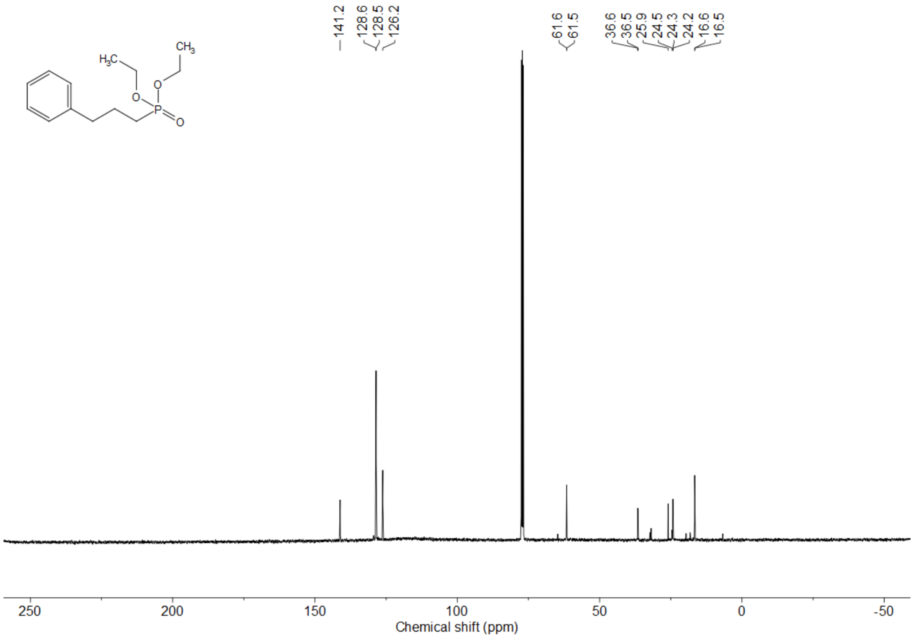

SI-8 ^{13}C-NMR

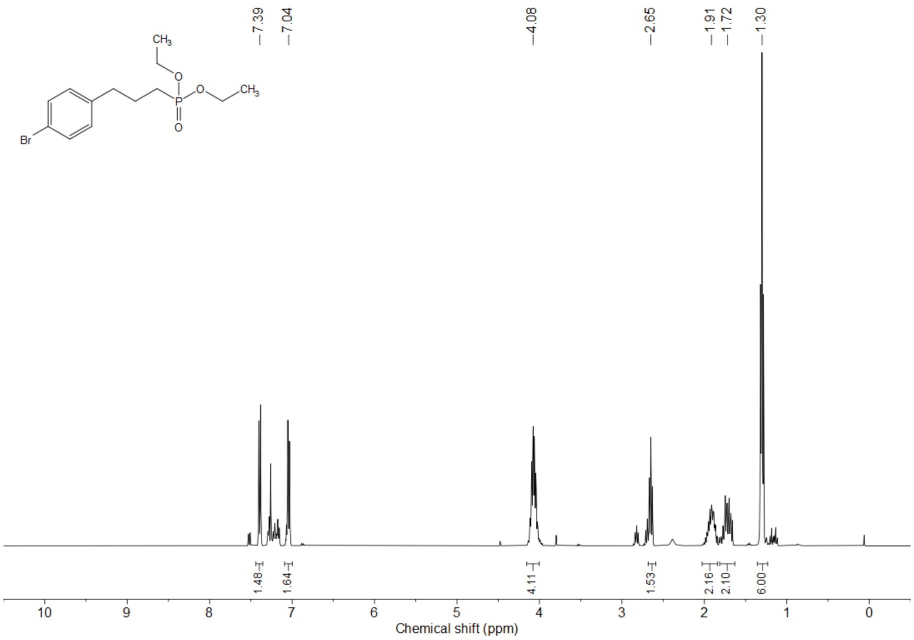

SI-9 1H-NMR

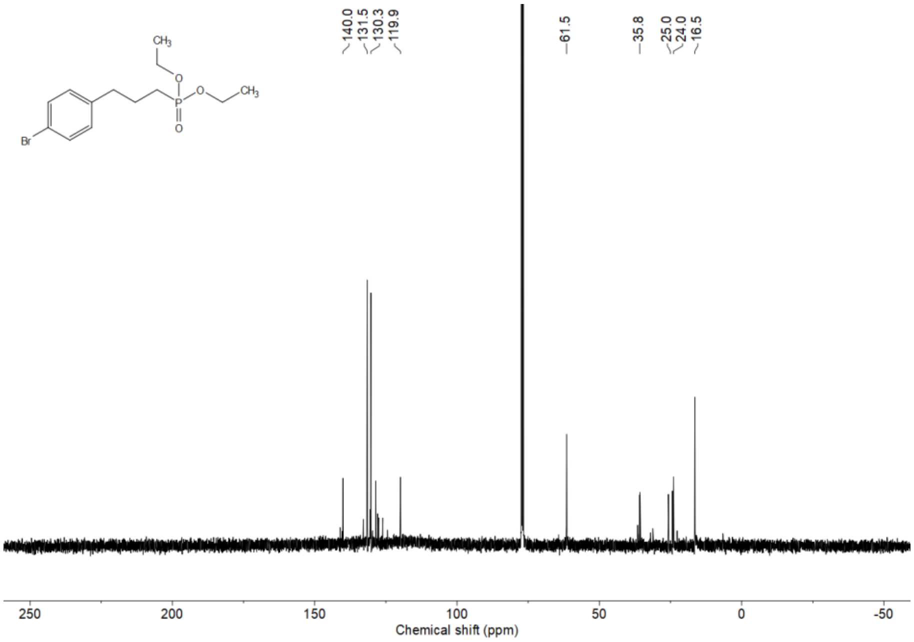

SI-9 13C-NMR

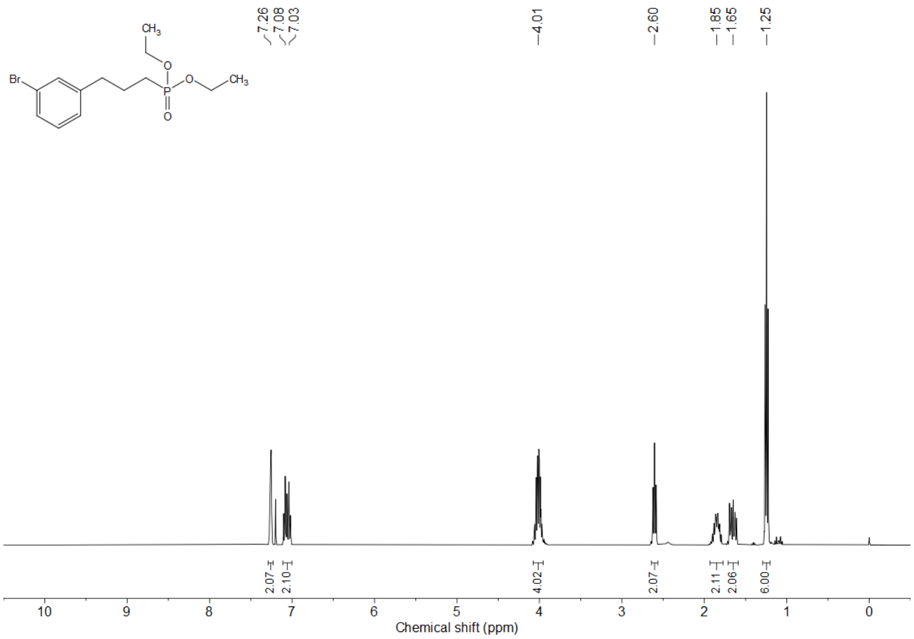

SI-10 ^{1}H-NMR

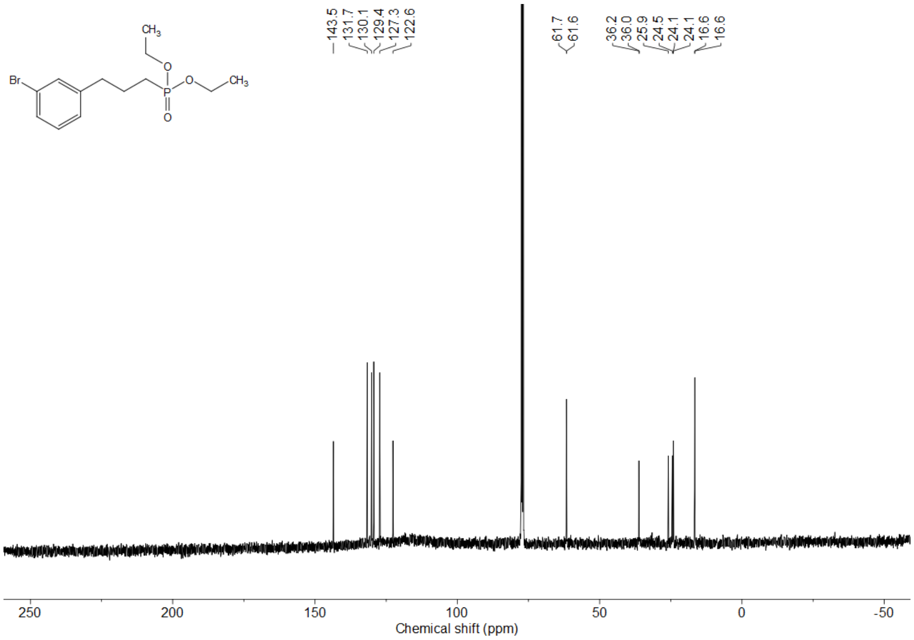

SI-10 ^{13}C-NMR

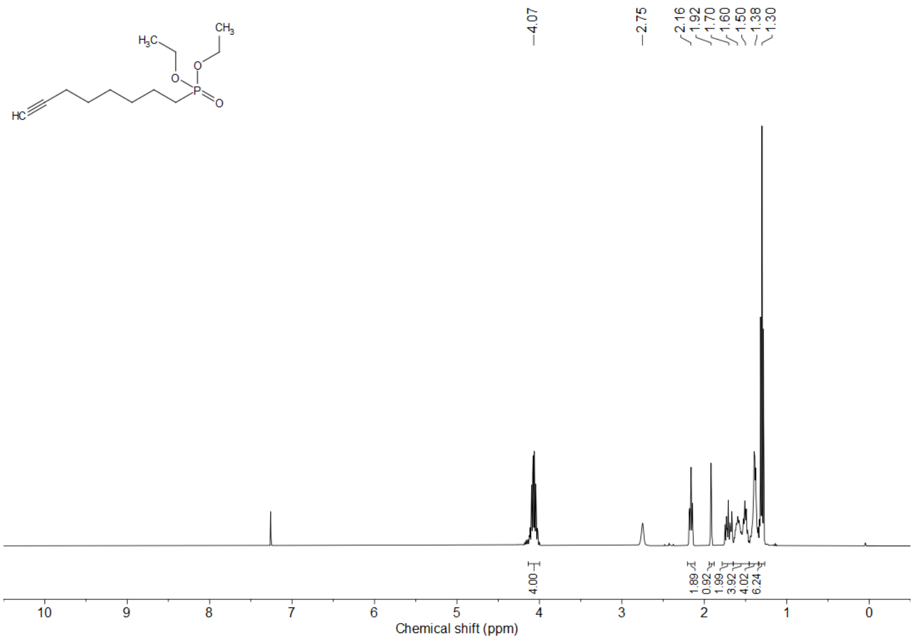

SI-11 ^{1}H-NMR

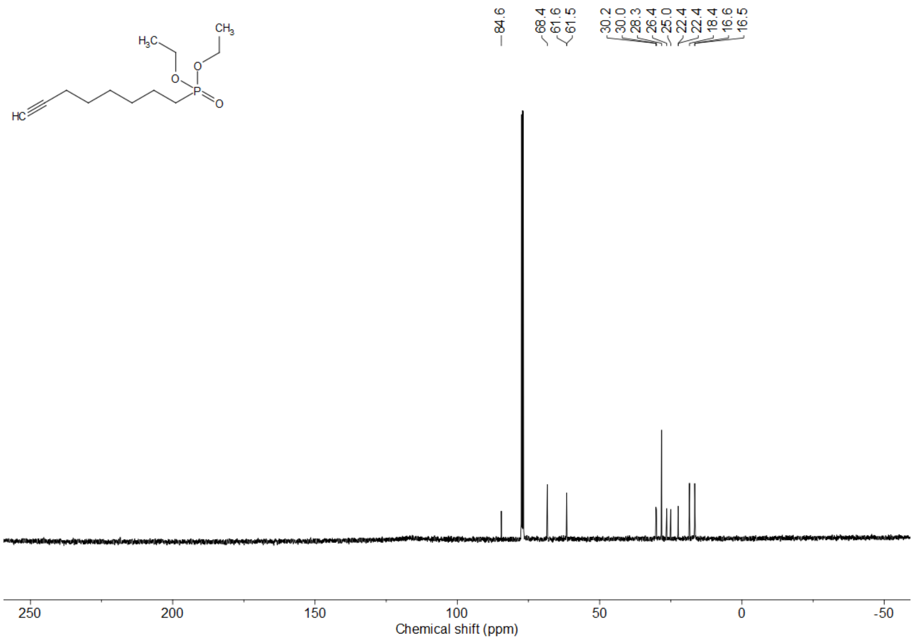

SI-11 ^{13}C-NMR

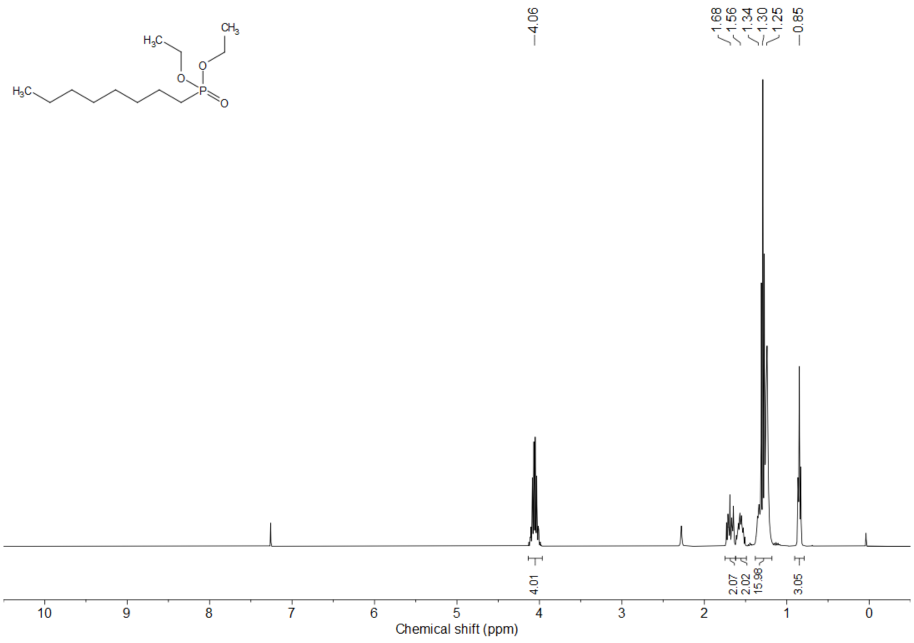

SI-12 ^{1}H-NMR

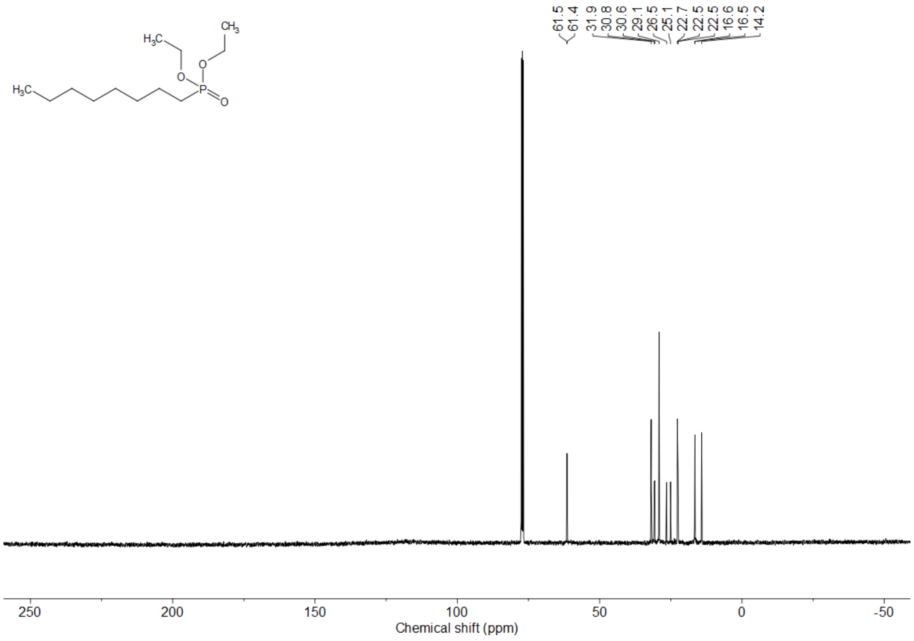

SI-12 ^{13}C-NMR

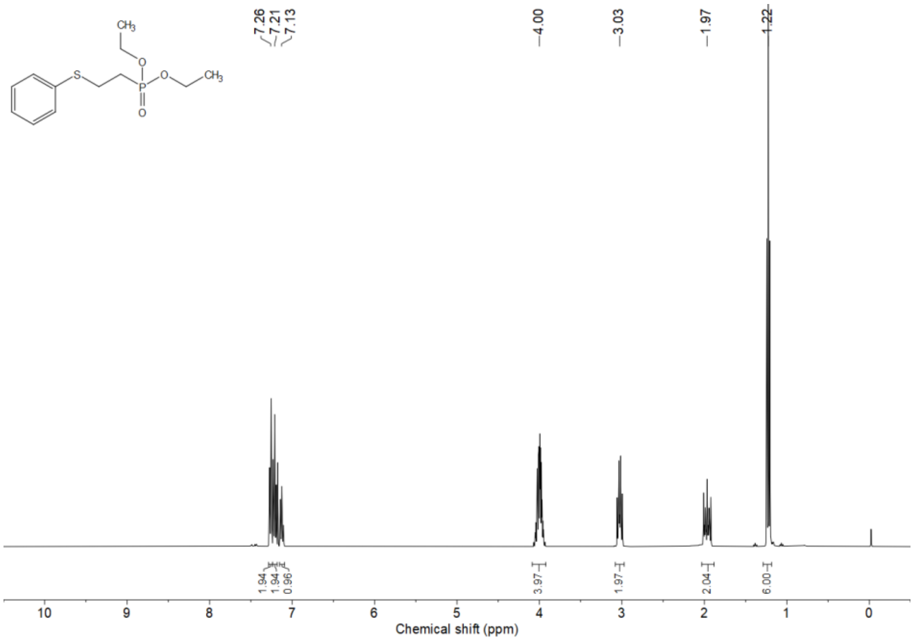

SI-13 ^{1}H-NMR

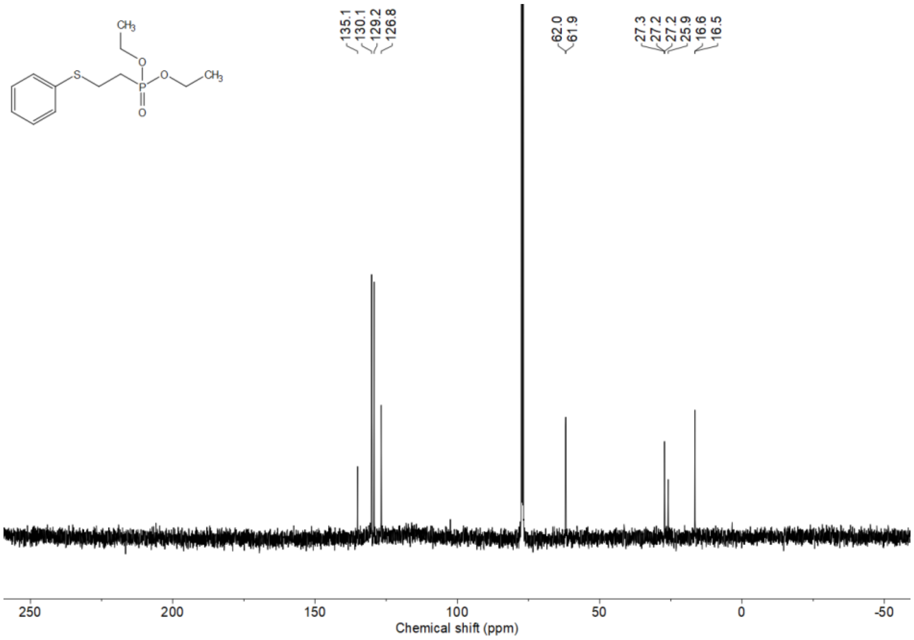

SI-13 ^{13}C-NMR

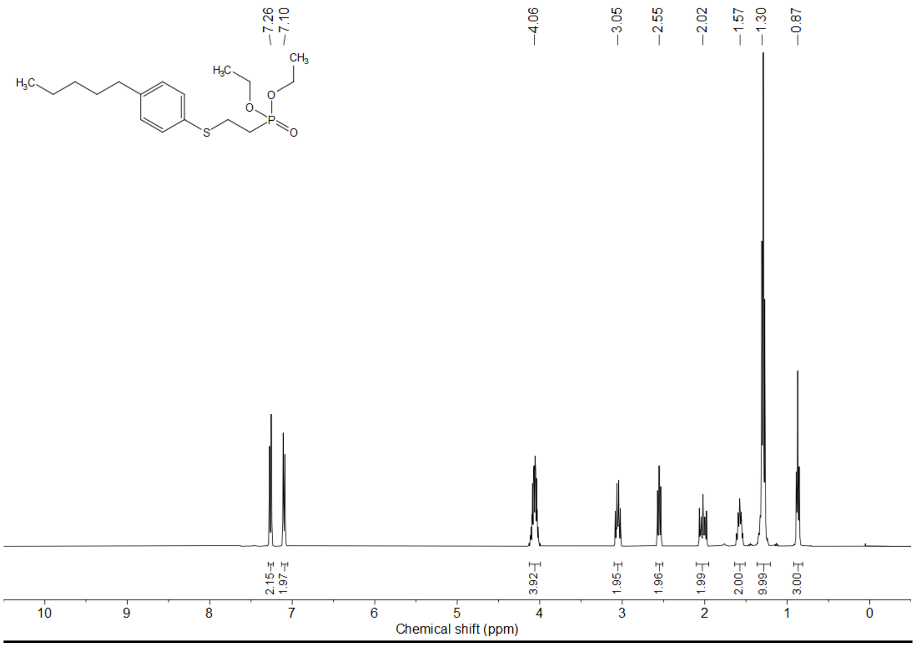

SI-14 ^{1}H-NMR

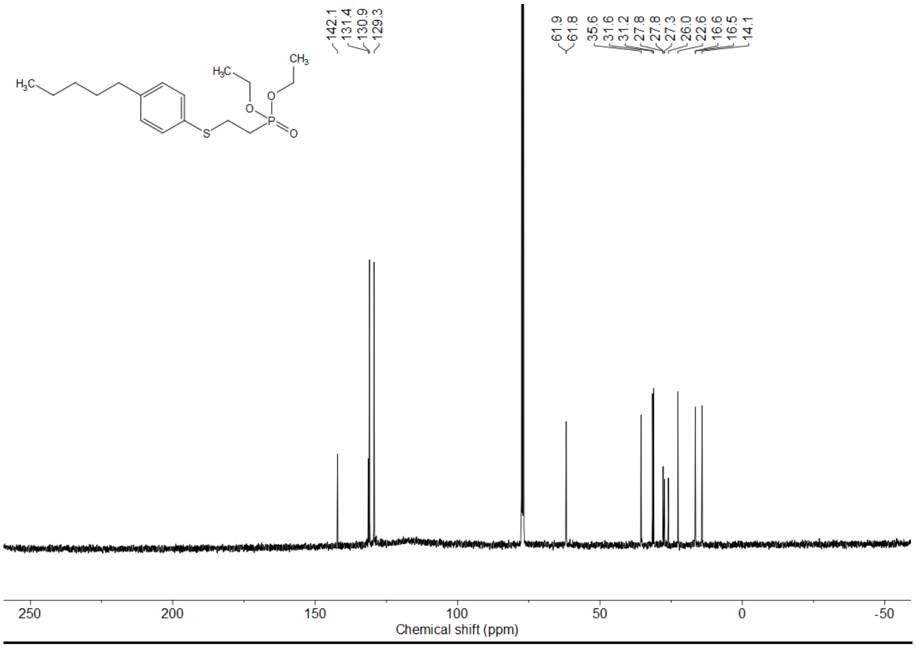

SI-14 ^{13}C-NMR

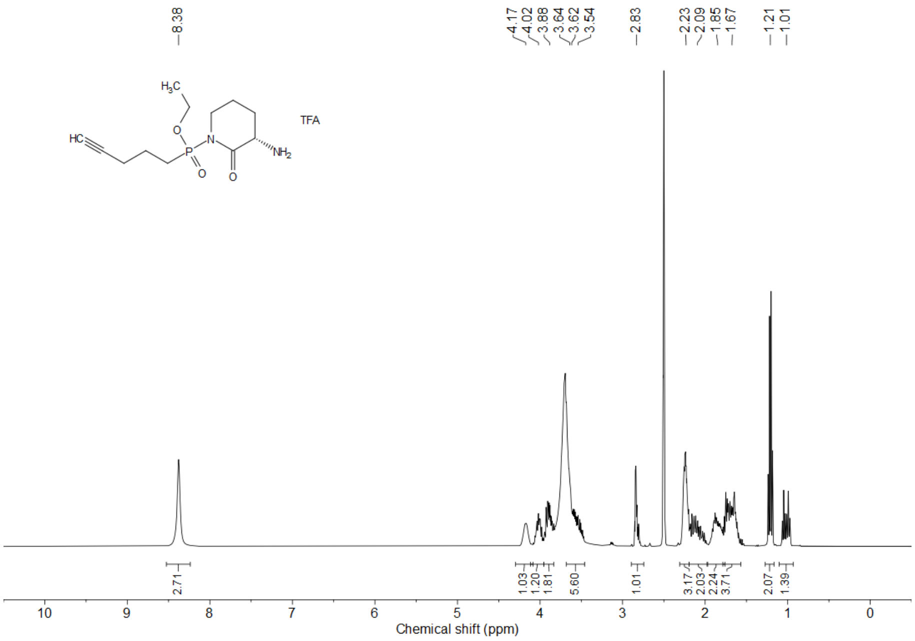

9 ^{1}H-NMR

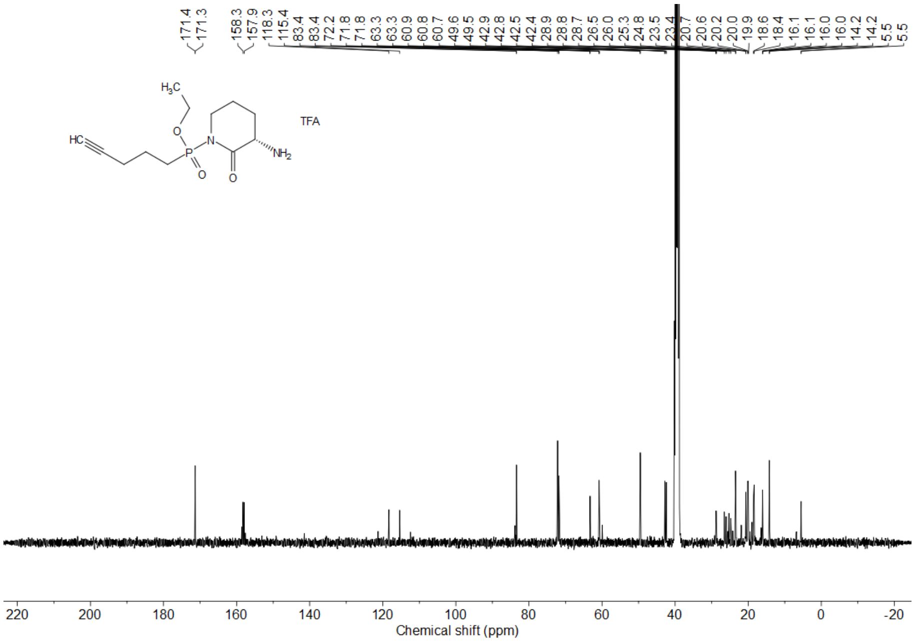

9 ^{13}C-NMR

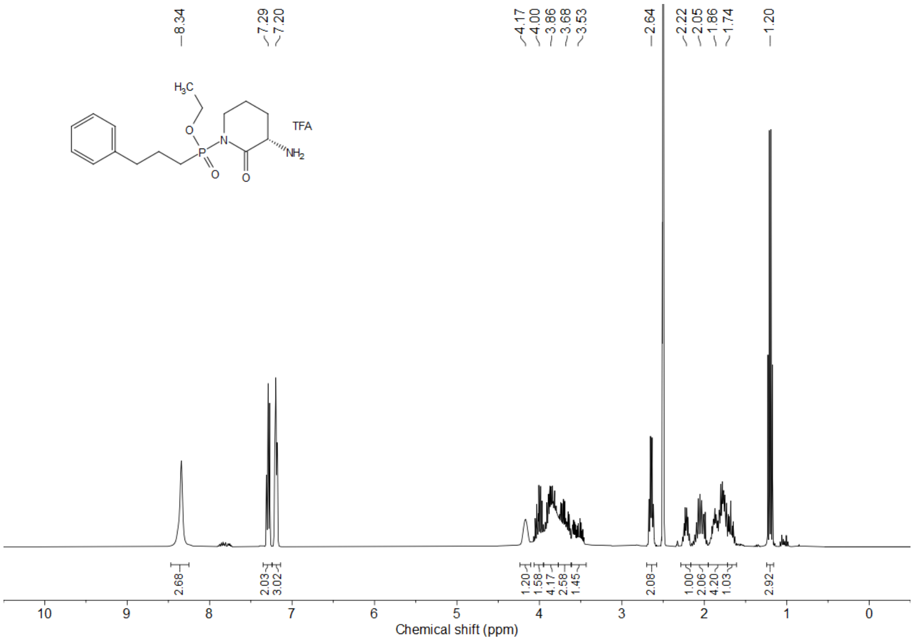

11 ^{1}H-NMR

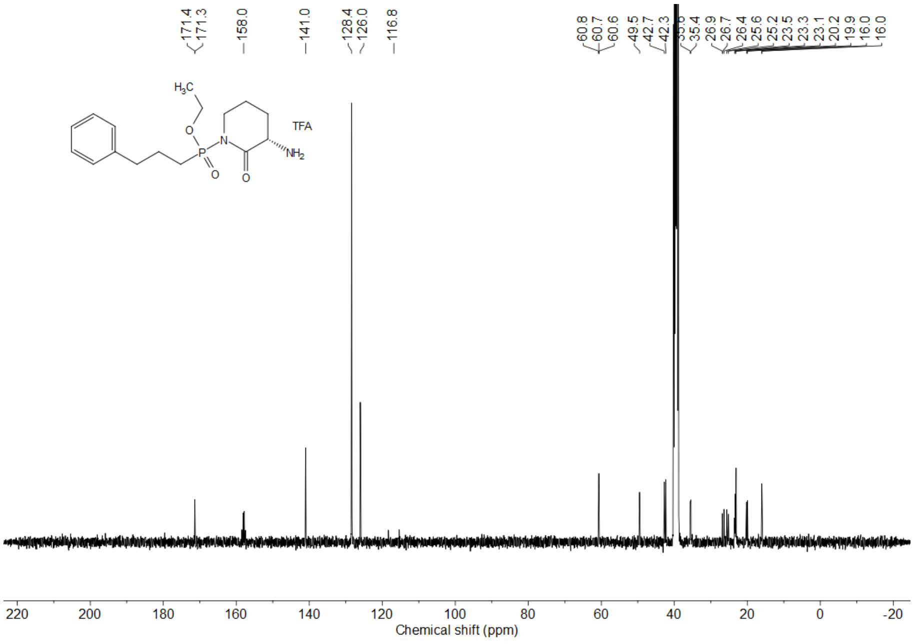

11 ^{13}C-NMR

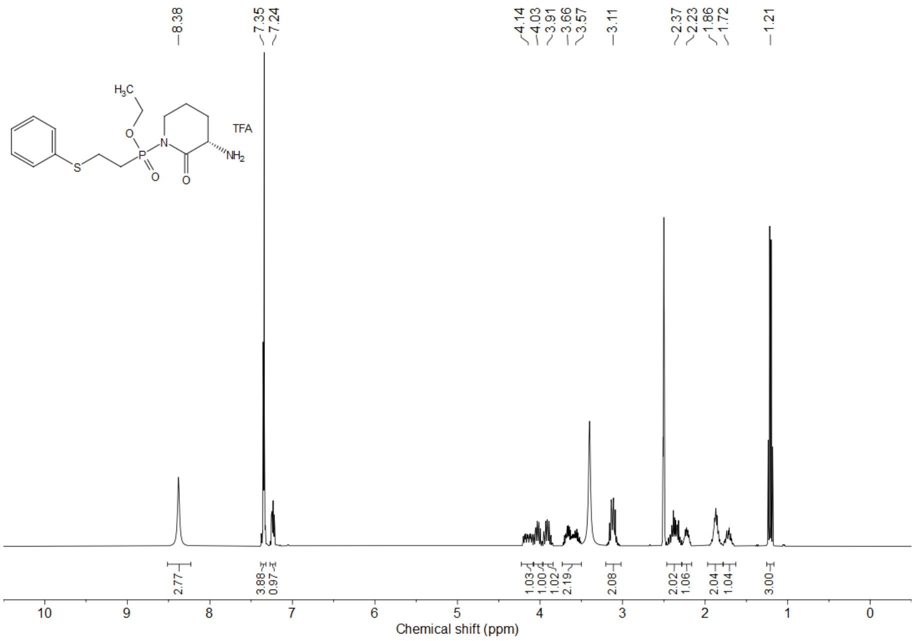

14 ^{1}H-NMR

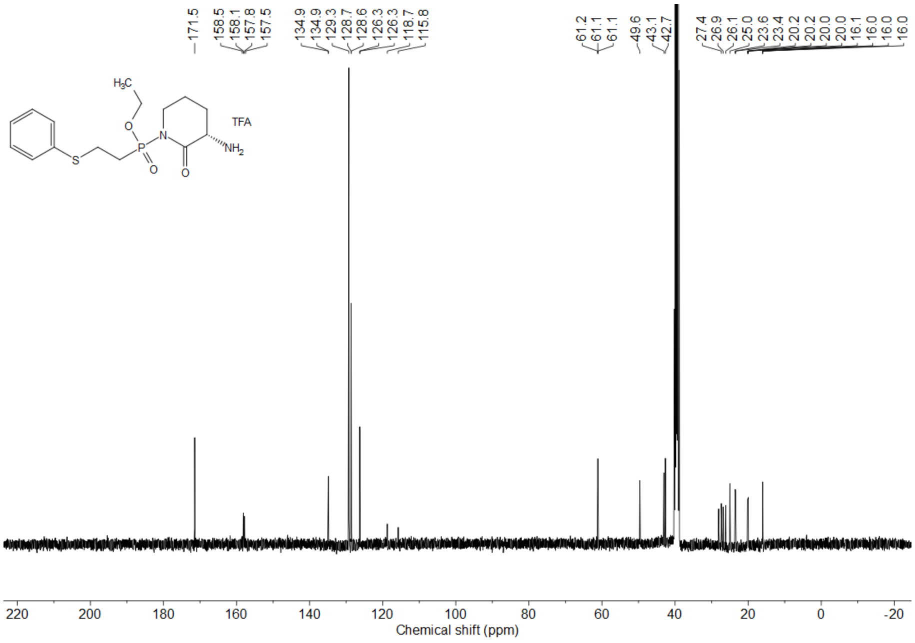

14 ^{13}C-NMR

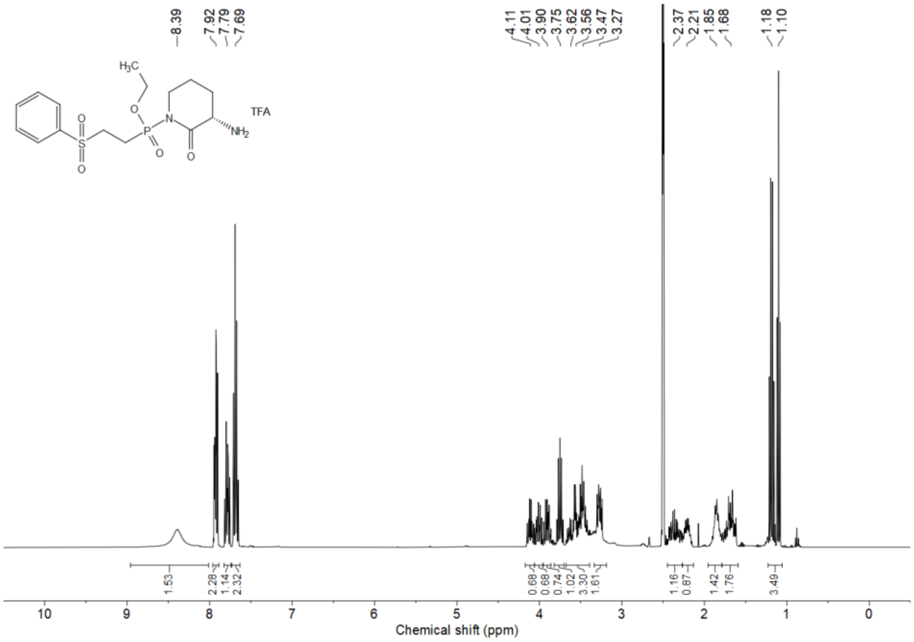

15 ^{1}H-NMR

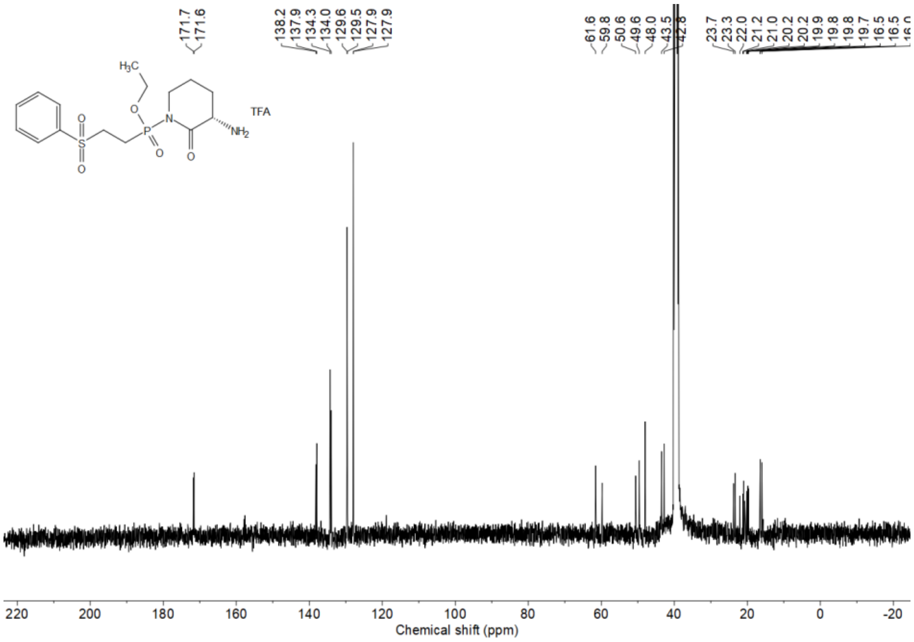

15 ^{13}C-NMR

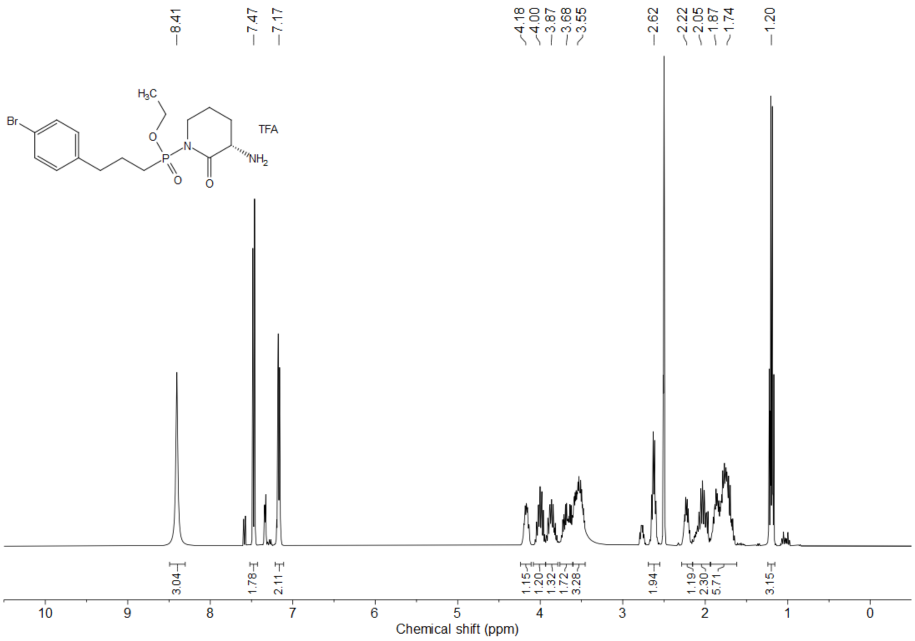

12 ^{1}H-NMR

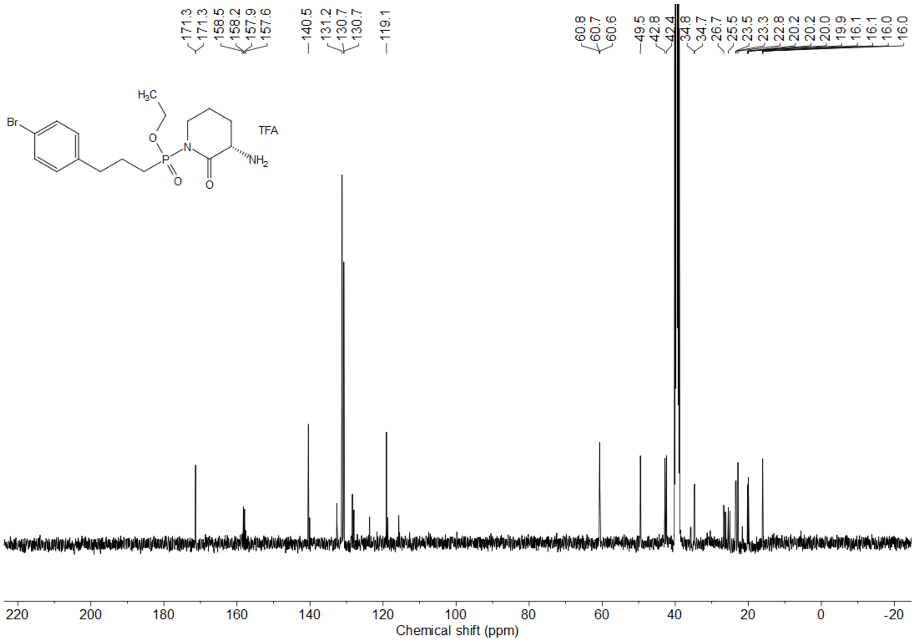

12 ^{13}C-NMR

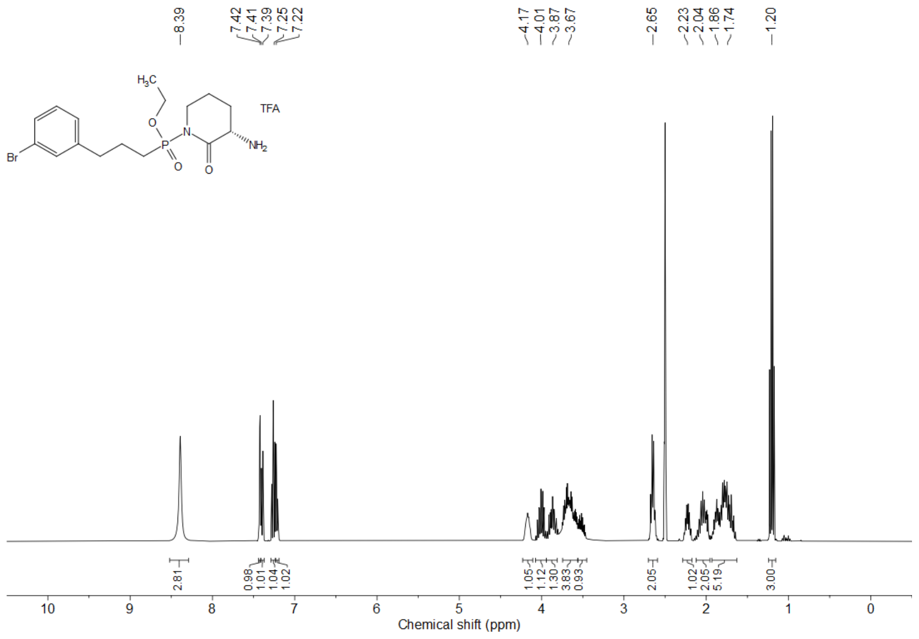

13 ^{1}H-NMR

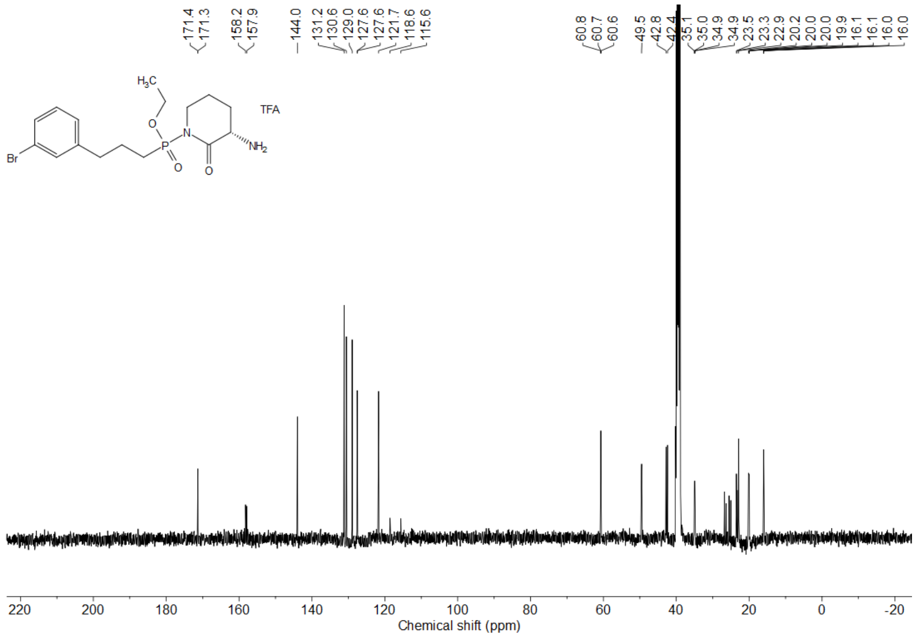

13 ^{13}C-NMR

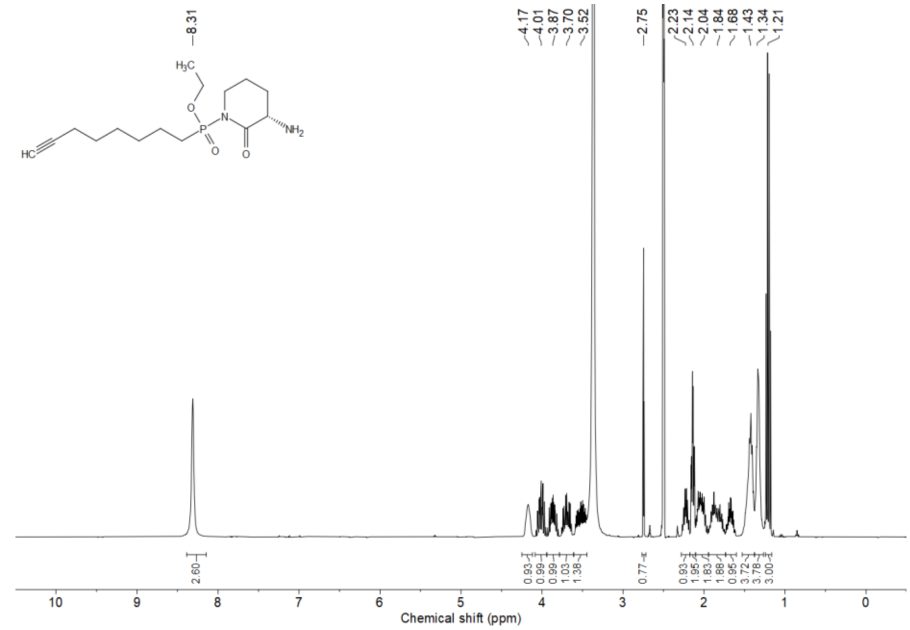

10 ^{1}H-NMR

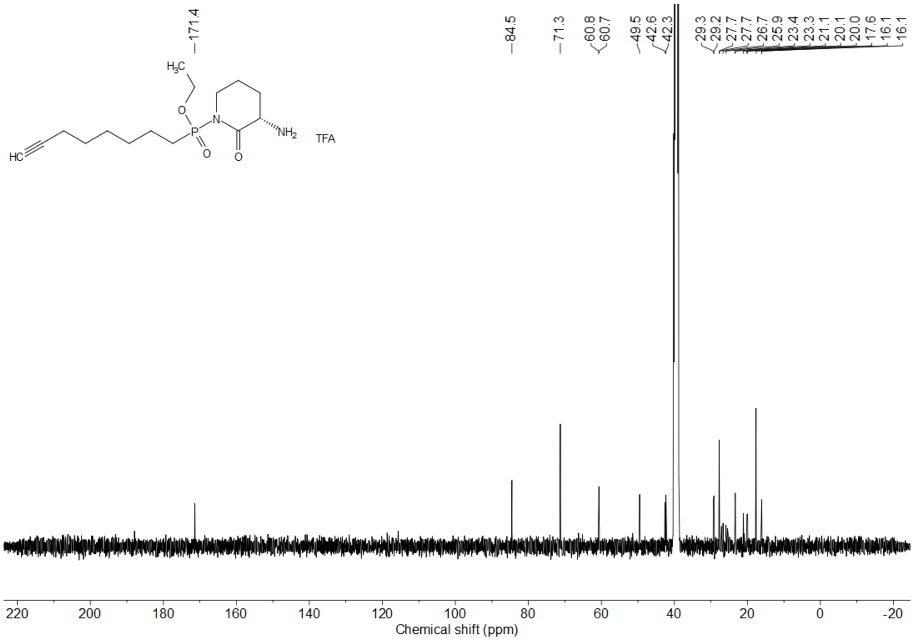

10 ^{13}C-NMR

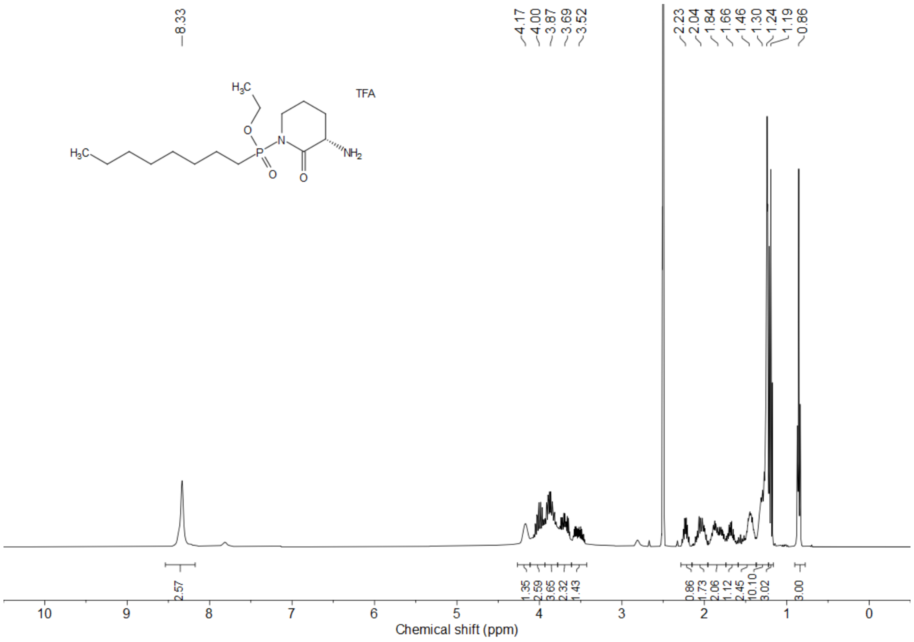

6 ^{1}H-NMR

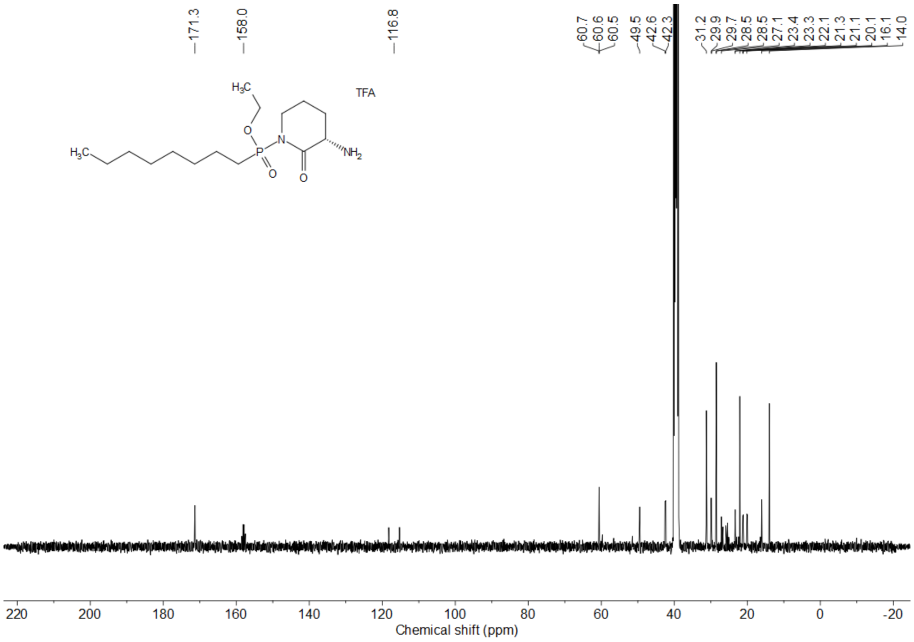

6 ^{13}C-NMR

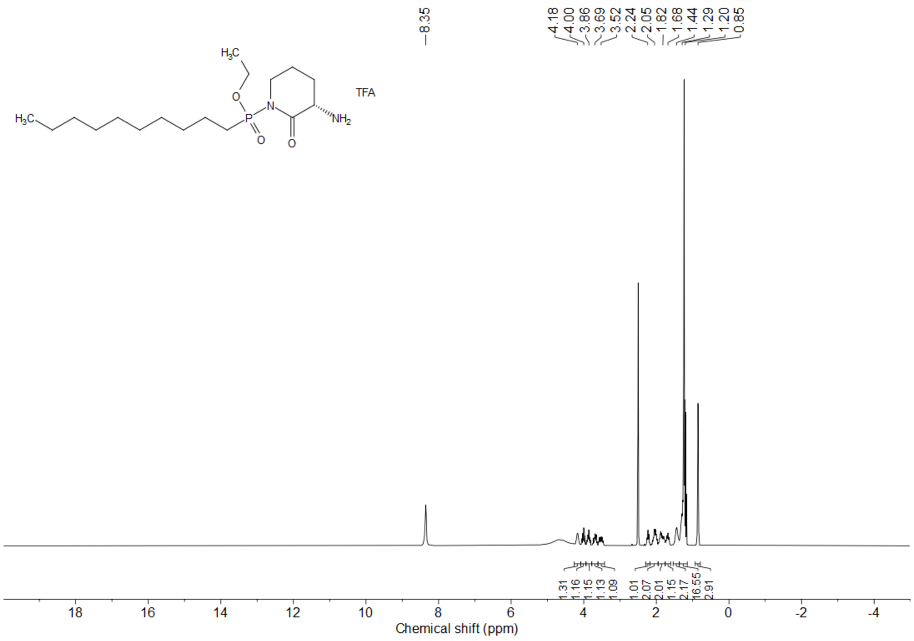

7 ^{1}H-NMR

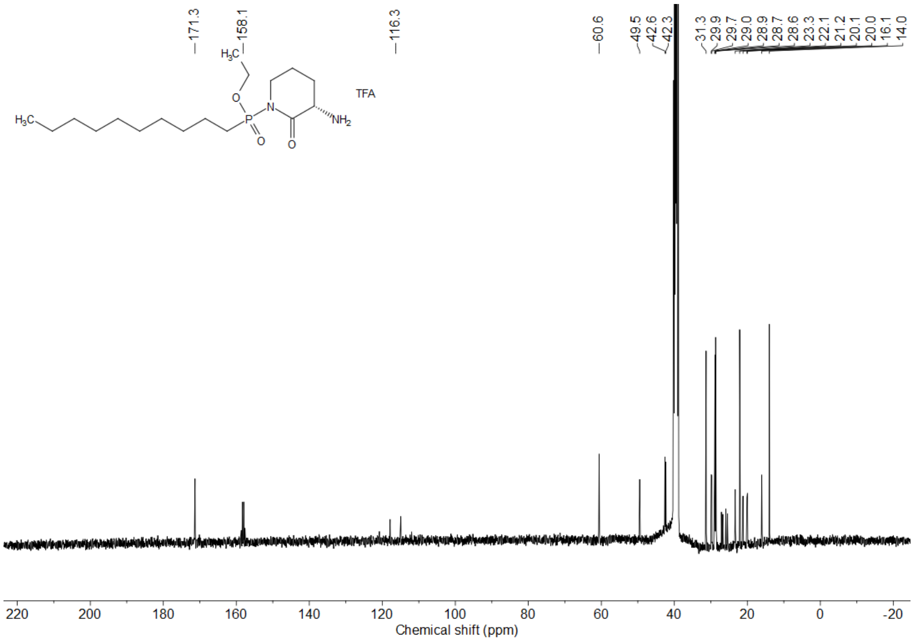

7 ^{13}C-NMR

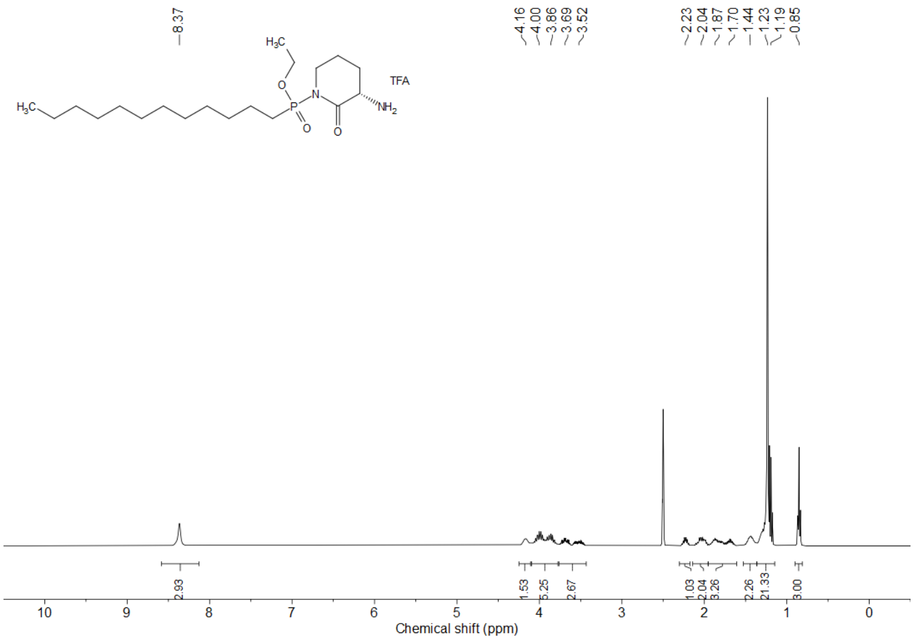

8 ^{1}H-NMR

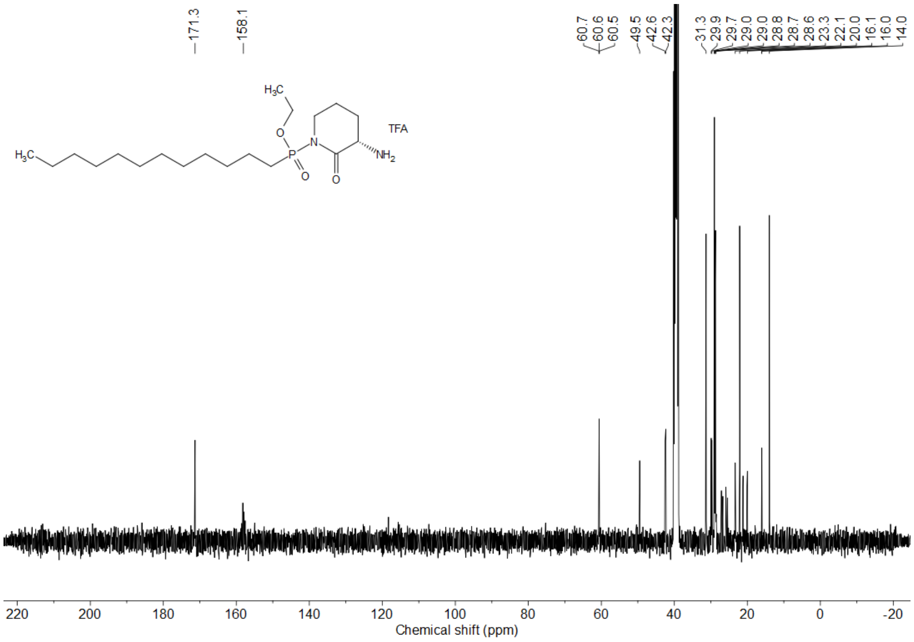

8 ^{13}C-NMR

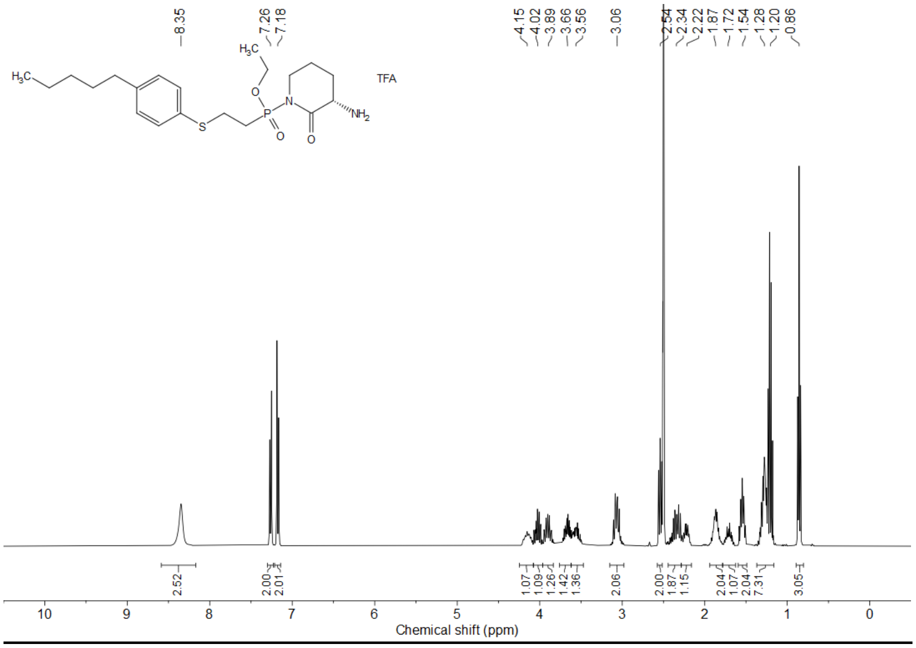

16 ^{1}H-NMR

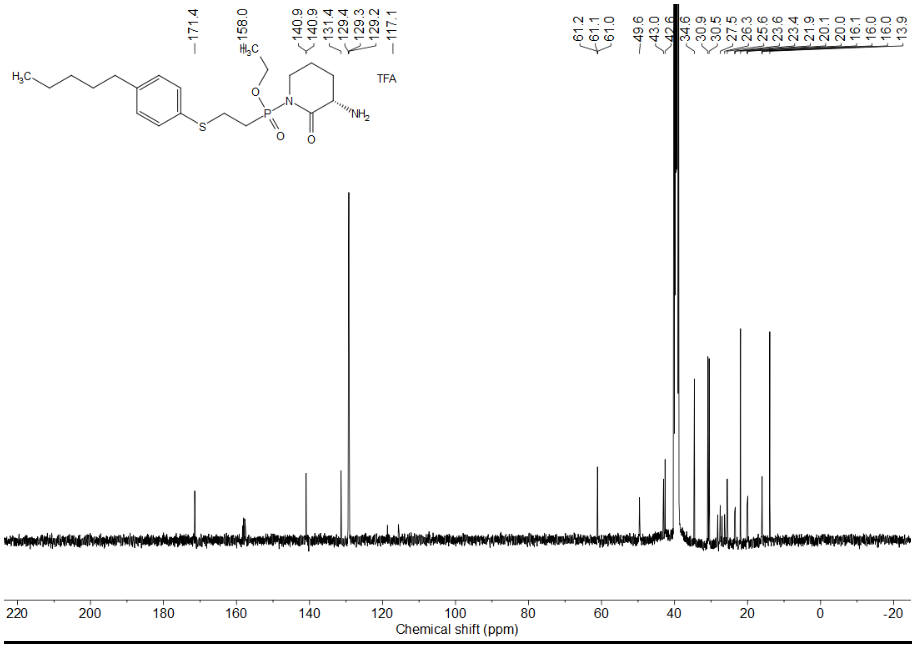

16 ^{13}C-NMR

Supplementary References

1 Abe, M. *et al.* First synthesis and determination of the absolute configuration of sulphostin, a novel inhibitor of dipeptidyl peptidase IV. *J Nat Prod* **67**, 999-1004 (2004).

2 Abe, M. *et al.* Development of a large-scale synthesis of sulphostin, a dipeptidyl peptidase IV inhibitor. *Org Process Res Dev* **9**, 570-576 (2005).

3 Yang, C. T. *et al.* Stereocontrolled C(sp3)-P bond formation with non-activated alkyl halides and tosylates. *Rsc Adv* **7**, 24652-24656 (2017).

4 Lopin, C., Gouhier, G., Gautier, A. & Piettre, S. R. Phosphonyl, phosphonothioyl, phosphonodithioyl, and phosphonotrithioyl radicals: Generation and study of their addition onto alkenes. *Journal of Organic Chemistry* **68**, 9916-9923 (2003).
