# Supplementary material for: Sulphostin-inspired N-phosphonopiperidones as selective covalent DPP8 and DPP9 inhibitors
Source: Nat Commun. 2025 Apr 3;16:3208. doi: 10.1038/s41467-025-58493-z (PMC11968843; doi:10.1038/s41467-025-58493-z)
Supplement: Supplementary file 2 — Description of Additional Supplementary Files [file 41467_2025_58493_MOESM2_ESM.docx]

**Description of Additional Supplementary Files**

**File name:** Supplementary Data 1

**Description:** Full list of proteins identified by ABPP, related to Fig. 3b

**File name:** Supplementary Data 2

**Description:** Full list of proteins identified by ABPP, related to Fig. 5b
