# Supplementary material for: Sulphostin-inspired N-phosphonopiperidones as selective covalent DPP8 and DPP9 inhibitors
Source: Nat Commun. 2025 Apr 3;16:3208. doi: 10.1038/s41467-025-58493-z (PMC11968843; doi:10.1038/s41467-025-58493-z)
Supplement: Supplementary file 5 — Reporting Summary [file 41467_2025_58493_MOESM5_ESM.pdf]

nature portfolio

Corresponding author(s): Markus Kaiser

Last updated by author(s): Mar 5, 2025

Reporting Summary

Nature Portfolio wishes to improve the reproducibility of the work that we publish. This form provides structure for consistency and transparency in reporting. For further information on Nature Portfolio policies, see our Editorial Policies and the Editorial Policy Checklist.

Statistics

For all statistical analyses, confirm that the following items are present in the figure legend, table legend, main text, or Methods section.

n/a	Confirmed	
☐	☒	The exact sample size (*n*) for each experimental group/condition, given as a discrete number and unit of measurement
☐	☒	A statement on whether measurements were taken from distinct samples or whether the same sample was measured repeatedly
☐	☒	The statistical test(s) used AND whether they are one- or two-sided<br>*Only common tests should be described solely by name; describe more complex techniques in the Methods section.*
☒	☐	A description of all covariates tested
☐	☒	A description of any assumptions or corrections, such as tests of normality and adjustment for multiple comparisons
☐	☒	A full description of the statistical parameters including central tendency (e.g. means) or other basic estimates (e.g. regression coefficient) AND variation (e.g. standard deviation) or associated estimates of uncertainty (e.g. confidence intervals)
☐	☒	For null hypothesis testing, the test statistic (e.g. *F*, *t*, *r*) with confidence intervals, effect sizes, degrees of freedom and *P* value noted<br>*Give P values as exact values whenever suitable.*
☒	☐	For Bayesian analysis, information on the choice of priors and Markov chain Monte Carlo settings
☒	☐	For hierarchical and complex designs, identification of the appropriate level for tests and full reporting of outcomes
☒	☐	Estimates of effect sizes (e.g. Cohen's *d*, Pearson's *r*), indicating how they were calculated

Our web collection on statistics for biologists contains articles on many of the points above.

Software and code

Policy information about availability of computer code

Data collection	All instruments were controlled with commercial software provided by the manufacturer.<br>Mass spectrometer were operated using Xcalibur software v3.0.63 and v4.7.69.37.<br>Diffraction data were collected at PXII/X10SA (SLS, Villigen, Switzerland) for DPP4:1 as well as DPP9 Ser730Ala:1 and at ID23-1 (ESRF, Grenoble, France) for the DPP9:16.
Data analysis	Graph-Pad Prism (v10.2.3 and v8.0.1)<br>MaxQuant (v2.0.3.0 and v2.5.2.0)<br>Zen black (v2.3)<br>Duolink ImageTool Setup 1.0.1.2<br>UniDec software (v6.0.4)<br>autoPROC, XDS (BUILT = 20200417 or 20230630)<br>autoPROC, AIMLESS (v0.7.7)<br>phaser (v2.8.3)<br>COOT (v0.8.9.2 or v0.9.8)<br>REFMAC (v5.8.0267, v5.8.0430 or v5.8.0419)<br>CORINA (v4.4.00026)<br>UCSF ChimeraX (v1.7.1)

For manuscripts utilizing custom algorithms or software that are central to the research but not yet described in published literature, software must be made available to editors and reviewers. We strongly encourage code deposition in a community repository (e.g. GitHub). See the Nature Portfolio guidelines for submitting code & software for further information.

Data

Policy information about availability of data

All manuscripts must include a data availability statement. This statement should provide the following information, where applicable:

- Accession codes, unique identifiers, or web links for publicly available datasets
- A description of any restrictions on data availability
- For clinical datasets or third party data, please ensure that the statement adheres to our policy

The structural datasets generated in this study have been deposited in the ProteinDataBank repository under the accession codes 9GOH (DPP4:1) [https://doi.org/10.2210/pdb9goh/pdb], 9GON (DPP9:1) [https://doi.org/10.2210/pdb9gon/pdb], 9GOC (DPP9 Ser730Ala:1) [https://doi.org/10.2210/pdb9goc/pdb], and 9GOD (DPP9:16) [https://doi.org/10.2210/pdb9god/pdb]. The crystal structures used in this study are available in the ProteinDataBank repository under the accession codes 1PFQ (DPP4) [https://doi.org/10.2210/pdb1pfq/pdb], 6EOO (DPP8) [https://doi.org/10.2210/pdb6eoo/pdb] and 6EOQ (DPP9) [https://doi.org/10.2210/pdb6eoq/pdb]. The mass spectrometry proteomics data have been deposited to the ProteomeXchange Consortium via the PRIDE partner repository with the dataset identifier PXD058896 [https://proteomecentral.proteomexchange.org/cgi/GetDataset?ID=PXD058896]. The processed chemoproteomic data are available in Supplementary Data 1 and 2. Source data are provided with this paper.

Research involving human participants, their data, or biological material

Policy information about studies with human participants or human data. See also policy information about sex, gender (identity/presentation), and sexual orientation and race, ethnicity and racism.

Reporting on sex and gender	This study did not involve human research participants.
Reporting on race, ethnicity, or other socially relevant groupings	This study did not involve human research participants.
Population characteristics	This study did not involve human research participants.
Recruitment	This study did not involve human research participants.
Ethics oversight	This study did not involve human research participants.

Note that full information on the approval of the study protocol must also be provided in the manuscript.

Field-specific reporting

Please select the one below that is the best fit for your research. If you are not sure, read the appropriate sections before making your selection.

☒ Life sciences ☐ Behavioural & social sciences ☐ Ecological, evolutionary & environmental sciences

For a reference copy of the document with all sections, see nature.com/documents/nr-reporting-summary-flat.pdf

Life sciences study design

All studies must disclose on these points even when the disclosure is negative.

Sample size	Sample sizes were chosen based on 'standard' sample size in literature and preliminary results. No statistical methods for sample size calculation were used. The sample sizes were sufficient to replicate the experiments and assess technical and biological viability of the result.
Data exclusions	No data were excluded.
Replication	All experiments were performed with sufficient biological or technical replicates in order to demonstrate statistical significance.<br>The exact number of replicates for each experiment is given in the respective figure legend or method section (n=4 biologically independent replicates for chemoproteomic experiments; n=3 technical replicates for enzyme inhibition assays, n=3 biologically independent replicates for PLA assays; n=9, 17 or 18 independent biological replicates for cell viability assays). All attempts at replication were successful.
Randomization	Samples (protein aliquots or cultured cells) were randomly allocated to the different experiments.
Blinding	Blinding is not applicable during the experimentation and data collection as the results were quantitative and did not require subjective interpretations. All biological samples were equally processed and analyzed.

Reporting for specific materials, systems and methods

We require information from authors about some types of materials, experimental systems and methods used in many studies. Here, indicate whether each material, system or method listed is relevant to your study. If you are not sure if a list item applies to your research, read the appropriate section before selecting a response.

Materials & experimental systems

n/a	Involved in the study
☐	☒ Antibodies
☐	☒ Eukaryotic cell lines
☒	☐ Palaeontology and archaeology
☒	☐ Animals and other organisms
☒	☐ Clinical data
☒	☐ Dual use research of concern
☒	☐ Plants

Methods

n/a	Involved in the study
☒	☐ ChIP-seq
☒	☐ Flow cytometry
☒	☐ MRI-based neuroimaging

Antibodies

Antibodies used

-anti-BRCA2, mouse, monoclonal IgG (purchased from R&D, Cat No#MAB2476, Clone:234403, RRID:AB_2259370)
-anti-DPP9, goat, polyclonal (self-made, RRID:AB_2889071, previously published in Justa-Schuch etal. (2016) https://doi.org/10.7554/eLife.16370 and Bolgi et al. (2022) https://doi.org/10.15252/embr.202154136)

Validation

-The anti-BRCA2 Ab was validated by silencing in Bolgi et al. (2022) https://doi.org/10.15252/embr.202154136 (Figure 1, Supplementary Figure 1 by western blotting and PLA)
On the manufacturers webside, further publications (13) are mentioned: https://www.rndsystems.com/products/human-brca2-antibody-234403_mab2476#product-reviews

-This anti-DPP9 Ab was validated via IF on DPP9 silenced and also on DPP9KO cells.

Eukaryotic cell lines

Policy information about cell lines and Sex and Gender in Research

Cell line source(s)

HEK293 cells were obtained from Cytion (300192, Lot: 300192-1119; p29).
HeLa Flp-In T-REx WT were a kind gift from Prof. Matthias Hentze.
The CRISPR-Cas9 system was used for knock out DPP9, generating a HeLa DPP9KO cell line cells as described in Heß et al. (2024) https://doi.org/10.1016/j.bbadis.2024.167133

Authentication

The cell lines used were not authenticated

Mycoplasma contamination

The cell lines used were tested negative for mycoplasma contamination.

Commonly misidentified lines
(See ICLAC register)

No commonly misidentified cell lines were used in the study

Plants

Seed stocks

No plants were involved in this study.

Novel plant genotypes

No plants were involved in this study.

Authentication

No plants were involved in this study.
